# Shedding Light on the Capabilities of Heteroditopic Mechanically Interlocked Molecules in Ion-Pair Sensing

**DOI:** 10.1021/acs.jpca.5c08037

**Published:** 2026-02-05

**Authors:** Fábio J. Amorim, Felipe R. F. Pagliarini, Renato L. T. Parreira, Giovanni F. Caramori

**Affiliations:** † Departamento de Química, 28117 Universidade Federal de Santa Catarina, Campus Universitário Trindade, 88040-900, Florianópolis, SC, Brazil; ‡ Núcleo de Pesquisas em Ciências Exatas e Tecnológicas, 92917 Universidade de Franca, 14404-600, Franca, SP, Brazil

## Abstract

Heterotopic mechanically interlocked molecules contain different binding sites within their structure, allowing them to recognize specific ion pairs (cations and anions) with a high affinity. The employment of heteroditopic receptors offers advantages over monotopic analogues, in general, being composed of both cation and anion binding sites. The present study elucidates the electronic structure-based recognition of anions and cations of a heteroditopic [2]­catenane, **IO**. Spherical cations and anions have been employed. The structure of **IO** was modified by replacing its original oxygen atoms of the crown-ether moiety by sulfur atoms and σ–hole donor iodines by −Te–CH_3_ groups leading to the modified [2]­catenanes **IS** and **TeO**, respectively. Energy decomposition analysis (EDA) and natural orbital for chemical valence reveals that the cations exhibit the strongest interaction with the binding pockets of all structures, with Cu^+^, Li^+^, and Ni^2+^ presenting the most stabilizing values, $\Delta E_{IO}^{tot}$ = −198.2, −175.1, and −653.4 kcal·mol^–1^, and $\Delta E_{IS}^{tot}$ = −226.4, −154.0, −702.5 kcal·mol^–1^, respectively. In contrast, anion recognition presented to be significantly lower, being purely dependent on the strength of the σ–hole donors and the size of the applied anion, with Cl^–^ exhibiting the most stable interaction, where $\Delta E_{IO}^{tot}$ = −109.9 kcal·mol^–1^. It was also found that the anion recognition for this particular molecule does not affect the cation recognition, significantly. The EDA results confirm that changing from a harder (O) to a softer (S) interactive environment will have considerable impact on cation recognition, thereby demonstrating the pivotal role, following the *size match rule*.

## Graphical abstract

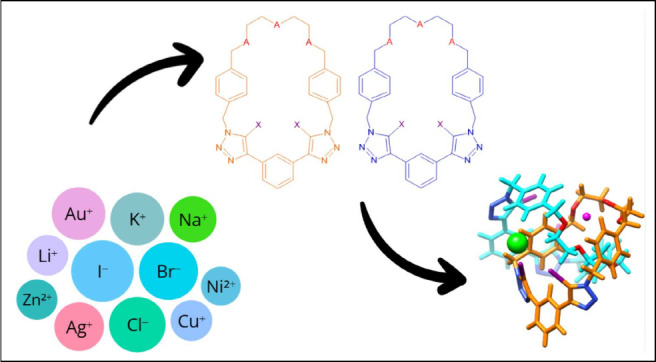

## 1 Introduction

It is well-established in literature that mechanically interlocked molecules (MIMs) exhibit a wide range of movements, due to their specific topologies (allowing to "distort" without disconnecting its fragment components or breaking the chemical bonds between its atoms), which can be applied in order to produce molecular-level machines.^1,2^ Such molecular machines are, in many cases, dependent on stimuli such as light irradiation or chemical input in order to be activated.^2^

In the domain of supramolecular chemistry, MIMs can be engineered to function as a molecular framework for ionic recognition. It is directly related with their structural and chemical features, which yield promising and efficient species capable of binding anions and cations.^3^ Typically, these species establish noncovalent interactions, either independently or in conjunction, thereby enhancing the interaction with ionic species. For instance, the mechanisms of interaction include hydrogen bonds,^3–7^ metal ion coordination,^8^ and halogen bonds.^9,10^ The generation of 3D cavities and clefts, tailored to specific geometric requirements, facilitates the host–guest interaction with charged species.^1^

Charged species are ubiquitous, playing a crucial role in a variety of processes in fields as diverse as chemistry, biology, medicine, the environment, and industry.^11^ In the context of anion recognition, noncovalent bonding can occur through the σ – hole interactions,^12^ a phenomenon that has been reported to be more effective than hydrogen bonding alone.^13^ This has also been confirmed recently, showing how important σ – hole interactions are in recognizing anions with different geometries.^14^ The interaction of cations with mechanically bonded sites has also been shown to enhance their binding strength, resulting in more favorable interactions compared to analogous noninterlocked ligands.^11,15,16^

The development of strong and selective systems for charged species remains a considerable challenge, not only for MIMs but also for supramolecular host–guest chemistry, in general. Heteroditopic receptors can fill this gap, because they contain domains that can recognize cations and anions at the same time. It has been shown that they work better than monotopic analogues.^17–21^ The investigation of cooperative effects associated with ion-pair binding represents a promising avenue for the design of heteroditopic receptors. This emerging field has significant potential applications, including the development of salt extraction and solubilization^22–26^ and membrane transport.^27,28^

Heteroditopic receptors are typically comprised of one or more cation- and anion-binding sites, which can be located in close proximity to each other or can be distributed over a wide spatial extent, leading to general classification of (i) contact ion-pair or (ii) separated ion-pair receptors. MIMs represent a paradigm shift in heteroditopic host design. They move beyond the limitations of covalent architecture by leveraging topological constraints to create systems that are not just preorganized, but also adaptive, highly cooperative, and stimuli-responsive.^21,29^ MIMs can therefore be employed as heteroditopic ion-pair hosts, offering distinct advantages over traditional acyclic or macrocyclic hosts due to their unique structural and topological features. This resulting improvement is termed as the *mechanical bond effect*, where the preorganized and solvent-shielded cavities of MIMs offer a unique opportunity to design three-dimensional binding sites for guest encapsulation. The degree of preorganization is directly correlated with the strength of ion-pair binding, suggesting that the highest degree of preorganization results in the strongest ion-pair binding. The present study illuminates the physical basis of the ion-pair sensing capability of the heteroditopic catenane (**IO**). It verifies the influence of the nature of the ions on each other as well as the role of the mechanical bond and different σ-hole donors in ion recognition. The study also highlights the presence of cooperativity in the process. In this sense, Beer’s heteroditopic [2]­catenane, **IO**, was used as a reference structure,^30^ which is comprised by the entanglement of two oligo­(ethylene glycol)-functionalized macrocycles as highlighted in Figure 1. The reference structure **IO** was modified by replacing the oxygen atoms by sulfur in the cation-interacting moiety (crown ether cavity). It led to heteroditopic [2]­catenane **IS**. The role of different σ – hole donors has been explored by substituting the iodine atoms involved in the anion recognition by a methyltellanyl group (-Te–CH_3_), leading to the [2]­catenane **TeO** (Figure 1). 3D structures are available in the Supporting Information material (Figure S1a-b). A set of spherical ions has been considered, including transition and d group metal cations (Cu^+^, Ag^+^, Au^+^, Ni^2+^, and Zn^2+^) alongside halogen anions (Cl^–^, Br^–^, and I^–^). The ${{\mathrm{PF}}_{6}}^{\text{−}}$ counterion has been utilized in systems comprising M^2+^ cations with a view to maintaining overall electrical neutrality and ensuring electrostatic comparability. This approach is intended to circumvent electrostatic biases in the energy decomposition analysis. All calculations analyzing the interaction between the [2]­catenane and Cl^–^ and Br^–^ anions, in **IO**-Cl^–^ and **IO**-Br^–^, as well as the performed studies replacing the original σ – donor iodine atoms for −Te–CH_3_ groups, in **TeO**-Cl^–^ were performed with the usage of Li^+^ as the interacting cation, since we aimed at further exploring purely the anion recognition capability of the [2]­catenane and the main influencing factors for the interaction. Energy Decomposition Analysis and Natural Orbital for Chemical Valence model, EDA-NOCV, has been used as a quantitative tool in a fragmentation scheme to provide a deeper insight into the role played by both applied cation and anion as well as the role played by the interacting environment in ion-pair recognition.

**Figure 1:**
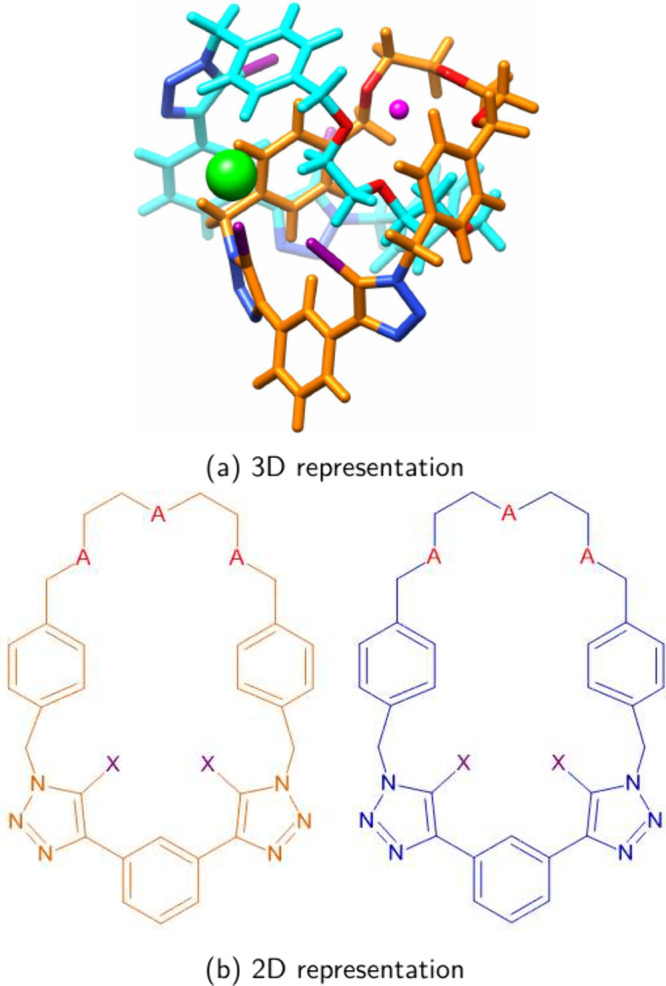
(a) The 3D representation of all overall structures of the studied [2]­catenanes contains a cation (highlighted as the pink sphere) situated in the crown ether moiety (oxygen atoms highlighted in red) and an anion (highlighted as the green sphere). (b) 2D structure without the inclusion of ions; In **IO**, X = I and A = O; In **IS**, X = I and A = S; In **TeO**, X = −Te–CH_3_ and A = O.

## 2 Computational Methods

The crystallographic structure of **IO** was employed as a reference model^31^ to build up structures **IS** and **TeO**, in which the oxygen atoms from the crown ether portion of the [2]­cateanene were replaced by sulfur atoms and the iodine σ – hole donor atoms were replaced by −Te–CH_3_ groups, respectively. The cations were positioned in the cleft containing an oligo­(ethylene glycol)-functionalized macrocycle as this configuration has been shown to be optimal based on experimental data. Subsequently, the entire structure was fully optimized without constraints using the Generalized Gradient Approximation (GGA) Density Functional Theory (DFT) functional of Becke and Perdew,^32,33^ BP86, in conjunction with the application of Grimme’s dispersion correction, DFT-D3,^34–36^ and the basis set LANL2DZ.^37^ Auxiliary basis sets were also employed by following the resolution of the identity approximation (RI). It has been considered for both the Coulomb (def2/J)^38^ and the exchange correlation integrals (RIJCOSX).^39^ All of the above structures were fully optimized without constraints by using the same level of theory. All optimized geometries correspond to minima structures on the potential energy surface due to the absence of imaginary eigenvalues on the Hessian matrix. All geometry optimizations were performed using the software Orca5.0.4 ^40^ and considering implicit solvation with SMD^41^ model applying chloroform as solvent. The molecular graphics images were created using the UCSF Chimera package.^42^

The nature of the host–guest interaction between the MIMs and the chosen ions was analyzed with the Energy Decomposition Analysis and Natural Orbital for Chemical Valence model (EDA-NOCV)^43,44^ approach as implemented in AMS-ADF sofware.^45^ The present analysis was conducted using the same theoretical framework previously outlined but now applied with an all-electron basis set, TZ2P.^46,47^ To support the reasoning behind the EDA-NOCV results, Hirshfeld charge analysis^48^ was also considered utilizing the same level of theory and dispersion correction previously mentioned by in conjunction with the def2-SVP basis set.^49^ The EDA-NOCV has been employed focusing to shed light on the bonding situations involving the ability of [2]­catenanes to recognize anions and cations (ion-pair), to quantify the contributions of halogen bonds for anion recognition, and to quantify the contributions of different sizes of the applied cations on this process, by means of different partition schemes adopted, this being an insightful method that has been used for host–guest recognition interactions in a number diverse studies.^3,50^ In this method, the total interaction energy, ΔE^tot^, between the two interacting fragments is decomposed according to eq 1, in which the total energy is made up of four main terms.

1 $$\Delta E^{tot}=\Delta E^{elstat}+\Delta E^{Pauli}+\Delta E^{oi}+\Delta E^{disp}$$

In this method, the total interaction energy, ΔE^tot^, between two or more interacting fragments is decomposed according to eq 1, in which ΔE^tot^ is decomposed into physically meaningful terms: electrostatic (ΔE^elstat^), Pauli’s repulsion (ΔE^Pauli^), orbital interaction (ΔE^oi^), and dispersion (ΔE^disp^).

The electrostatic component, ΔE^elstat^, represents the quasiclassical electrostatic interaction between the unperturbed charge distributions of the deformed fragments. The Pauli repulsion, ΔE^Pauli^, accounts for the repulsion related to the direct Pauli exclusion principle of both fragment orbitals, which must respect the wave function antisymmetry. The orbital interaction energy, ΔE_oi_, represents both the charge transfer and the orbital polarization of the inner fragment. This last term is further decomposed into the pairwise contribution of the interacting fragment orbitals according to the deformation density channels. The EDA-NOCV method also estimates the density-flow symmetry and direction and its energy contribution. The ΔE_disp_ energy considers dispersion corrections, as suggested by Grimme et al. Further details about the EDA-NOCV method can be found in the literature.^43,51^

## 3 Results and Discussion

### 3.1 Geometric Parameters

The geometry optimization process led the ions to acquire their most stable sites, with the anions assuming a position in close proximity to the σ – hole donor atoms, while the cations moved toward a position within the crown ether portion of the catenane. Table 1 provides an overview of the most significant geometric parameters associated with both cation and anion interactions, including the distances between both ions, dC···A, and the distances from their nearest neighboring atoms belonging to different portions of the [2]­catenane (see insets in Figure 2) are named from **d1** to **d7**. For the applied M^2+^ cations the shortest distance between the cations and the counterion, ${{\mathrm{PF}}_{6}}^{\text{−}}$ , was named as **dCI** (Figure S2). The angles that resulted from both σ – hole donor atoms interacting with the applied anion were also obtained and denominated as C-σ_1_ ··· X^–^ and C-σ_2_ ··· X^–^.

**Figure 2:**
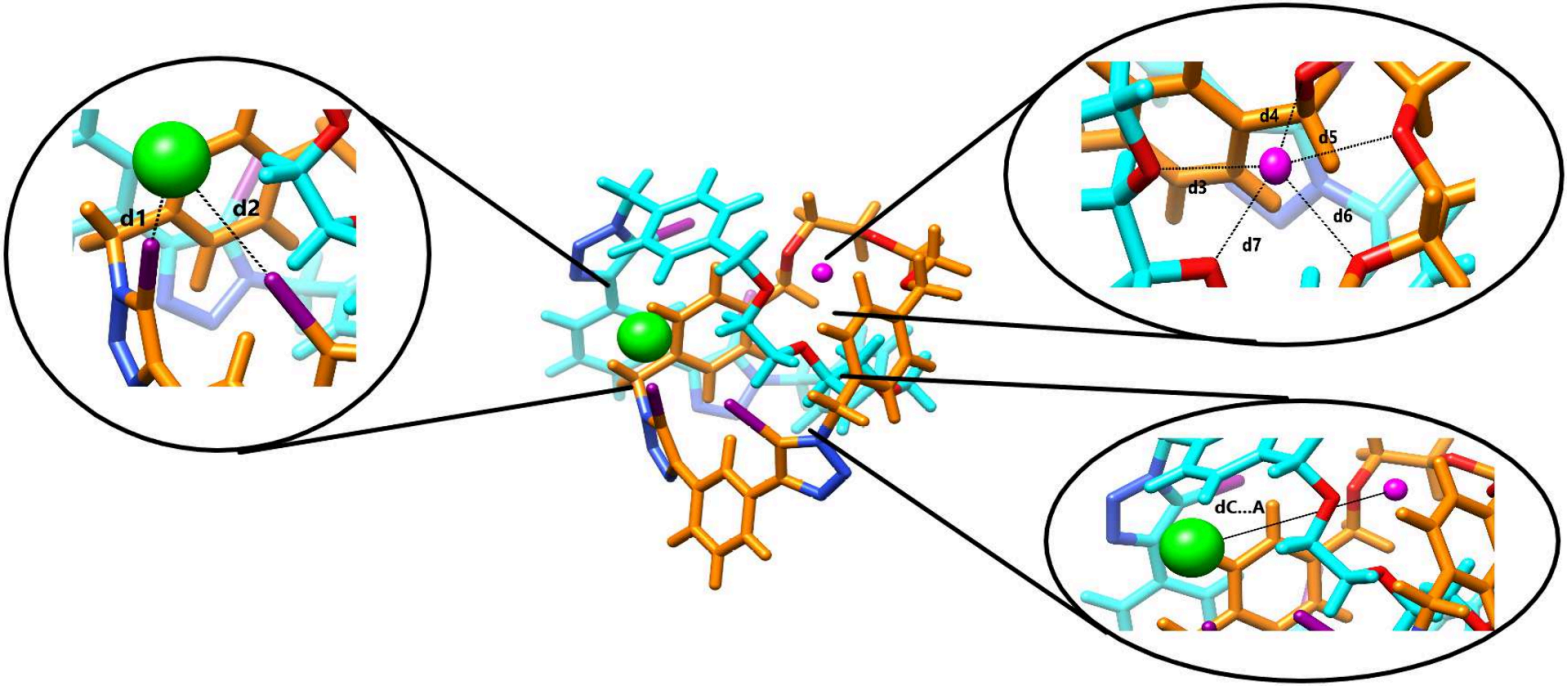
Geometrical parameters involved in the recognition of the anion and cations applied. The distances **d1** and **d2** are the shortest distances between the σ – donor and acceptor, while **d3** to **d7** depict the shortest distances between the cation and the oxygen/sulfur atoms.

**Table 1:** Selected Geometric Parameters for the Optimized Structures of **IO**-M^+^| $M^{2+}{{\mathrm{PF}}_{6}}^{\text{−}}X^{\text{−}}$ , **IS**-M^+^ | $M^{2+}{{\mathrm{PF}}_{6}}^{\text{−}}X^{\text{−}}$ , and **TeO**-M^+^X^–^ (X^–^ = Cl^–^, Br^–^, I^–^ ; M^+^| $M^{2+}{{\mathrm{PF}}_{6}}^{\text{−}}$ = Li^+^, Na^+^, and K^+^, Cu^+^, Ag^+^, Au^+^, Ni^2+^, and Zn^2+^)

	Angles (deg)		Distances (Å)								
	C-σ_1_ ··· X^–^	C-σ_2_ ··· X^–^	d1	d2	d3	d4	d5	d6	d7	dCI	dC ··· A
**IO-**Li^+^Cl^–^	169.6^a^	167.8	3.15	3.10	1.98	2.11	2.16	2.16	2.01	-	6.44
**IO**-Li^+^Br^–^	170.7	170.0	3.31	3.27	1.99	2.26	2.07	2.22	1.99	-	6.50
**IO**-Li^+^I^–^	171.4	171.0	3.63	3.54	1.99	2.27	2.08	2.21	2.00	-	6.74
**IO**-Na^+^I^–^	172.0	171.1	3.60	3.54	2.32	2.44	2.39	2.43	2.31	-	7.03
**IO**-K^+^I^–^	170.6	169.5	3.61	3.55	2.67	2.72	2.73	2.74	2.65	-	7.35
**IO**-Cu^+^I^–^	171.8	170.8	3.62	3.55	2.09	2.34	2.13	2.40	2.17	-	6.78
**IO**-Ag^+^I^–^	172.0	169.5	3.60	3.57	2.37	2.45	2.47	2.61	2.43	-	6.95
**IO**-Au^+^I^–^	170.2	170.7	3.64	3.51	2.12	2.98	2.15	2.90	2.82	-	6.92
**IO**-Ni^2+^PF_6_^–^I^–^	171.2	165.5	3.69	3.52	1.92	1.96	1.90	1.96	2.55	5.25	6.62
**IO**-Zn^2+^PF_6_^–^I^–^	170.4	174.7	3.66	3.48	2.04	2.06	2.12	2.09	2.05	2.20	6.81
**IS**-Li^+^I^–^	170.2	174.6	3.62	3.50	2.50	2.65	2.60	2.82	2.56	-	7.20
**IS**-Na^+^I^–^	169.3	171.7	3.64	3.53	2.85	3.05	2.93	3.17	2.91	-	7.26
**IS**-K^+^I^–^	166.6	169.0	3.66	3.56	3.17	3.39	3.23	3.31	3.24	-	7.38
**IS**-Cu^+^I^–^	173.7	173.6	3.58	3.55	2.39	2.40	2.49	3.72	2.42	-	6.47
**IS**-Ag^+^I^–^	170.3	174.4	3.62	3.50	2.63	2.68	2.78	3.51	2.69	-	7.03
**IS**-Au^+^I^–^	170.3	174.4	3.62	3.49	2.53	2.51	3.04	3.79	2.75	-	6.89
**IS**-Ni^2+^PF_6_^–^I^–^	173.9	171.2	3.57	3.55	2.65	2.34	2.28	2.30	2.31	2.45	7.08
**IS**-Zn^2+^PF_6_^–^I^–^	171.1	174.1	3.61	3.49	2.59	2.59	2.67	2.58	2.56	2.21	7.16
**TeO**-Li^+^I^–^	168.4	171.5	3.72	3.64	1.99	2.33	2.05	2.22	1.99	-	6.69

a σ_1_ and σ_2_ refer to the σ–hole donor atom interacting with the anion, particularly I and Te.

The values reported in Table 1 serve as a diagnostic tool, indicating that the applied anions, in all structures, are favorably interacting with the σ – hole donor atoms given by the almost linear angles obtained in both C-σ_1_ ···X^–^ and C−σ_2_··· X^–^, ranging from 168.4 to 174.4°. Furthermore, the distances between the anions and the interacting σ – hole donor atoms, **d1** and **d2**, range from 3.10 to 3.69 Å, reveal that the halogen bonds formed are stabilized, since they are significantly smaller than the sum of the van der Waals or ionic radii, with a reduction ratio smaller than 1.0 which is further confirmed by the EDA-NOCV results for the anion interaction (Table 2).

**Table 2:** Energy Decomposition Analysis, EDA-NOCV (kcal.mol^‑1^) Involving the [2]­Catenane **IO** ^a^

						Hirshfeld Population Analysis	
	ΔE^tot^	ΔE^elstat^	ΔE^Pauli^	ΔE^oi^	ΔE^disp^	**IO**-M^+^ | M^2+^	X^–^
**IO**-Li^+^···Cl^–^	–101.9	–96.3	47.9	–46.8	–6.7	+0.46	–0.46^a^
		(64.3%)		(31.2%)	(4.5%)		
**IO**-Li^+^···Br^–^	–96.6	–94.0	47.9	–42.3	–8.2	+0.46	–0.46
		(65.1%)		(29.3%)	(5.7%)		
**IO**-Li^+^···I^–^	–91.4	–84.7	40.0	–36.8	–9.9	+0.57	–0.57
		(64.5%)		(28.0%)	(7.53%)		
**IO**-Na^+^···I^–^	–90.5	–83.9	40.5	–37.5	–9.6	+0.57	–0.57
		(64.0%)		(28.6%)	(7.3%)		
**IO**-K^+^···I^–^	–89.2	–82.33	40.1	–37.6	–9.4	+0.57	–0.57
		(63.7%)		(29.1%)	(7.3%)		
**IO**-Cu^+^··· I^–^	–91.7	–84.7	39.9	–36.9	–9.9	+0.57	–0.57
		(64.4%)		(28.1%)	(7.5%)		
**IO**-Ag^+^··· I^–^	–90.9	–83.7	39.3	–36.8	–9.7	+0.58	–0.58
		(64.3%)^a^		(28.3%)	(7.5%)		
**IO**-Au^+^··· I^–^	–93.6	–87.3	41.9	–38.3	–9.9	+0.57	–0.57
		(64.4%)		(28.3%)	(7.3%)		
**IO**-Ni^2+^PF_6_^–^···I^–^	–107.6	–100.9	43.1	–39.2	–10.7	+0.53	–0.53
		(66.9%)		(26.0%)	(7.1%)		
**IO**.Zn^2+^PF_6_^–^···I^–^	–101.2	–94.5	43.0	–39.7	–10.1	+0.56	–0.56
		(65.5%)		(27.5%)	(7.0%)		
						**IO**.X^–^	M^+^ | M^2+^
**IO**-I···Li^+^	–175.1	–127.0	23.8	–60.1	–11.8	–0.14	+0.14
		(63.9%)		(30.2%)	(5.9%)		
**IO**-I···Na^+^	–142.2	–118.6	31.7	–40.2	–15.2	–0.24	+0.24
		(68.2%)		(23.1%)	(8.7%)		
**IO**-I···K^+^	–123.4	–107.2	38.5	–35.8	–18.9	–0.27	+0.27
		(66.2%)		(22.1%)	(11.7%)		
**IO**-I^–^ ··· Cu^+^	–198.2	–190.0	101.5	–91.2	–18.4	+0.03	–0.03
		(63.4%)		(30.4%)	(6.1%)		
**IO**-I^–^ ··· Ag^+^	–168.3	–154.5	78.0	–69.0	–22.8	–0.22	+0.22
		(62.7%)		(28.0%)	(9.2%)		
**IO**-I^–^ ··· Au^+^	–192.6	–197.6	154.2	–125.8	–23.5	–0.22	+0.22
		(57.0%)		(36.3%)	(6.8%)		
**IO**-I^–^PF_6_^–^···Ni^2+^	–653.4	–445.9	199.9	–388.5	–19.0	–0.10	+0.10
		(52.2%)		(45.5%)	(2.2%)		
**IO**-I^–^PF_6_^–^···Zn^2+^	–623.7	–431.8	98.7	–271.9	–18.7	–0.41	+0.41
		(59.8%)		(37.6%)	(2.6%)		

a % of attractive int. (ΔE^elstat^ + ΔE^oi^ + ΔE^disp^).

b In the upper block of the table are the results related to the decomposition of anion interactions, for which the following fragmentation scheme was used: f1 = [2]­catenane + cation (+ counterion) and f2 = the interacting anion (**IO**-M^+^ ···X^–^ or **IO**- $M^{2+}{{\mathrm{PF}}_{6}}^{\text{−}}\cdot \cdot \cdot$ X^–^). In the lower block are the results of the cation decompositions, where the following fragmentation scheme was used: f1 = [2]­catenane + anion (+ counterion) and f2 = the interacting cation (**IO**-I^–^ ···M^+^ or **IO**- $I^{\text{−}}{{\mathrm{PF}}_{6}}^{\text{−}}\cdot \cdot \cdot$ M^2+^). The Hirsheld analysis for the interacting fragments is also reported.

This pattern has been identified in all structures (**IO**, **IS**, and **TeO**) when the applied anion is I^–^, without any significant geometric changes when applying different cations, also being corroborated by the EDA-NOCV results available in Tables 2 and 3. The halogen bonds involving the chloride and bromide anions are relatively shorter than those involving iodide, ranging from 3.10 to 3.31 Å, indicating that the anion interaction with the [2]­catenane is dependent on the size of the applied anion, and then Cl^–^, the smallest applied anion, exhibits the shorter distances in relation to the σ – hole interaction donor atoms, resulting in the most stabilizing interaction (ΔE^tot^ = −101.9 kcal.mol^‑1^ in **IO**-Li^+^··· Cl^–^), while **IO**-Li^+^···Br^–^ and **IO**-Li^+^···I^–^ exhibit total interaction energies of −96.6 and −91.4 kcal.mol^‑1^, respectively (see Table 2).

**Table 3:** Energy Decomposition Analysis, EDA-NOCV (kcal/mol) Involving the [2]­Catenanes **IS** and **TeO** ^a^

						Hirshfeld Population Analysis	
	ΔE^tot^	ΔE^elstat^	ΔE^pauli^	ΔE^oi^	ΔE^disp^	**IS**-M^+^ | M^2+^	X^–^
**IS**-Li^+^···I^–^	–90.0	–83.8	42.0	–38.1	–10.2	+0.57	–0.57
		(63.4%)^a^		(28.8%)	(7.7%)		
**IS**-Na^+^···I^–^	–90.0	–82.9	40.1	–37.0	–10.2	+0.58	–0.58
		(63.7%)		(28.4%)	(7.8%)		
**IS**-K^+^···I^–^	–89.4	–81.6	38.8	–36.4	–10.2	+0.58	–0.58
		(63.7%)		(28.4%)	(8.0%)		
**IS**-Cu^+^···I^–^	–91.5	–84.0	41.4	–38.5	–10.4	+0.57	–0.57
		(63.2%)		(29.0%)	(7.8%)		
**IS**-Ag^+^···I^–^	–90.6	–84.3	42.2	–38.3	–10.2	+0.57	–0.57
		(63.5%)		(28.8%)	(7.7%)		
**IS**-Au^+^···I^–^	–91.3	–85.2	42.9	–38.8	–10.2	+0.57	–0.57
		(63.5%)		(28.9%)	(7.6%)		
**IS**-Ni^2+^PF_6_^–^···I^–^	–98.4	–90.6	40.3	–38.5	–9.6	+0.58	–0.58
		(64.0%)		(28.7%)	(7.3%)		
**IS**-Zn^2+^PF_6_^–^···I^–^	–99.2	–92.6	45.5	–41.5	–10.6	+0.56	–0.56
		(64.0%)		(28.7%)	(7.3%)		
**TeO**-Li^+^···I^–^	–88.5	–82.3	15.4	–32.3	–11.8	+0.59	–0.59
		(65.1%)		(25.6%)	(9.3%)		
						**IS**-X^–^	M^+^ | M^2+^
**IS**-I^–^ ···Li^+^	–154.0	–98.2	20.3	–63.7	–12.4	–0.06	+0.06
		(56.3%)		(36.5%)	(7.1%)		
**IS**-I^–^ ···Na^+^	–127.6	–90.2	21.3	–42.3	–16.4	–0.19	+0.19
		(60.6%)		(28.4%)	(11.0%)		
**IS**-I^–^ ··K^+^	–111.5	–84.8	28.8	–36.6	–18.9	–0.23	+0.23
		(60.4%)		(26.1%)	(13.5%)		
**IS**-I^–^ ···Cu^+^	–226.4	–202.8	124.2	–127.6	–20.2	+0.16	–0.16
		(57.8%)		(36.4%)	(5.8%)		
**IS**-I^–^ ··· Ag^+^	–188.1	–167.8	99.4	–95.4	–24.3	–0.07	+0.07
		(58.4%)		(33.2%)	(8.5%)		
**IS**-I^–^ ··· Au^+^	–234.9	–212.7	154.1	–149.9	–26.5	–0.07	+0.07
		(54.7%)		(38.5%)	(6.8%)		
**TeO**-I^–^ ···Li^+^	–175.2	–126.9	23.8	–60.4	–11.8	–0.15	+0.15
		(63.7%)		(30.3%)	(6.0%)		
**IS**-I^–^PF_6_^–^···Ni^2+^	–702.5	–404.3	181.9	–461.5	–18.5	+0.16	–0.16
		(45.7%)		(52.2%)	(2.1%)		
**IS**-I^–^PF_6_^–^···Zn^2+^	–616.3	–354.1	64.0	–306.9	–19.3	–0.25	+0.25
		(52.1%)		(45.1%)	(2.8%)		

a % of attractive int. (ΔE^elstat^ + ΔE^oi^ + ΔE^disp^).

b In the upper block of the table are the results related to the decomposition of anion interactions, for which the following fragmentation scheme was used: f1 = [2]­catenane + cation (+ counterion) and f2 = the interacting anion (**IS**| **TeO**-M^+^··· X^–^ or **IS**. $M^{2+}{{\mathrm{PF}}_{6}}^{\text{−}}\cdot \cdot \cdot$ X^–^). In the lower block are the results of the cation decompositions, where the following fragmentation scheme was used: f1 = [2]­catenane + anion (+ counterion) and f2 = the interacting cation (**IS**| **TeO**-I^–^ ···M^+^ or **IS**- $I^{\text{−}}{{\mathrm{PF}}_{6}}^{\text{−}}\cdot \cdot \cdot$ M^2+^). The Hirsheld analysis for the interacting fragments is also reported.

According to Table 1, the distances **d3** to **d7** depict how cations interact with the crown ether portion of the [2]­catenane. The distances between the applied cations and the interacting site atoms range from 1.92 to 3.79 Å, these being mostly smaller than the distances established between the anions and the σ-hole donor atoms, **d1** and **d2**, indicating that the cations must be better stabilized by this crown ether portion than the anions by the halogen bonds. This trend is strongly corroborated by the values obtained in the energy decomposition analysis, EDA-NOCV.

The EDA-NOCV results provide further confirmation of these indicatives. Notice that the distances undergo significant changes when the interacting cation presents an increased ionic radius. Among the alkali metals, Li^+^ has the shortest distances with several neighboring atoms, with distances ranging from 1.98 to 2.82 Å between the structures **IO**, **IS**, and **TeO**, indicating that the interaction with Li^+^ will present the most favorable values, being followed by Na^+^ and K^+^ with distance values ranging from 2.31 to 3.17 Å and 2.65 to 3.39 Å, respectively. These findings reveal that the recognition of cations by the employed [2]­catenanes presents a behavior similar to that observed in the recognition of anions, in which the smaller interacting ion establishes smaller distances in relation to the atoms of the interaction site and establishes more stabilizing interactions, as confirmed by the following trend $E_{{\mathrm{Li}}^{+}}^{tot}$ < $E_{Na+}^{tot}$ < $E_{K+}^{tot}$ (Tables 2 and 3).

The monovalent, M^+^, coinage metal ions (Cu^+^, Ag^+^, and Au^+^) exhibit a tendency similar to that of alkali metal ions Li^+^, Na^+^, and K^+^ in terms of the interaction distances they establish with neighboring atoms (distances **d3** - **d7**) and how these change depending on the ionic radii. As the latter increases, these distances also increase (Table 1).

For instance, Cu^+^ exhibits the shortest values of **d3**-**d7**, while Ag^+^ presents intermediate values ranging from 2.37 to 3.51 Å and Au^+^ presents the longest distances ranging from 2.12 to 3.79 Å (Table 1). Concerning cation interaction with **IO**, the results indicate that Cu^+^ will exhibit the strongest interaction when compared with Ag^+^ and Au^+^, given that Cu^+^ presents smaller ionic radii and larger coordination number (CN). Notice that Ag^+^ and Au^+^, when compared to each other, exhibit interesting distance values. For example, **d3** and **d5** present the smallest values, in the case of Au^+^, diverging slightly from the distances obtained with Cu^+^. In contrast, the **d4**, **d6**, and **d7** values are smaller for the Ag^+^ ion than those for Au^+^. The results clearly indicate that both Au^+^ and Ag^+^, when interacting with [2]­catenanes, exhibit a reduced CN compared to Cu^+^ ( ${\mathrm{CN}}_{{\mathrm{Au}}^{+}}\approx$ 2, ${\mathrm{CN}}_{{\mathrm{Ag}}^{+}}\approx$ 4, and ${\mathrm{CN}}_{{\mathrm{Cu}}^{+}}\approx$ 5).

Regarding the modified [2]­catenate **IS**, which contains a thiacrown ether moiety, when interacting with transition metal cations, the **d3**-**d7** distance values indicate that the interaction of all cations will undergo a greater repulsive component with sulfur atoms. This is due to the sizes of the interaction site atoms and the applied cations. However, there is greater distortion of the molecule when interacting with Au^+^, which is due to the large overall **d3**-**d7** distances, which range from 2.51 to 3.79 Å. In contrast, Ag^+^, which ranges from 2.63 to 3.51 Å.

For M^2+^, Ni^2+^, and Zn^2+^ cations, the interaction with **IO** and **IS** presented smaller distances compared to all other cations, averaging 2.06 and 2.07 Å in **IO**, and 2.38 and 2.60 Å in **IS** for Ni^2+^ and Zn^2+^, respectively. These results indicate that both Ni^2+^ and Zn^2+^ will present the highest interaction values with catenanes **IO** and **IS**, with the Ni^2+^ interaction being even more favorable than that of Zn^2+^. Furthermore, the presence of the counterion, ${{\mathrm{PF}}_{6}}^{\text{−}}$ , causes a significant variation in the **dCI** distance when comparing Ni^2+^ interacting with **IO** (5.25 Å) and with **IS** (2.45 Å), indicating a direct coordination of the counterion, ${{\mathrm{PF}}_{6}}^{\text{−}}$ , with Ni^2+^. On the other hand, the **dCI** value remains practically unchanged when the interacting ion is Zn^2+^. The obtained dC···A values range from 6.44 Å to 7.38 Å, indicating that the employed heteroditopic [2]­catenanes are separated ion-pair receptors, in which the bound ions are spatially distant from each other, with the receptor architecture physically separating them. (See all optimized structures of **IO**- $M^{2+}{{\mathrm{PF}}_{6}}^{\text{−}}$ and **IS**- $M^{2+}{{\mathrm{PF}}_{6}}^{\text{−}}$ in Figure S3a-d.)

Finally, when comparing the geometric parameters between the systems **IO**-Li^+^I^–^, **IS**-Li^+^I^–^, and **TeO**-Li^+^I^–^ we have an overview of the modifications in both the anion- and cation-recognizing portions. This comparative analysis makes it clear that when going from **IO**-Li^+^I^–^ to **IS**-Li^+^I^–^, since the anion-recognizing portion is not modified, the parameters do not change, whereas the distances between the atoms of the thia-crown ether moiety increase significantly. On the other hand, when comparing **IO**-Li^+^I^–^ and **TeO**-Li^+^I^–^, we notice that the halogen bond donor moiety changes, exhibiting an increase in the **d1** and **d2** distances, since the σ-hole donor I is replaced by – Te – CH_3_. These results suggest that both moieties can be separately redesigned when searching for more selective interactions of certain cations and anions.

### 3.2 Bonding Situations

The results of the EDA-NOCV analysis of the interaction between [2]­catenane and the applied ions indicate that the structure exhibits a more stable interaction with the applied cations than with the interacting anions (Tables 2 and 3). Concerning the interactions of anions with the [2]­catenane **IO** (Table 2), the results reveal that both Cl^–^ and Br^–^ exhibit more favorable interactions with **IO** than I^–^, with ΔE^tot^ values being −101.9, −96.6 and 91.4 kcal.mol^–1^, respectively. The results of **IO**-Li^+^··· X^–^, (with X = Cl, Br), presents ΔE^elstat^ and ΔE^oi^ as the most significant contributors, ranging from 64.3% to 65.1% and 31.2% to 29.3% of the total attractive interaction, respectively. In the case of **IO**-M^+^··· I^–^ (where M = Li, Na, K, Cu, Ag, Au), the results demonstrate that the total interaction energies, ΔE^tot^, exhibit a high degree of similarity in terms of stabilization, independent of the applied cation. For instance, ΔE^tot^ ranges from −89.2 to −93.6 kcal.mol^–1^. Similarly, the electrostatic, ΔE^elstat^, and orbital, ΔE^oi^, components are the primary stabilizing factors. The electrostatic contribution has been found to be responsible for 64–67% of the stabilization, while the orbital contribution contributes approximately 28%. Among the stabilizing contributions in systems **IO**-M^+^··· X^–^ (with X = Cl, Br, I), the dispersive one, ΔE^disp^, accounts for the smallest contribution, ranging from 4.5% to 7.5% of the total stabilizing interaction. In the case of **IO**- $M^{2+}{{\mathrm{PF}}_{6}}^{\text{−}}\cdot \cdot \cdot I^{\text{−}}$ (where M = Ni, Zn), the value of ΔE^tot^ is slightly more favorable than compared to all **IO**-M^+^··· I^–^ cases, being −107.6 and −101.2 kcal.mol^–1^, respectively.

With regard to the cation interactions, as **IO**-I^–^ ··· M^+^, the aggregate interaction, undergoes a substantial enhancement, ranging from −123.4 to −198.2 kcal.mol^–1^, the overall stability of the interactions still remains dependent on the components, ΔE^elstat^, ΔE^oi^, and ΔE^disp^, which presented a significant increment when compared with the corresponding anion interactions, **IO**-M^+^··· I^–^. For example, comparing the values of such components for series **IO**-M^+^··· I^–^ and **IO**-I^–^ ··· M^+^ (where M = Li, Na, K, Cu, Ag, Au), it is noted that they become more stabilizing for cation interactions. The results concerning the **IO-**I^–^ ··· M^+^ interactions reveal that one of the key factors for the cation recognition in **IO** is their size, suggesting that smaller cations interact strongly with the crown ether portion. However, some exceptions to this trend can be observed, as in the cases of silver­(I) and gold­(I) ions. As can be observed Au^+^ has a more favorable ΔE^tot^ than Ag^+^, which stems from the ion coordination behaviors; while silver­(I) exhibits a coordination number (CN) of 4, gold­(I) exhibits CN = 2 to the crown ether portion of **IO** (as indicated by the results in Table 1), which are also in line with the values by Liao et.^52^ It can be concluded that R­(Au^+^) < R­(Ag^+^) (where ${\mathrm{CN}}_{{\mathrm{Au}}^{+}}$ = 2 → 70 pm; ${\mathrm{CN}}_{{\mathrm{Ag}}^{+}}$ = 4 → 114 ∼ 116 pm). As expected, the cation interactions in **IO**- ${{\mathrm{PF}}_{6}}^{\text{−}}\cdot \cdot \cdot M^{2+}$ (where M = Ni, Zn) are much more stabilizing than the anion interactions in **IO**- $M^{2+}{{\mathrm{PF}}_{6}}^{\text{−}}\cdot \cdot \cdot I^{\text{−}}$ .

The Hirshfeld population analysis corroborates the trends obtained for the electrostatic contributions. The data demonstrate that electron density invariably migrates from the anion or anion-containing fragment to the cation or cation-containing fragment. In the context of anion interactions, an observation was made of the flow of electronic density. This flow occurs from the applied anion, X^–^, toward the [2]­catenane that contains or interacts with the cation, M^+^. This interaction leads to a depletion of the cation net charge from +1­(e) to +0.57­(e) when interacting with I^–^. Conversely, the iodide displays a reduction in negative charge, amounting to −0.57­(e). It is evident that the resulting values of charge are consistent for **IO**-M^+^··· I^–^ irrespective of the applied M^+^. A similar pattern is exhibited when the interaction occurs with chloride (Cl^–^) and bromide (Br^–^). However, the higher degree of interaction exhibited by both anions with **IO**-M^+^ results in a greater decrease in the negative charge, amounting to −0.46­(e). The findings reported herein are consistent with the recognized characteristics of the applied cation, suggesting that the ion’s effect on the anion interaction remains negligible. This conclusion is further substantiated by the population analysis results. It has been proposed that anion recognition may not be as favorable as cation recognition. This phenomenon can be attributed to the predominant interaction of the anions with the σ-hole bond donor atoms, while the cations are positioned in a more favorable coordination environment.

For cation recognition, the Hirshfeld Population Analysis results reveal that M^+^ has a higher net charge redistribution with [2]­catenane, which complements the obtained results from the ΔE^tot^ term. For all three applied alkali metal cations were originally +1­(e) when isolated and become +0.14­(e), +0.24­(e), and +0.27­(e), following across Li^+^, Na^+^, and K^+^, respectively. In the context of the coinage-metal cations, Au^+^ and Ag^+^ were originally +1­(e) when isolated and become +0.22­(e) when interacting with **IO**, and **IO**-X^–^ net charge changes from −1­(e) to −0.22­(e), while Cu^+^ exhibited the most pronounced net charge distribution. The initial charge of +1­(e) diminished to −0.03­(e) upon interacting with **IO**.X^–^, while **IO**.X^–^ initially possessing a net charge of −1­(e) changes to +0.03­(e). This outcome aligns with the EDA-NOCV results, which demonstrate that Cu^+^, among all applied M^+^ cations, exhibits the most favorable interaction with **IO**-I^–^. This phenomenon is ascribed to the optimal coordination site, which facilitates interaction through coordination with oxygen atoms of the crown ether moiety.

When **IO** interacts with M^2+^ ions, a higher amount of charge inflow and outflow during the ion recognition process is observed. The results of this study align with the findings of the EDA-NOCV analysis, which indicated that Ni^2+^ exhibited a more favorable interaction than Zn^2+^, resulting in the accumulation of the most negative net charge from **IO**-I^–^, −0.41­(e).

The EDA-NOCV results in Table 3 provide further insights regarding how the magnitude of interactions with ions changes as a function of modifications in the structure of [2]­catenane, **IS**, and **TeO**. Relative to **IS**, the crown ether portion of **IO** is modified by replacing the oxygen atoms with sulfur atoms, yielding a crown thioether moiety. Relative to **TeO**, both σ – hole-donating iodines are replaced by – Te – CH_3_ groups. According to the EDA-NOCV results, the anion recognition does not present significant changes (Tables 2 and 3) either with the replacement of oxygen atoms by sulfur atoms or with the replacement of σ – hole donor atoms, since the anion is kept bound to the catenane exclusively through σ-hole interactions. In contrast, the cation interaction exhibits more significant changes, since both M^+^ and M^2+^ are better stabilized in the crown ether/thioether portion since it has a greater number of interacting atoms.

Depending on the nature of the cation, for example, alkali metal (Li^+^, Na^+^, K^+^) or coinage (Cu^+^, Ag^+^, Au^+^) cations, they may present gain or loss of stabilization with the modification of the portion that interacts with them, such as going from **IO** to **IS** (Tables 2 and 3).

For instance, in the case of **IS**.I^–^ ···M^+^, where M = Li, Na, K, a significant decrease in the interaction strength is observed, resulting from the change in the crown ether to crown thioether moiety. However, an interaction pattern analogous to that observed in **IO** is repeated in **IS**, where $\Delta E_{{\mathrm{Li}}^{+}}^{tot}$ < $\Delta E_{{\mathrm{Na}}^{+}}^{tot}$ < $\Delta E_{K^{+}}^{tot}$ . This behavior, where a harder cation like Li^+^ exhibits a more stabilizing interaction with a given cavity of the molecule than Na^+^ and K^+^, even in a softer environment, is directly related with the size match between the ionic radius of the cation and the cavity size of the crown ether portion, along with the charge density of the ion. It is well-established that lithium ions have a smaller ionic radius (approximately 0.136 nm) and a significantly higher charge density than potassium ions (approximately 0.256 nm). On the other hand Crown ethers/thioethers are cyclic molecules containing a cavity lined with oxygen or sulfur atoms that can form ion-dipole interactions with cations. The stability of the resulting interaction is maximized when the cavity size closely matches the ionic radius of the guest cation, a principle known as the *size match rule*. For instance, 12-crown-4, with a cavity size of about 0.12–0.15 nm, binds Li^+^ much more strongly than K^+^. Conversely, a 18-crown-6, with a larger cavity (0.26–0.32 nm), exhibits a higher affinity for K^+^ than for Li^+^.^53^ Therefore, the difference in binding affinity is attributed to the high differences in charge density between the alkali metal ions, which directly influence the strength of the electrostatic interactions with the electron-rich atoms in the crown ether portion. Therefore, in the present case, the crown ether/thioether portions in [2]­catenanes **IO and IS** exhibit a cavity size that fits better with smaller alkali cations.

On the other hand, when coinage metal cations are employed, on going from **IO**.I^–^ ···M^+^ to **IS**.I^–^ ···M^+^ (M = Cu, Ag, Au), the total interaction energy values, ΔE^tot^, becomes much more stable, changing from −198.2, −168.3, and −192.6 to −226.4, −188.1, and −234.9 kcal.mol^–1^ for Cu^+^, Ag^+^, Au^+^, respectively. The coinage metal ions interact more strongly with crown thioethers than with crown ethers due to the significant influence of the sulfur donor atoms in the thioether portion. These soft metal ions have a high affinity for soft donor atoms such as sulfur, which are more effective at coordinating with them compared to the harder oxygen donor atoms found in standard crown ethers. Such a claim is confirmed by the EDA-NOCV analysis, which confirms that not only the electrostatic contribution but also the orbital and dispersion terms become more stabilizing when such cations interact with the thioether portion of the [2]­catenane. The presence of thioether moiety enhances the stability of the resulting metal interaction, particularly for soft cations such as Ag^+^ and Au^+^. That is not an isolated case, since studies have shown that polymers modified with mixed-donor crown ethers containing thioether groups exhibit very high selectivity and capacity for Ag^+^ ions.^53^

The interaction with M^2+^ cations presented the most stabilizing values of interaction energy, with both **IO** and **IS** [2]­catenanes, where in both cases Ni^2+^ exhibited the most favorable energy values of ΔE^tot^. Notice that for **IO**, $\Delta E_{{\mathrm{Ni}}^{2+}}^{tot}$ and $\Delta E_{{\mathrm{Zn}}^{2+}}^{tot}$ present a slight difference of around 29.7 kcal.mol^–1^, demonstrating that both electrostatic and orbital interaction terms are the predominant contributors to the overall attractive interaction of the system (with %ΔE^elstat^ > %ΔE^oi^). However, when interacting with **IS**, the ΔE value in between $\Delta E_{{\mathrm{Ni}}^{2+}}^{tot}$ and $\Delta E_{{\mathrm{Zn}}^{2+}}^{tot}$ significantly increases, being of around 86.2 kcal.mol^–1^, both electrostatic and orbital interaction terms remain as the main contributors but now with %ΔE^elstat^ < %ΔE^oi^ for Ni^2+^, while Zn^2+^ presents the same pattern as exhibited in **IO**. When the EDA-NOCV results are rationalized in conjunction with the geometric parameters, it can be deduced that Ni^2+^, when interacting with **IO**, adopts a square planar geometry. Conversely, in **IS**, it assumes an octahedral geometry. This preference can be attributed to the increased repulsion caused by the more substantial sulfur atoms, leading to a structural distortion in **IS** (RMSD_**IO**→**IS**_: 1.68) and the position with the counterion, ${{\mathrm{PF}}_{6}}^{\text{−}}$ , now directly coordinated with Ni^2+^. This change in geometry assumed by Ni^2+^ results in an increase of its ionic radii, going from 63 pm in a square planar geometry to 83 pm in an octahedral environment. It contrasts with the results exhibited by Zn^2+^ when interacting with both **IO** and **IS**. In this particular instance, the cation is situated within an octahedral coordination environment, presenting an ionic radius of 88 pm without any variations or significant structural distortions caused by the replacement of oxygen atoms with sulfur (RMSD_**IO**→**IS**_ = 0.86). When depicting the distances in Table 1 (**d3** - **d7**) it can be seen that for both employed cations M^2+^ an increase in distances with the change of the interactive environment (**IO**- $M^{2+}{{\mathrm{PF}}_{6}}^{\text{−}}I^{\text{−}}$ → **IS**- $M^{2+}{{\mathrm{PF}}_{6}}^{\text{−}}I^{\text{−}}$ ) is observed. However, Ni^2+^ always presents shorter distances than Zn^2+^. Alongside all info provided by both EDA-NOCV and geometric parameters, the electronic configuration of both M^2+^ must be taken into consideration. While Ni^2+^ presents a d^8^ electronic configuration, Zn^2+^ exhibits a complete filled orbital being d^10^, resulting in a more favorable interaction for Ni^2+^ than Zn^2+^ with both [2]­catenanes **IO** and **IS**.

The Hirshfeld population analysis for both anion and cation interactions, while both [2]­catenanes interact with a M^2+^ ion, demonstrates the same exhibited behavior for M^+^ where a more significant net charge flow is observed in the cation interaction. In the context of anion recognition, I^–^, in both structures, was originally −1­(e) when isolated, and presented similar amounts of charge flow when interacting with **IO**- $M^{2+}{{\mathrm{PF}}_{6}}^{\text{−}}$ and **IS**- $M^{2+}{{\mathrm{PF}}_{6}}^{\text{−}}$ (M = Ni and Zn), ranging from −0.53­(e) to −0.58­(e). In the case of the cation recognition of M^2+^ interacting with **IO**- $I^{\text{−}}{{\mathrm{PF}}_{6}}^{\text{−}}$ and **IS**- $I^{\text{−}}{{\mathrm{PF}}_{6}}^{\text{−}}$ , the initial charge of +1­(e) when isolated, for Ni^2+^ and Zn^2+^, is significantly diminished ranging from −0.16­(e) and +0.41­(e).

The NOCV analysis of all complexes, as depicted in Figures 3 and S4–S7 (Supporting Information material), corroborates with the obtained results from both energy decomposition and Hirshfeld population analyses. The sum of the energy contributions associated with all of the NOCV density deformation channels precisely equals the total orbital interaction energy, ΔE^oi^, in EDA-NOCV (Tables 2 and 3). It happens because each NOCV pair (Ψ_*k*_, Ψ_–*k*_) corresponds to a specific electron density deformation channel, Δρ_k_, that describes a particular type of interaction. Each of these deformation density channels has a corresponding energy contribution (ΔE_orb,k_) to the total orbital interaction energy.

**Figure 3:**
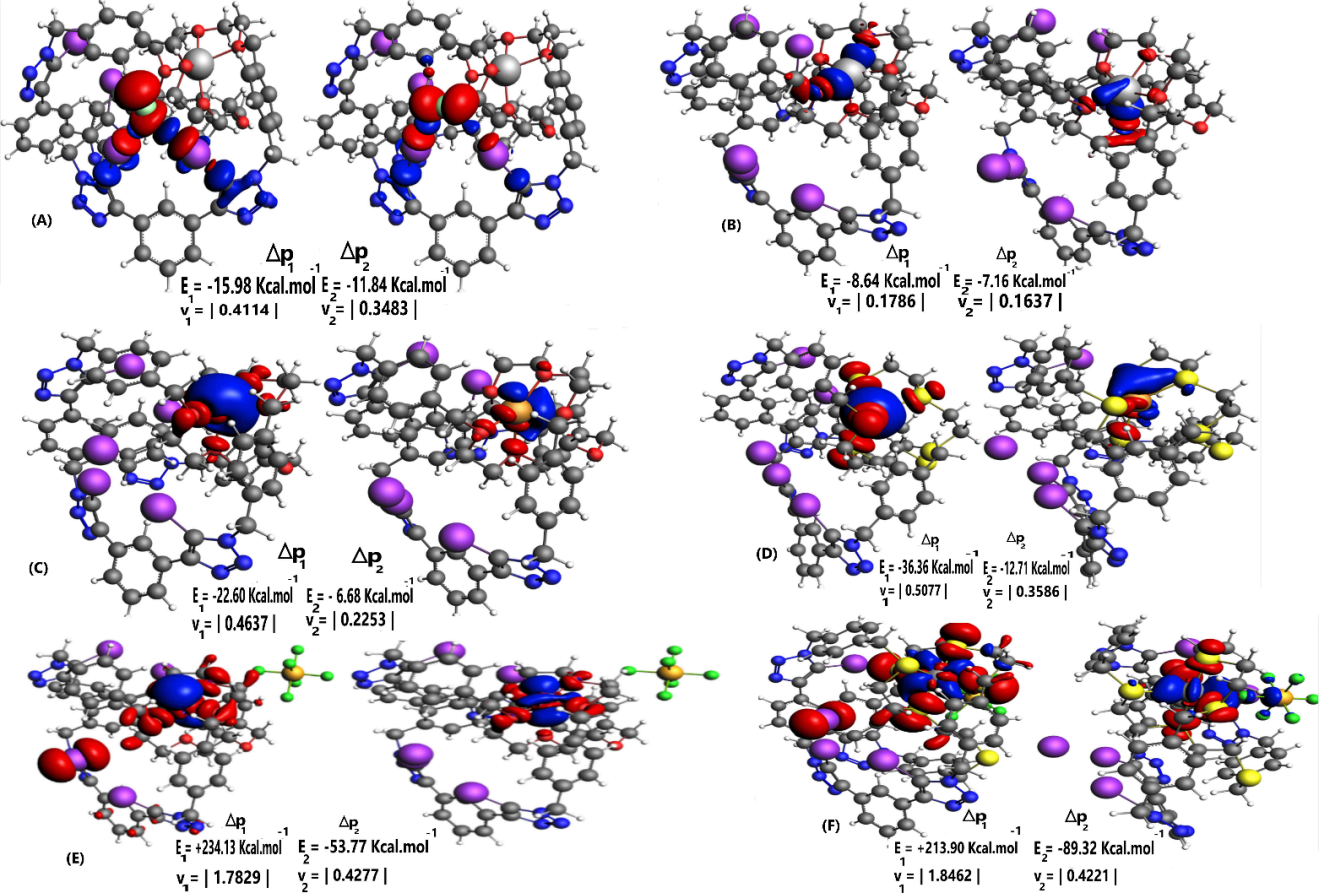
First two density deformation channel surface plots, ρ_1,2_ with isovalue: 0.001 au. In the following systems, the red and blue regions, respectively, indicate the outflow and inflow electron density: **(A) IO-Li** ^**+**^···Cl^–^; **(B) IO-I** ^**–**^ ···Li^+^; (C) **IO-I** ^**–**^ **···Cu** ^**+**^; **(D) IS-I** ^**–**^ **···Cu** ^**+**^; **(E) IO-I** ^**–**^ **···Ni** ^**2+**^; **(F) IS-I** ^**–**^ **···Ni** ^**2+**^.

In the examined systems, solely the initial two channels have been considered, Δρ_1_ and Δρ_2_, since they are the most significant for the studied interactions. For anion recognition, **IO**| **IS**| **TeO**-M^+^··· X^–^ shows that the first two density channels (Δρ_1_ and Δρ_2_) are related to the σ – hole interaction (C–I···X^–^) without any involvement of the cation binding pocket. For **IO**| **IS**-M^2+^··· X^–^ this behavior remains the same except when M^2+^ = Ni^2+^. A minor contribution from the cation and its binding pocket is evident, arising not only from the magnitude of its interaction as a result of its +2 charge but also from its coordination environment. In the interaction with **IO**, the less favorable square planar geometry facilitates the outflow of charge density from the cation and the oxygen surrounding it, in conjunction with the applied anion, to C – I, thereby promoting anion interaction. In the interaction with **IS**, a similar slight contribution from both Ni^2+^ and its binding pocket can be identified. However, the octahedron facilitates charge density inflow, which results in a slight decrease in anion recognition. In cation recognition, **IO**| **IS**| **TeO**-I^–^ ··· M^+^ exhibits Δρ_1_ and Δρ_2_ related with the M^+^ cation and its binding pocket atom, oxygen or sulfur. The density channels exhibit the charge inflow directly or closely to the cation, while the charge outflow around its interactive environment. It is seen that the coinage metal cations present higher charge inflow than the alkali, corroborating with the ΔE^tot^. **IO| IS**-I^–^ ···M^2+^, when M^2+^ = Zn^2+^, exhibits Δρ_1_ and Δρ_2_ related to the depletion of charge density from its surrounding atoms and accumulation in the cation. The density charge flow presents overall the same behavior given the octahedral geometry when interacting with both **IO** and **IS**. However, for M^2+^ = Ni^2+^, a destabilizing density charge flow is obtained in **IO** and **IS**, Δρ_1_, given to the cation’s elevated charge resulting in interfragment polarization and increasing the electrostatic component, alongside the Ni^2+^ interaction with both binding pockets leads to an increased electronic distortion effect, which corroborates the change in geometry determined from the geometric parameters and energy decomposition analysis. The geometry changes from square planar in **IO** to octahedral in **IS**. Δρ_2_, although less probable, presents a stabilized density charge flow related to the charge outflow from the surrounding atoms and an inflow to the cation. Notice that ${\mathrm{IS}}_{\Delta \rho_{2}}$ exhibits more stabilization than ${\mathrm{IO}}_{\Delta \rho_{2}}$ given to the present inflow contribution from ${{\mathrm{PF}}_{6}}^{\text{−}}$ in **IS** resulted from the variation of the cation’s assumed geometry.

The EDA-NOCV, in conjunction with the geometric parameters, helps to elucidate that in fact both size of the cation, influenced by the CN, and cavity size of the crown ether/thioether portions are main key factors modulating the cation recognition.

### 3.3 Alchemical Free-Energy Principles

Alchemical free energy principles in conjunction with homodesmotic reactions have been employed to calculate the free energy differences associated with both cation and anion interaction processes, as represented in Figure 4. ΔE_1_ provides the energy involved in the cation recognition, while ΔE_2_ in contrast into the anion recognition. ΔE_3_ and ΔE_4_ provide values that represent the energy required to separate both ions from the [2]­catenanes and to fragment all interacting constituent moieties of the overall structure, respectively. These two components can be employed to demonstrate the influence of the mechanical bond in both anion and cation recognition.

**Figure 4:**
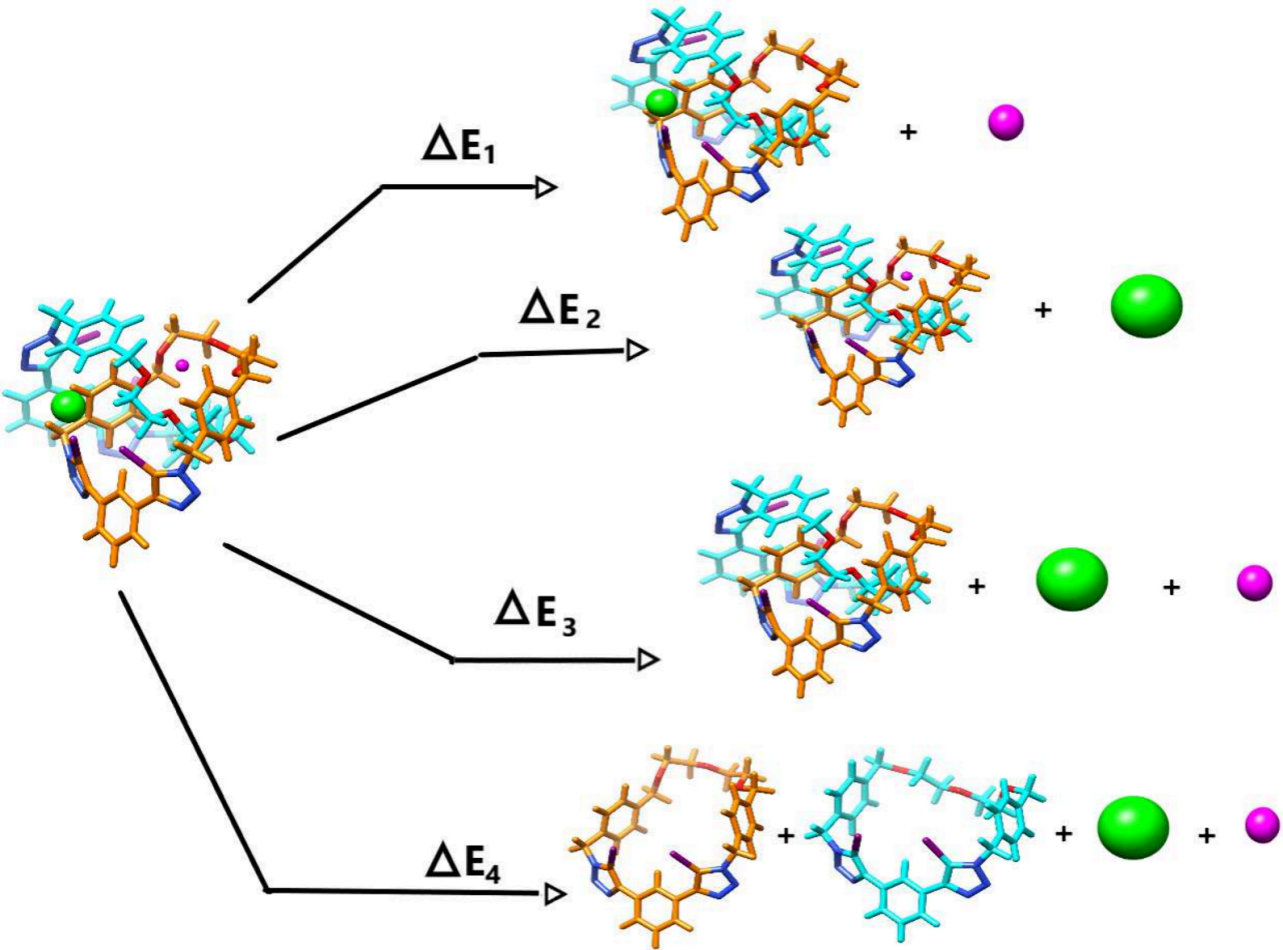
Homodesmostic reaction scheme to evaluate the overall stabilization when cations and anions interact with [2]­catenanes. (ΔE_1_) denotes de energy involved in the cation recognition, the (ΔE_2_) in the anion recognition, (ΔE_3_) the energy required to separate all applied ions interacting with the MIM, and the (ΔE_4_) is the energy separate all 4 components of the studied systems. The cations and anions are schematically represented by pink and green spheres, respectively.

Subtracting the ΔE_n_ values allows for a more in-depth understanding of the interplay between the various contributions involved in cation and anion recognition in the heteroditopic [2]­catenanes studied. Such an analysis was considered only for systems presenting the most significant interactions: **IO**-Li^+^I^–^, **IO**-Cu^+^I^–^, **IS**-Li^+^I^–^, and **IS**-Cu^+^I^–^. This allows us to shed new light on how favorable the recognition processes are, as well as the energetic costs of ion exchange and the existence of cooperativity. Finally, we can examine the role of the mechanical bonds of [2]­catenanes in the process.

As indicative from the results depicted in Table 4 and Figure 5, It is evident that all homodesmotic reactions are endothermic. This means that an energy input is required to remove the ions from the stabilizing environment provided by the [2]­catenanes and to separate the mechanical bond. The values also reveal that it takes much less energy to remove anions from the [2]­catenane than it does to remove cations because the latter are more strongly stabilized by the crown ether or thioether moieties of the [2]­catenanes.

**Figure 5:**
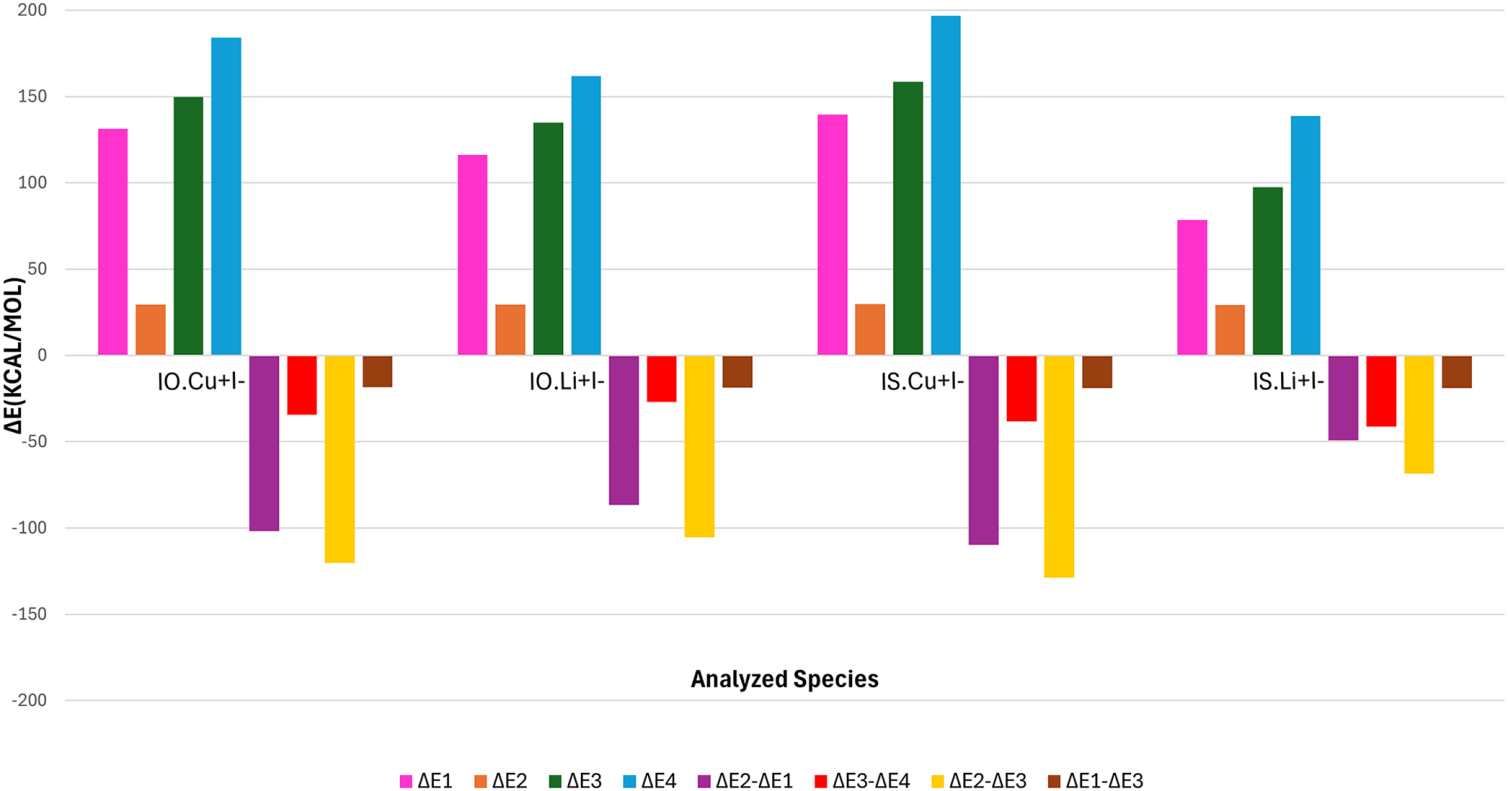
Contributions (kcal.mol^–1^) of the cation and anion interaction on the applied cations (Cu^+^ and Li^+^) as they interact with the [2]­catenanes **IO**–**IS** containing I^–^ following the scheme presented in Figure 4.

**Table 4:** Energy Values (ΔE_1_ – ΔE_4_, kcal.mol^‑1^) Concerning the Homosdesmotic Reactions Depicted in Figure 4

	ΔE_1_	ΔE_2_	ΔE_3_	ΔE_4_	(ΔE_2_ – ΔE_1_)	(ΔE_3_ – ΔE_4_)	(ΔE_2_ – ΔE_3_)	(ΔE_1_ – ΔE_3_)
**IO**-Cu^+^I^–^	132.3	29.5	149.8	184.2	–101.8	–34.4	–120.3	–18.5
**IO**-Li^+^I^–^	116.2	29.5	134.8	161.8	–86.7	–27.0	–105.3	–18.6
**IS**-Cu^+^I^–^	139.6	29.8	158.5	196.7	–109.8	–38.3	–128.7	–18.9
**IS**-Li^+^I^–^	78.5	29.2	97.6	138.8	–49.4	–41.3	–68.4	–19.1

In it, the most prominent values are in the interaction with the Cu^+^ cation in both **IO** and **IS** structures, with ΔE_1_ values of 132.3 and 139.6 kcal.mol^–1^, respectively. The values of ΔE_1_ for the Li^+^ cation reveal a decrease in the necessary energy when comparing the results from **IO** to **IS**, in line with EDA-NOCV results from Tables 2 and 3. This is because the interacting environment changes from harder to softer atoms interacting with a hard acid such as Li^+^. There is an energy difference of 37.7 kcal.mol^‑1^ when going from **IO** to **IS**. This is in contrast to what is observed with the Cu^+^ interaction. Since Cu^+^ is a soft acid, it results in a more stabilized interaction with **IS**. This makes it necessary to use a higher energy to withdraw the cation from the crown thiothermal moiety in **IS**. When analyzing the depicted results from ΔE_2_ all interactions presented in average the same value, of around 29.5 kcal.mol^–1^. This behavior is expected since the same anion, I^–^, was applied for all fragmentation schemes. Nonetheless, it is noticeable that a major difference in the necessary energy to perform ΔE_2_ in comparison with ΔE_1_, being observed by ΔE_2_ – ΔE_1_.

The subtraction of the ΔE_n_ values was plotted in Figure 5. For instance, ΔE_2_ – ΔE_1_ values demonstrate the enhanced stability achieved when performing the exchange of ions (exchanging the previously purely anionic interaction for a purely cation interaction). Such stabilization is much more significant for Cu^+^ than for Li^+^, given the significantly more favorable interaction values for Cu^+^ compared to Li^+^ in both catenanes (Table 2 and Table 3). ΔE_2_ – ΔE_3_ illustrates the stability gained when all of the interacting species are separated from each other and when the cation interacts with its respective binding site in the catenane. This relation can be contrasted with ΔE_1_ – ΔE_3_, which shows the stability gained when all components are separated, and only the anion interacts with the catenane at its respective binding site. As it can be observed, ΔE_2_ – E_3_ presents more stabilizing values than ΔE_1_ – E_3_ since the cation interaction, in accordance to the provided results of ΔE_1_ and in Tables 2 and 3, is higher in both catenanes than anion interactions. Cu^+^, as expected, presents the most stabilizing values, varying from −128.7 to −120.3 kcal.mol^–1^ when interacting with structures **IS** and **IO**, respectively. It becomes evident by the obtained values that **IS**.Li^+^presents the least stabilization gained by purely the addition of the cation, since it is with respect of a harder cation interacting with a softer environment composed of sulfur atoms. In all cases, ΔE_1_ – E_3_ is equivalent to the overall stability gained, given that all interactions involve the same applied anion (I^–^), resulting in stabilization values ranging from −18.5 to −19.1 kcal.mol^–1^. The role of the mechanical bond in ensuring the overall stability of the system is finally determined by ΔE_3_ – ΔE_4_ values, which quantify the stability provided by the mechanical bond, which varies from −27.0 to −41.3 kcal.mol^–1^. The [2]­catenane **IS** exhibits stronger mechanical bonds than **IO**. For **IS**.Li^+^ this becomes more stabilizing, when compared to **IO**.Li^+^, since it tends to compensate the less favorable interaction of **IS** with Li^+^, in which, the presence of Li^+^ does not result in the necessity of **IS** perform significant conformational changes in order to bind the cation (being a structure with a higher degree of preorganization). For **IS**.Cu^+^ the increase, when compared to its **IO** analogue, is expected since the overall interaction presents a higher degree of complementarity, presenting mutual electronic and spacial complementary binding sites to form the interaction.

## Concluding Remarks

The present study elucidates the anion and cation recognition process performed by the studied heteroditopic [2]­catenane **IO** from a computational electronic structure perspective. Cations such as Li^+^, Na^+^, K^+^, Cu^+^, Ag^+^, Ni^2+^, and Zn^2+^ have been considered in conjunction with the following anions chloride, bromide, and iodide. The role of different interactive environments for both anion and cation recognitions were also considered. The structure **IO** was modified by incorporating softer atoms in the crown ether moiety, by replacing the oxygen atoms for sulfur atom leading to the modified [2]­catenane structure **IS**. **IO** was further modified by incorporating a different σ – hole donor group, namely, −Te −CH_3_ groups as a chalcogen bond donor, leading to the [2]­catenane **TeO**. Insights into both cation and anion recognitions were gained by quantifying the contributions of not only the mechanical but also cation and anion contributions to the overall stability of the studied structures by using the EDA-NOCV energy decomposition, isodesmic reactions, and alchemical free-Energy principles.

The minimum structures and their geometric parameters demonstrate that all applied anions are stabilized in the specific heteroditopic [2]­catenane by purely σ – hole interactions. Furthermore, these parameters demonstrate a dependence on the size of the interacting anion, where Cl^–^ presented the most favorable interaction exhibiting deeper penetration in the [2]­catenane’s binding site. This behavior follows an inversely proportional order as the total interaction, ΔE^tot^, between the anion and the [2]­catenane decreases as the ionic radius of the applied anion increases. The presence of −Te −CH_3_ groups in **TeO** results in a reduction in interaction with the applied anion. This is due to the reduced σ-hole donor strength of the −Te −CH_3_ groups in comparison to iodine.

In the case of the cation recognition, the minimum structures and their geometric parameters demonstrate that all applied cations will obtain different coordination when interacting with the crown ether or thioether portions of **IO**. Additionally, such parameters are dependent on the minimum conformation adopted by the [2]­catenane and the nature of the interacting cation. Li^+^ and Cu^+^ are the most effective interacting cations, as determined by their coordination with the coordinative site exhibiting the best coordination with the crown ether or thioether binding sites.

The EDA-NOCV results reveal that the stability of the resulting interaction is maximized when the cavity size closely matches the ionic radius of the guest cation, a principle known as the size match rule, demonstrating that the environment of the binding site plays a significant role in this process, as evidenced by changing the crown ether to a crown thioether moiety, on going from **IO** to **IS**, leading in an increase in the binding energy stabilization when coinage metal cations interact, and a noticeable decrease in interaction with all alkali metals interact, but still exhibiting the strongest interactions with Li^+^ and Cu^+^ among all alkali and coinage metal cations, respectively. The EDA-NOCV, in conjunction with the geometric parameters, elucidates that both the size of the cation, influenced by the CN, and cavity size of the crown ether/thioether portions are main key factors modulating the cation recognition.

## Supplementary Material



## Notes

Figure S1**:** 3D Visual representation of the modified [2]­catenanes (**IO** and **IS**) Figure S2**:** Depicted distances between the interacting cation and the binding environment (**d3-d7**), and **dCI** if the counterion is applied Figure S3**:** 3D visual representation of the Optimized **IO**-M^2+^. ${{\mathrm{PF}}_{6}}^{\text{−}}$ and **IS**-M^2+^. ${{\mathrm{PF}}_{6}}^{\text{−}}$ structures Figure S4 - S7:3D visual representation of the first two density deformation channel surface plots of all remaining studied interactions from structures **IO**, **IS** and **TeO** Table S1–S19**:** Cartesian Coordenantes of all studied structures **IO**-M^+^.X^–^, **IS**-M^+^.X^–^, **IO**-M^2+^.X^–^. ${{\mathrm{PF}}_{6}}^{\text{−}}$ and IO-M^2+^.X^–^. ${{\mathrm{PF}}_{6}}^{\text{−}}$ (PDF)
