# Supplementary material for: Shedding Light on the Capabilities of Heteroditopic Mechanically Interlocked Molecules in Ion-Pair Sensing
Source: J Phys Chem A. 2026 Feb 5;130(7):1501–13. doi: 10.1021/acs.jpca.5c08037 (PMC12927025; doi:10.1021/acs.jpca.5c08037)
Supplement: Supplementary file 1 [file jp5c08037_si_001.pdf]

Supporting Information: Shedding Light on the Capabilities of Heteroditopic Mechanically Interlocked Molecules in Ion-Pair Sensing

Fábio J. Amorim,[*,†] Felipe R. F. Pagliarini,[†] Renato L. T. Parreira,[‡] and Giovanni F. Caramori[*,†]

†*Departamento de Química, Universidade Federal de Santa Catarina, Campus Universitário Trindade, 88040-900, Florianópolis, SC, Brazil.*

‡*Núcleo de Pesquisas em Ciências Exatas e Tecnológicas, Universidade de Franca, 14404-600, Franca, SP, Brazil*

E-mail: fabio.amorim@posgrad.ufsc.br; giovanni.caramori@ufsc.br

Contents

Illustrations **S3**

Cartesian Coordinates **S21**

Supporting Information Available

In a nutshell, the first section of the SI available includes the illustrations referencing both modified structures **IS-TeO** in S1, the distance of the applied counter anion, PF_6^- with the M^{2+} cations, referring to **dCI**, in S2, the optimized geometries of both **IO-IS** [2]catenanes interacting with the M^{2+} cations and the counter anion, PF_6^-, in S3 and the density deformation channels of all remaining structures obtained from the NOCV analysis in S4-S7. The second section provides the Cartesian Coordenates of the obtained optimized structures, utilized for the analysis, of all studied systems.

Illustrations

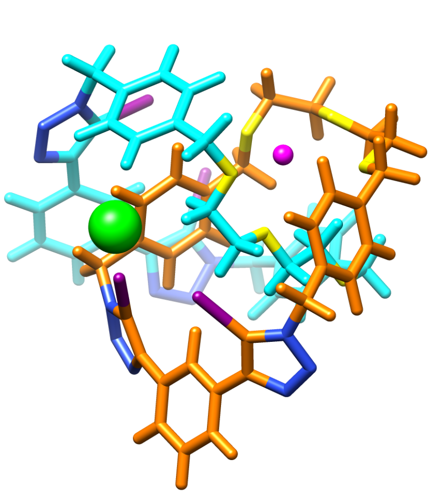

(a)

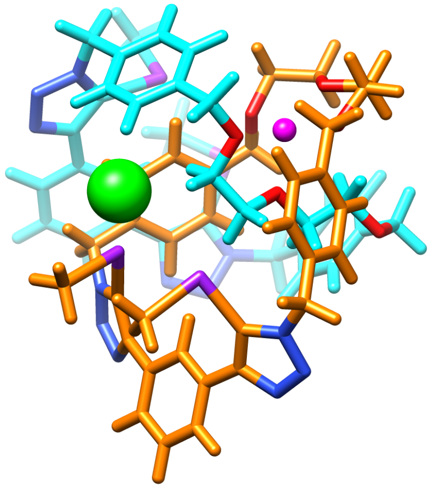

(b)

Figure S1: Modified [2]Catenanes **2-3**; **a)** 3D representation of modified Heteroditopic structure nominated as **2**, where the oxygen atoms were replaced by sulfur atoms (highlighted in yellow); **b)** 3D representation of the Heteroditopic structure nominated as **3**, where both original $\sigma-$hole interaction donor atoms (I) were replaced by $-Te-CH_3$

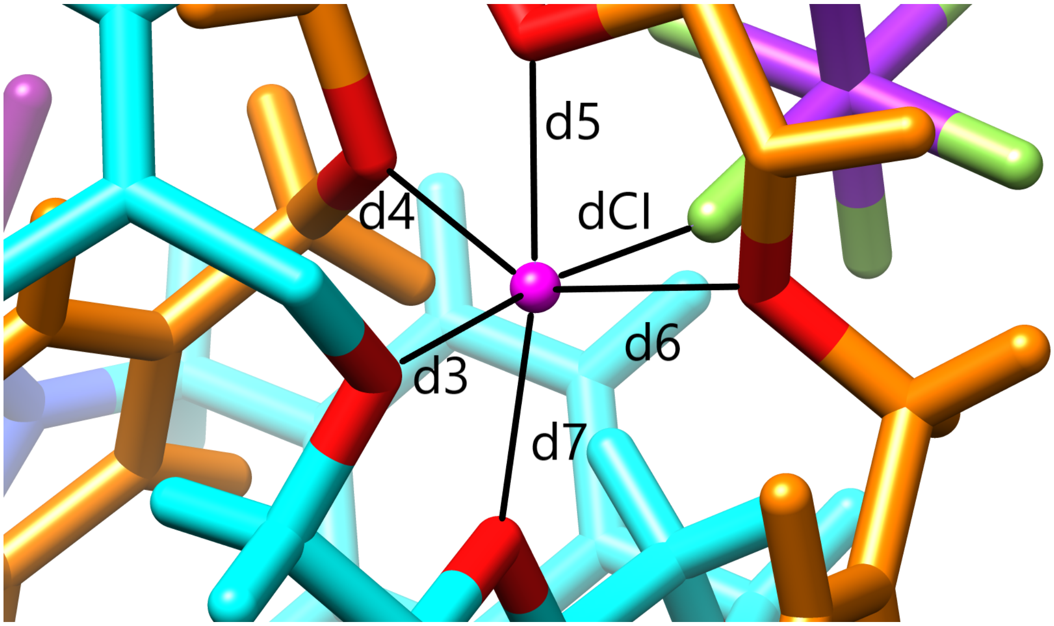

Figure S2: All depicted distances between the interacting cation and the binding environment (**d3-d7**), with the inclusion of **dCI** referring to the distance between the cation and the counter-ion (if applied)

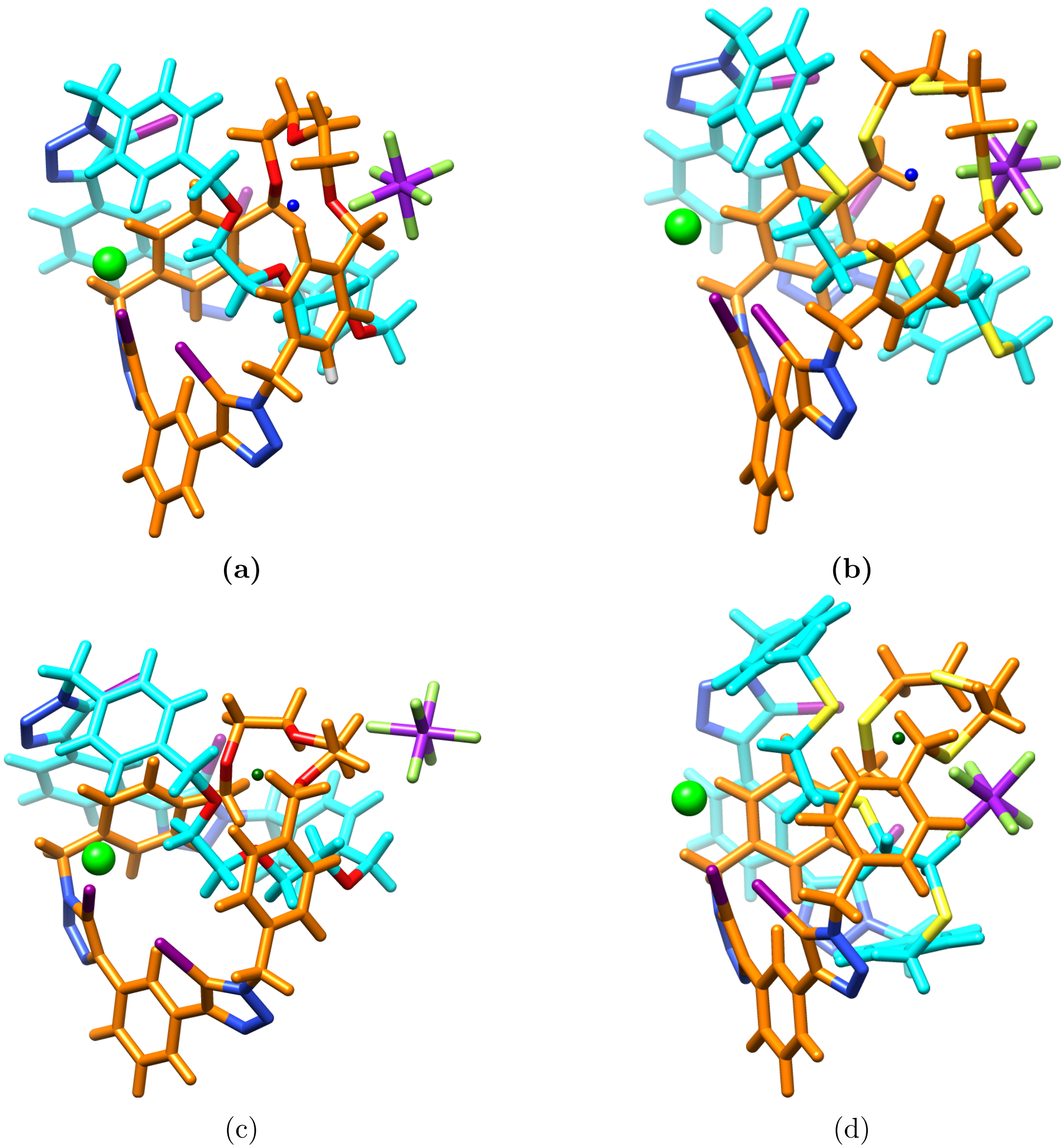

Figure S3: Optimized **IO**-$M^{2+}.PF_6{}^-I^-$ and **IS**-$M^{2+}.PF_6{}^-I^-$ structures: **a) IO**-$Zn^{2+}.PF_6{}^-I^-$; **b)** IO-$Ni^{2+}.PF_6{}^-I^-$; **c) IS**-$Zn^{2+}.PF_6{}^-I^-$; **d) IS**-$Ni^{2+}.PF_6{}^-I^-$

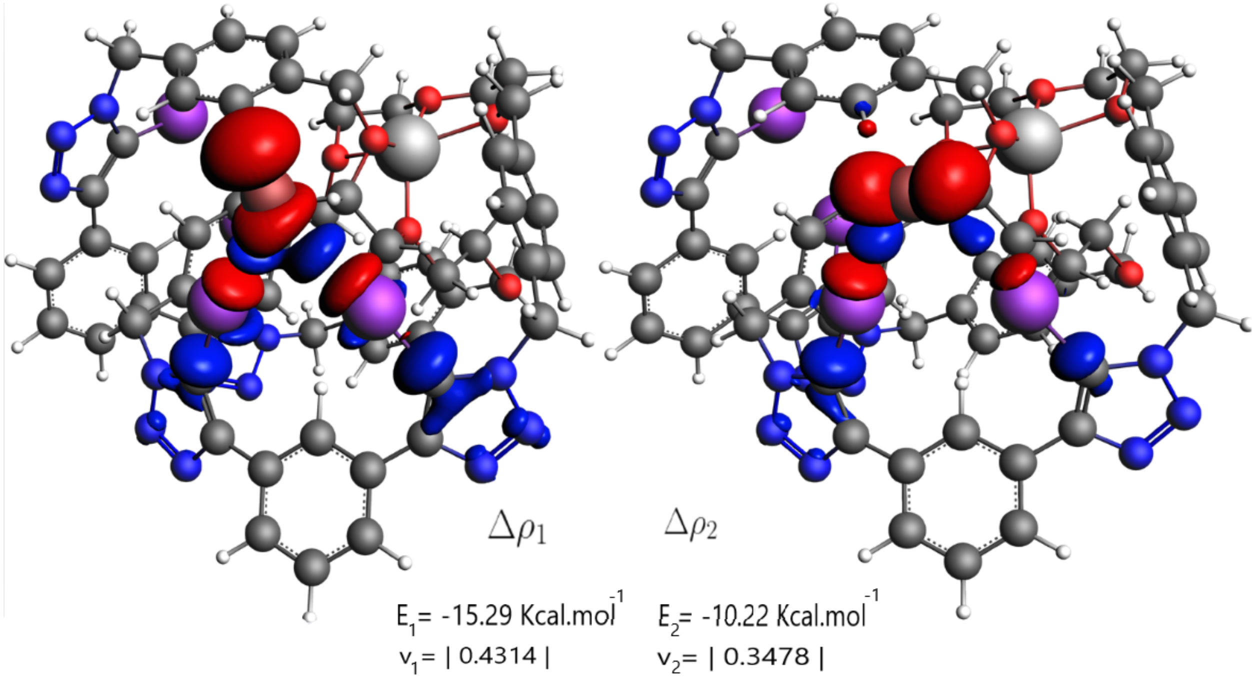

IO-Li$^+$···Br$^-$

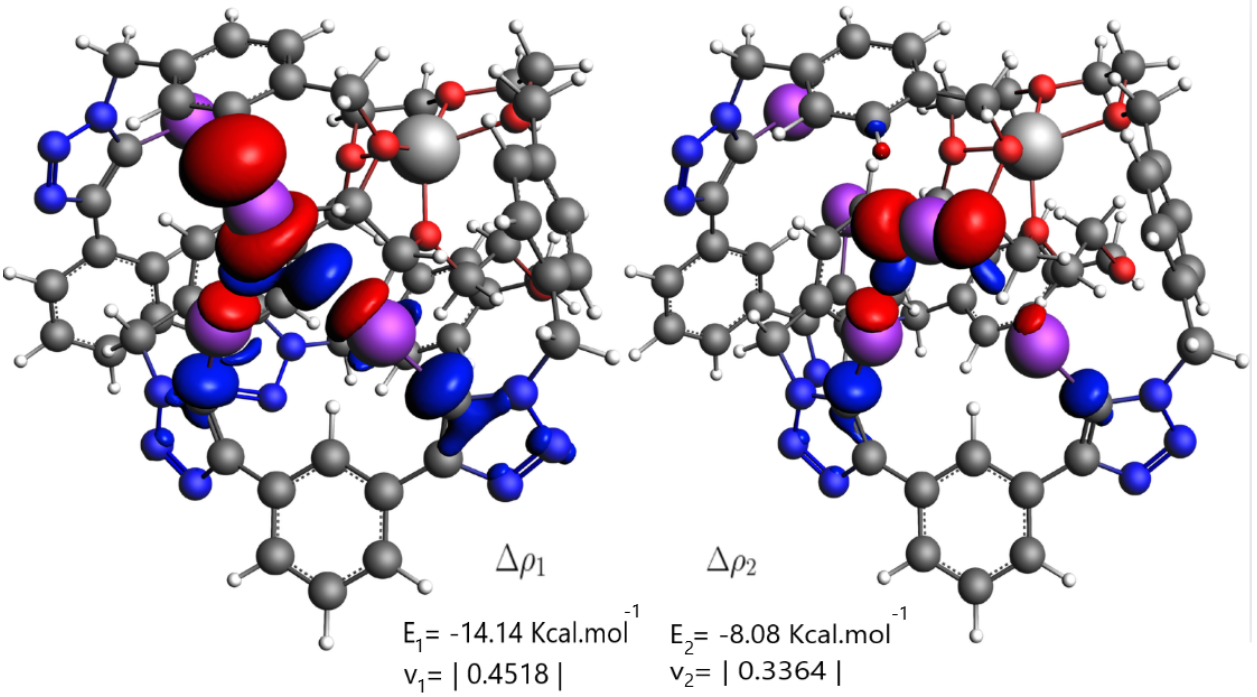

IO-Li$^+$···I$^-$

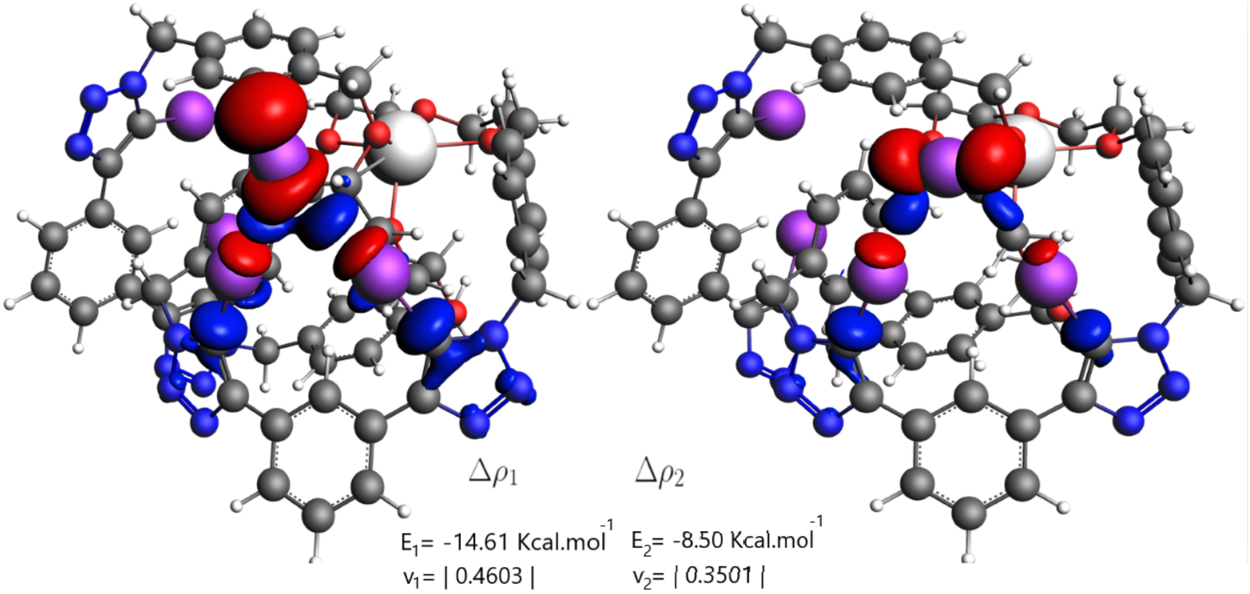
$\Delta\rho_1$
$\Delta\rho_2$
E_1= -14.61 Kcal.mol^{-1}
E_2= -8.50 Kcal.mol^{-1}
v_1= | 0.4603 |
v_2= | 0.3501 |

IO-Na^+···I^-

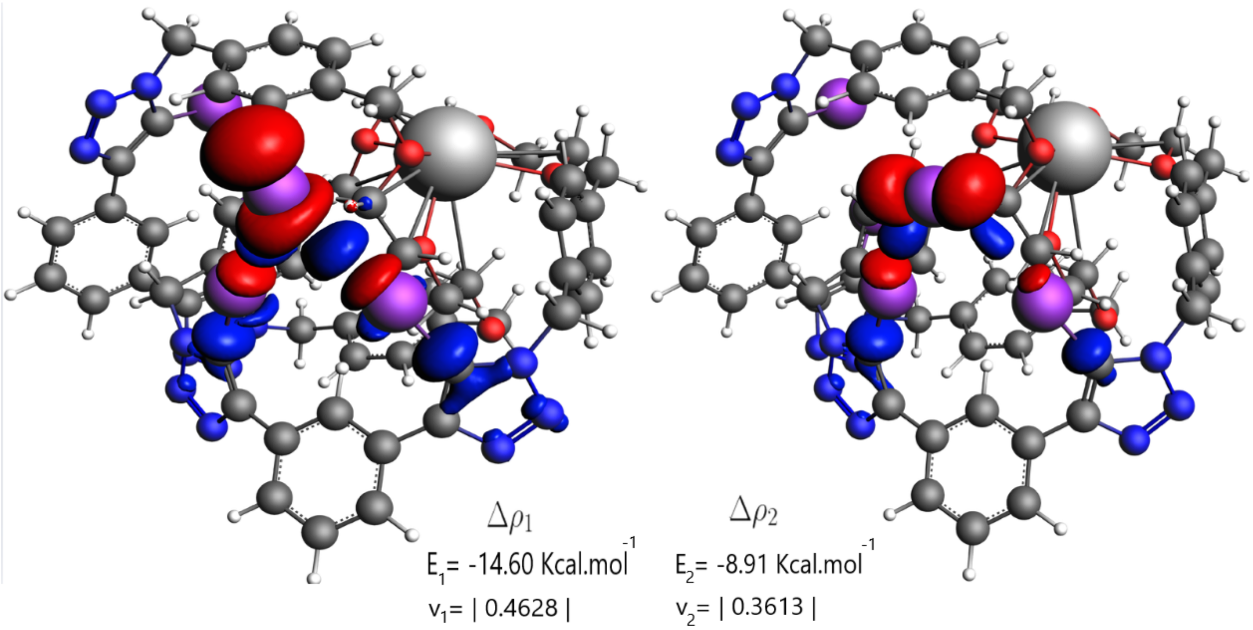
$\Delta\rho_1$
$\Delta\rho_2$
E_1= -14.60 Kcal.mol^{-1}
E_2= -8.91 Kcal.mol^{-1}
v_1= | 0.4628 |
v_2= | 0.3613 |

IO-K^+···I^-

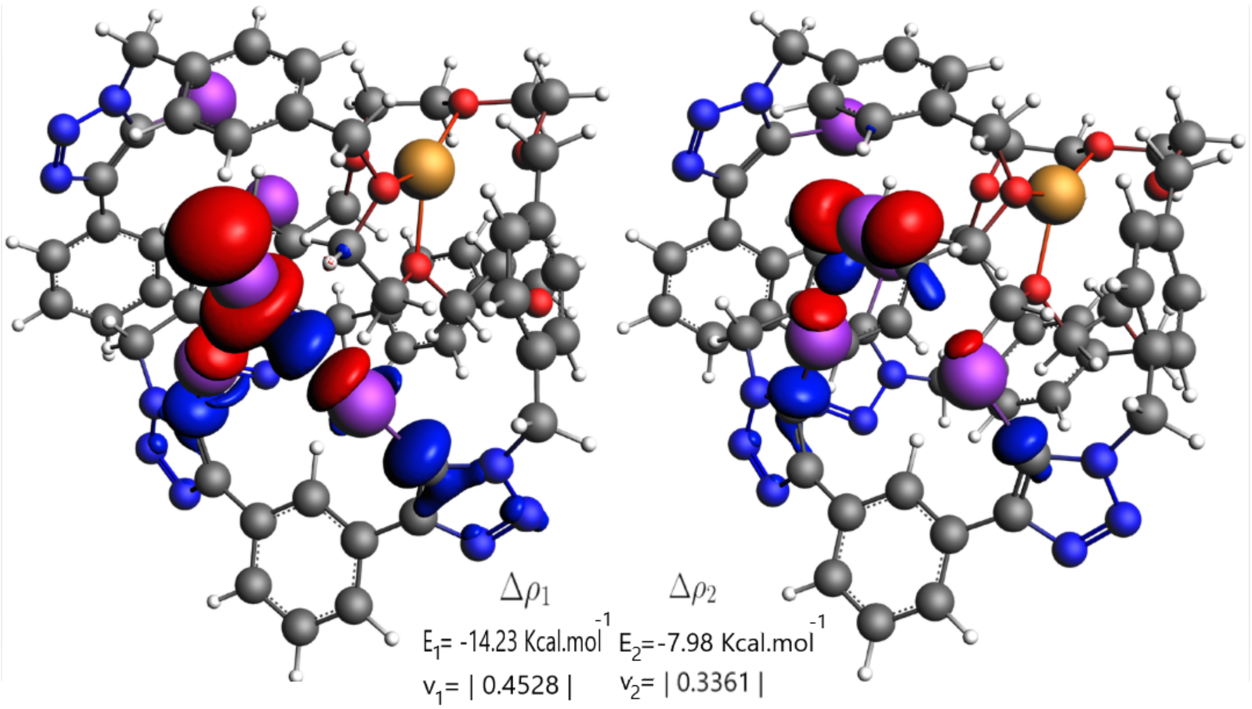

IO-$Cu^{+}\cdots I^{-}$

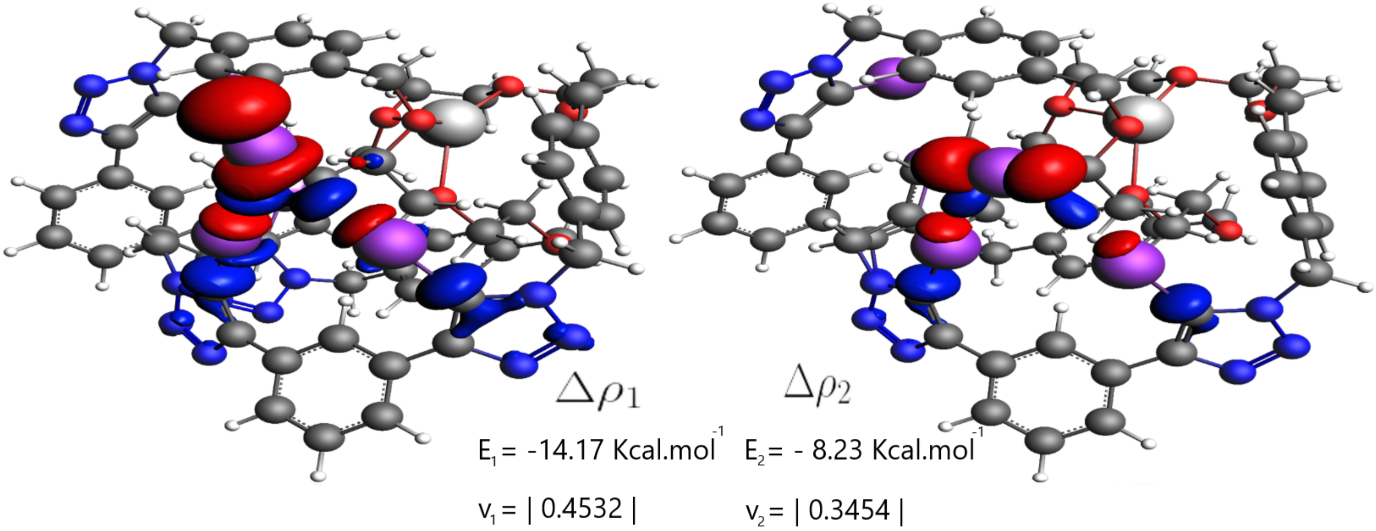

IO-$Ag^{+}\cdots I^{-}$

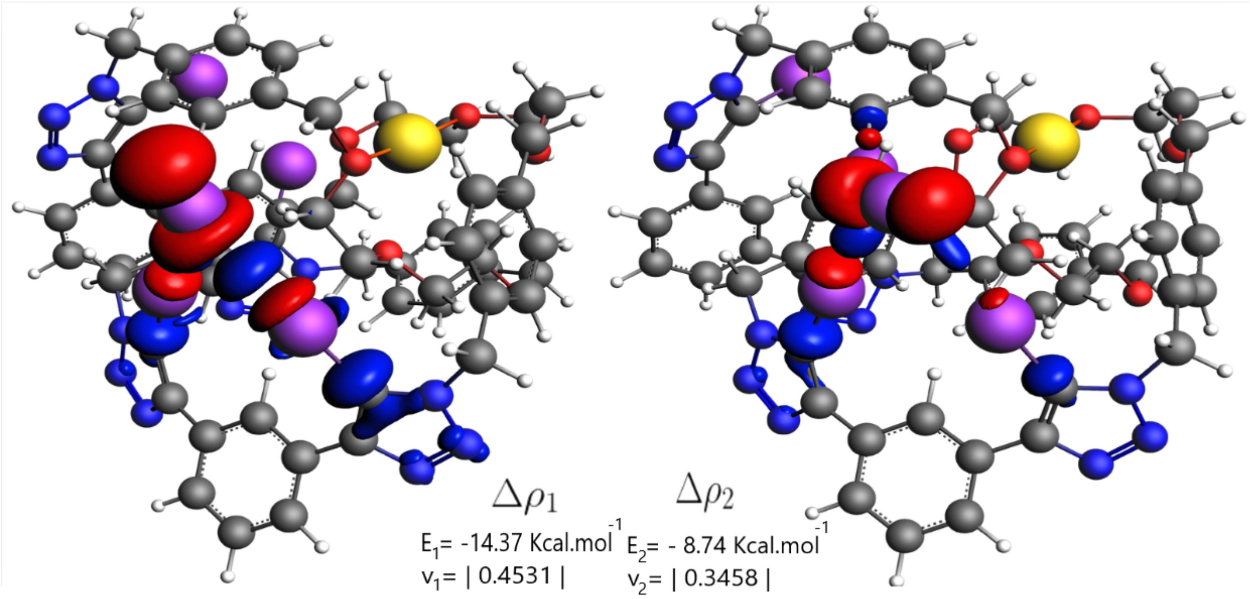

IO-Au^{+}···I^{-}

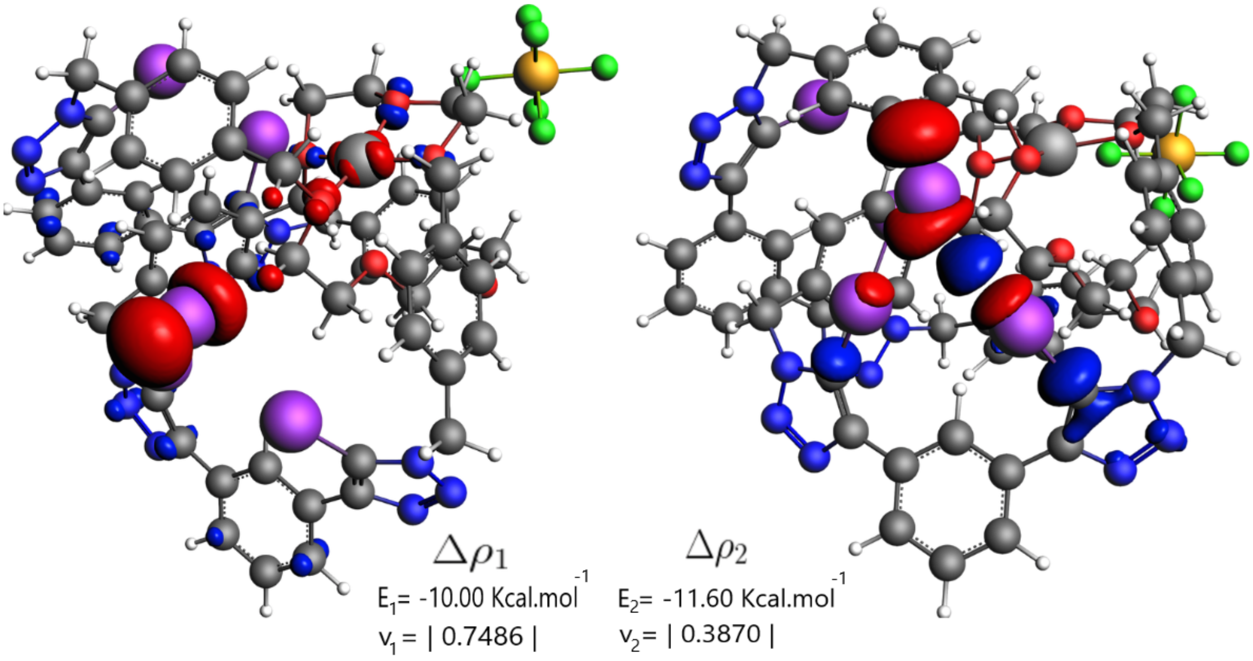

IO-Ni^{2+}···I^{-}

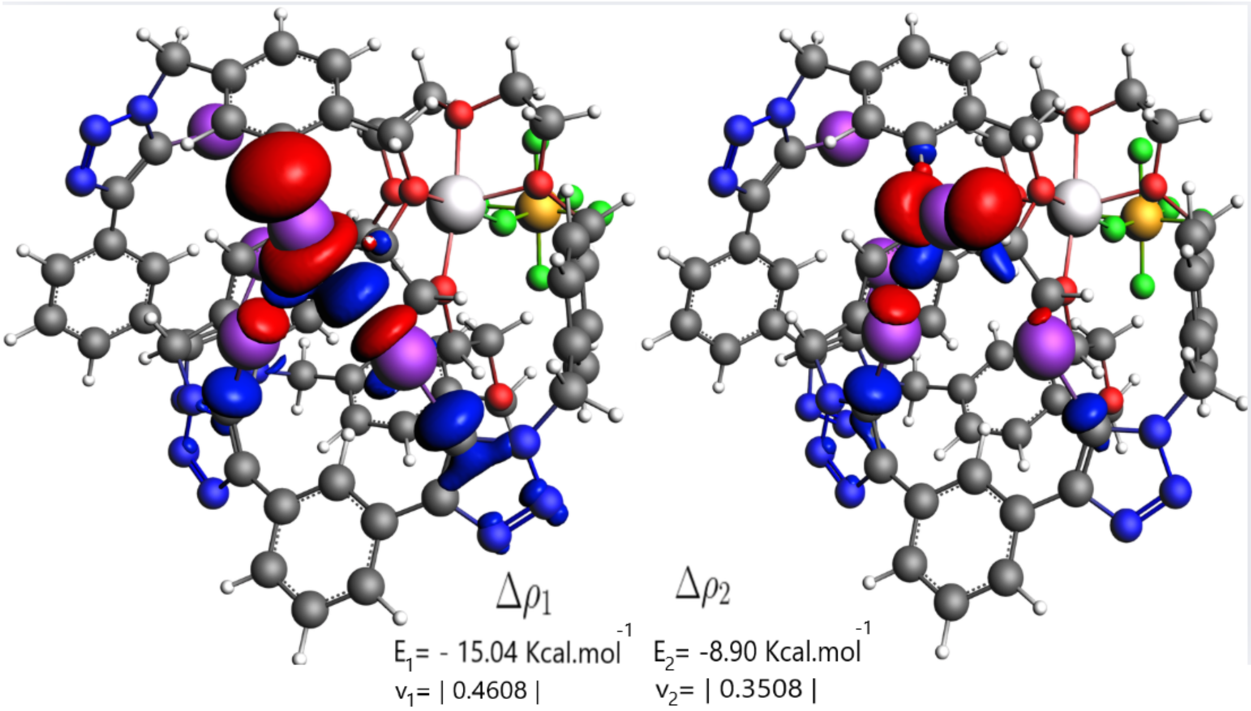

IO-Zn^{2+}···I^{-}

Figure S4: The first two density deformation channel surface plots, $\rho_{1,2}$ with isovalue: 0.001 a.u., where the red and blue regions indicate the outflow and inflow electron density for the anion interaction of the **IO** structures

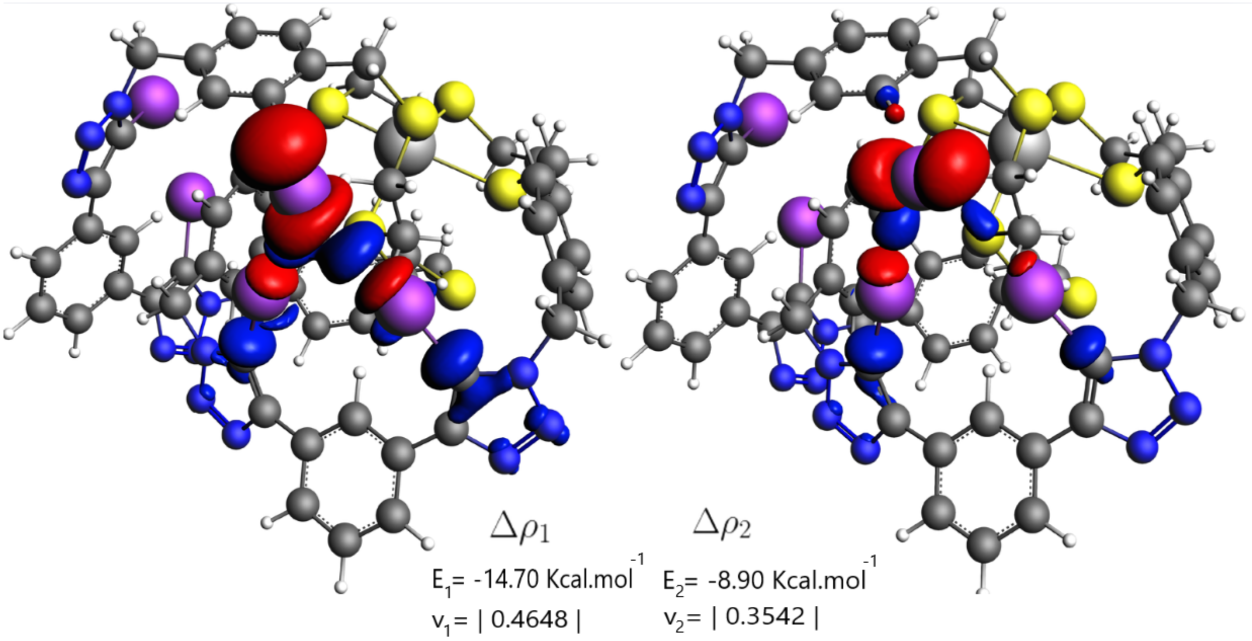

Δρ1
Δρ2
E1= -14.70 Kcal.mol-1
v1= | 0.4648 |
E2= -8.90 Kcal.mol-1
v2= | 0.3542 |

IS-Li$^+$···I$^-$

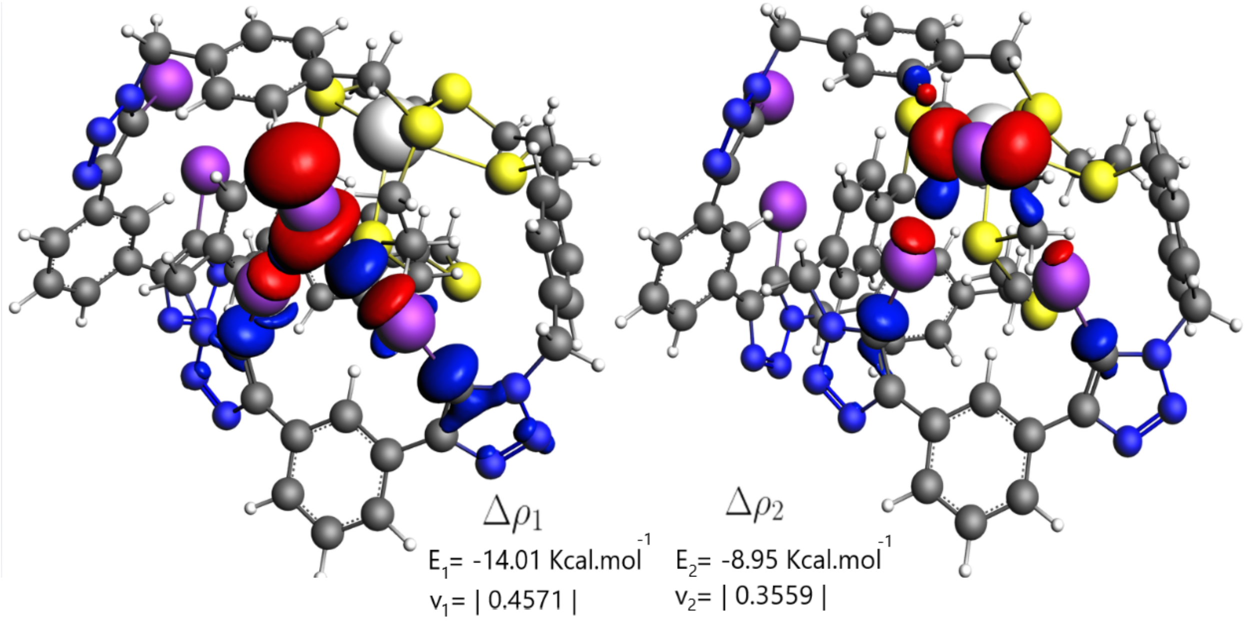

Δρ1
Δρ2
E1= -14.01 Kcal.mol-1
v1= | 0.4571 |
E2= -8.95 Kcal.mol-1
v2= | 0.3559 |

IS-Na$^+$···I$^-$

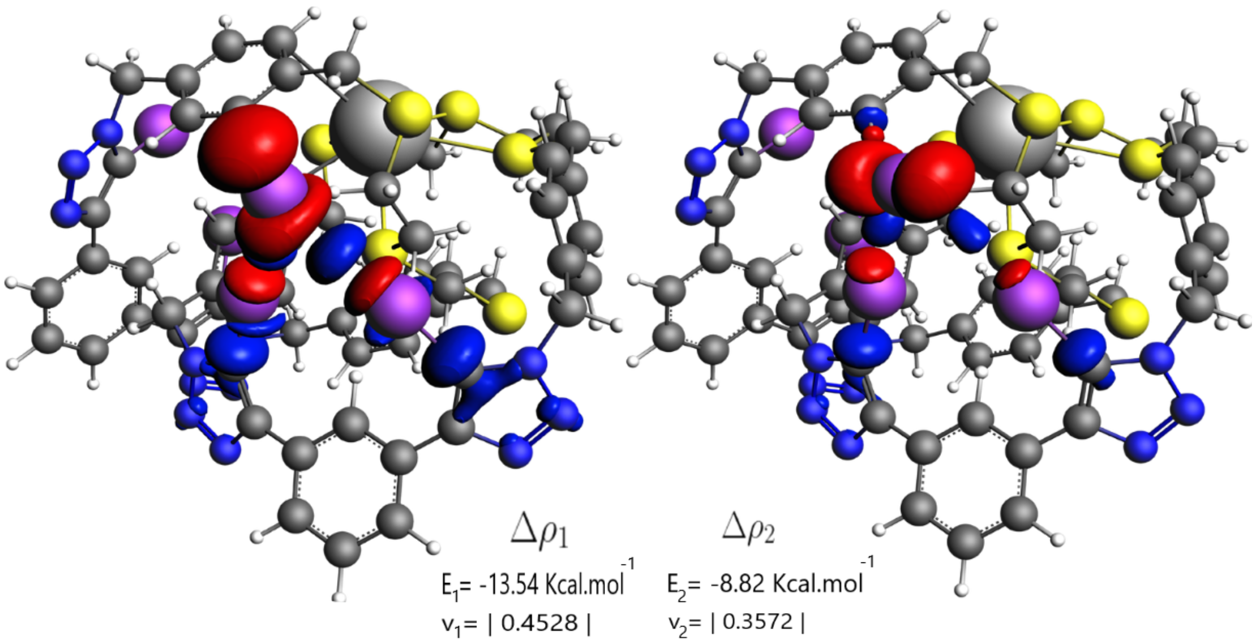

IS-$K^+ \cdots I^-$

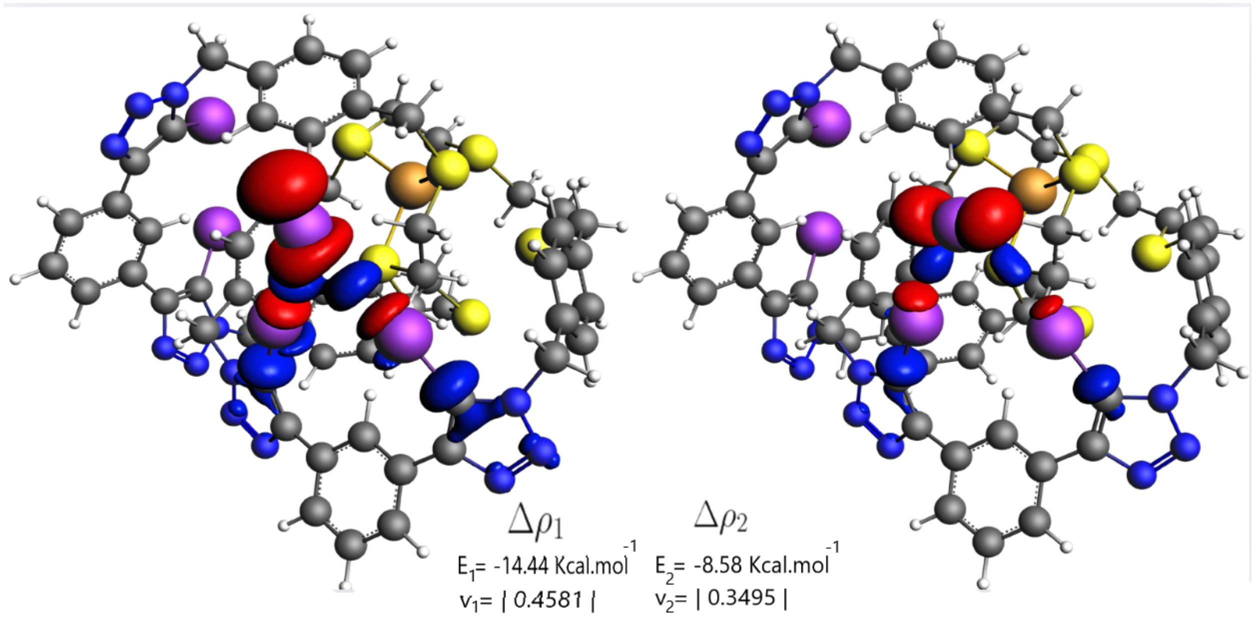

IS-$Cu^+ \cdots I^-$

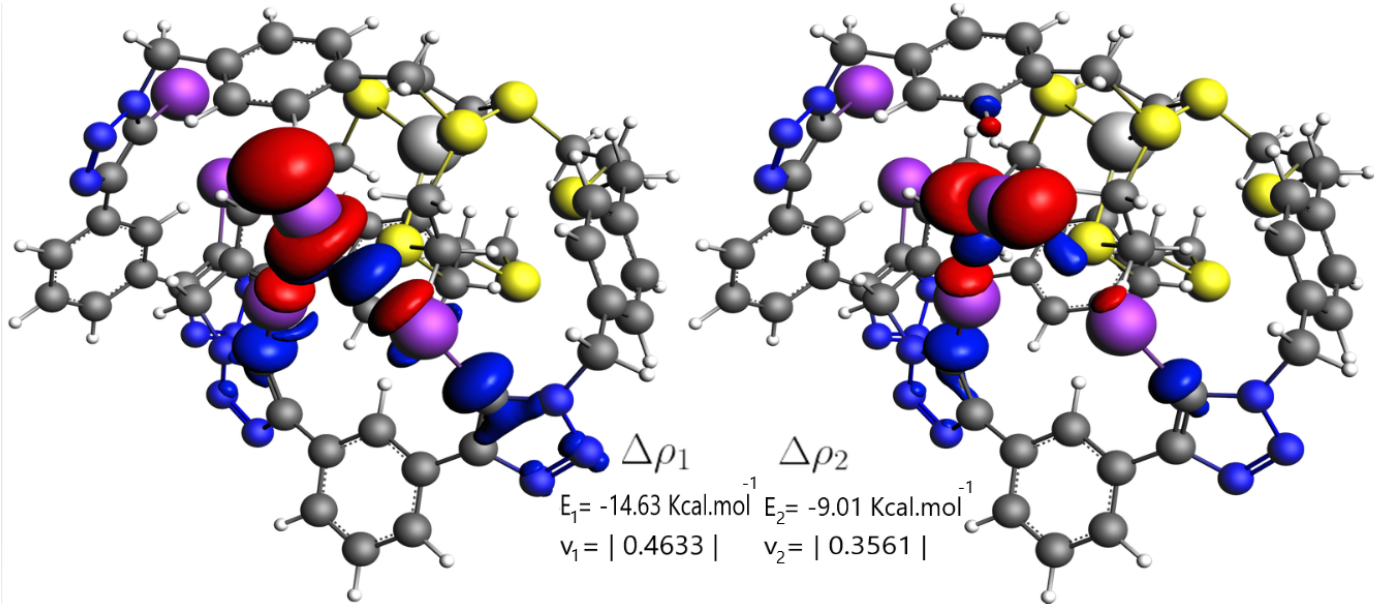

IS-Ag$^+\cdots$I$^-$

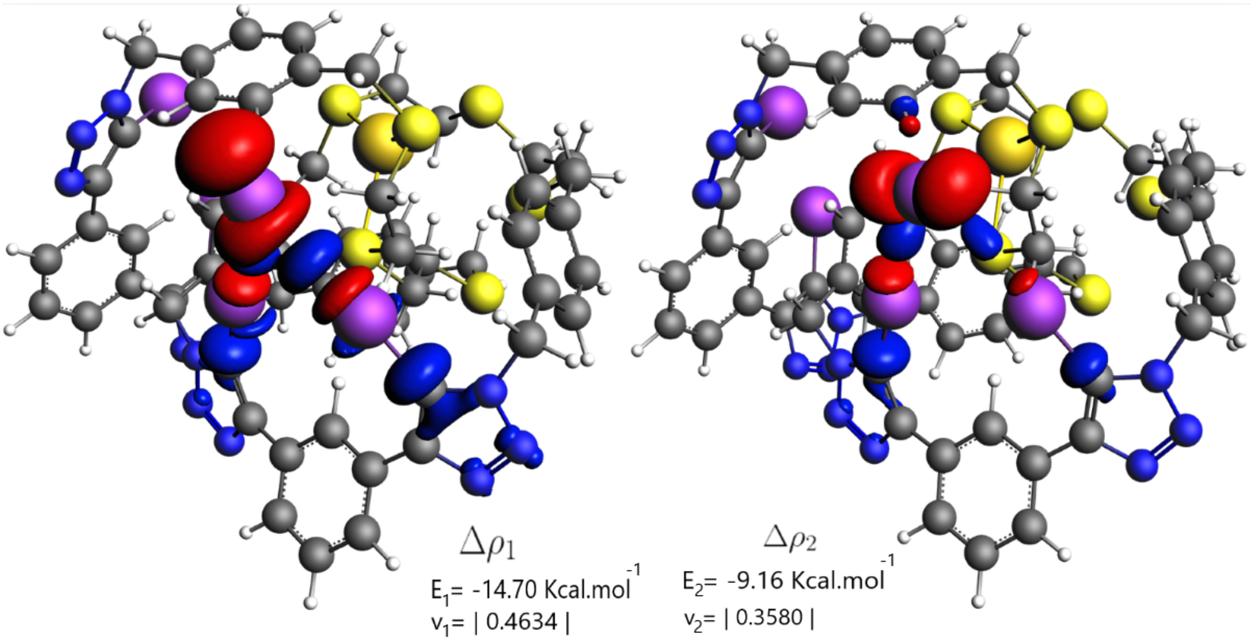

IS-Au$^+\cdots$I$^-$

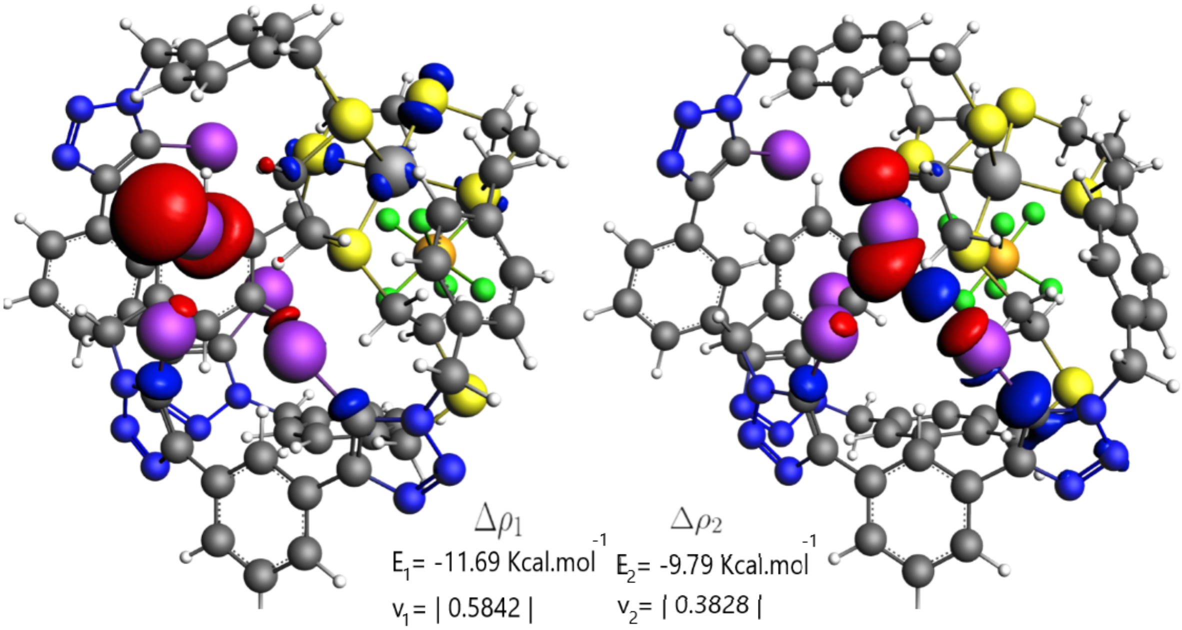

IS-Ni^{2+}···I^{-}

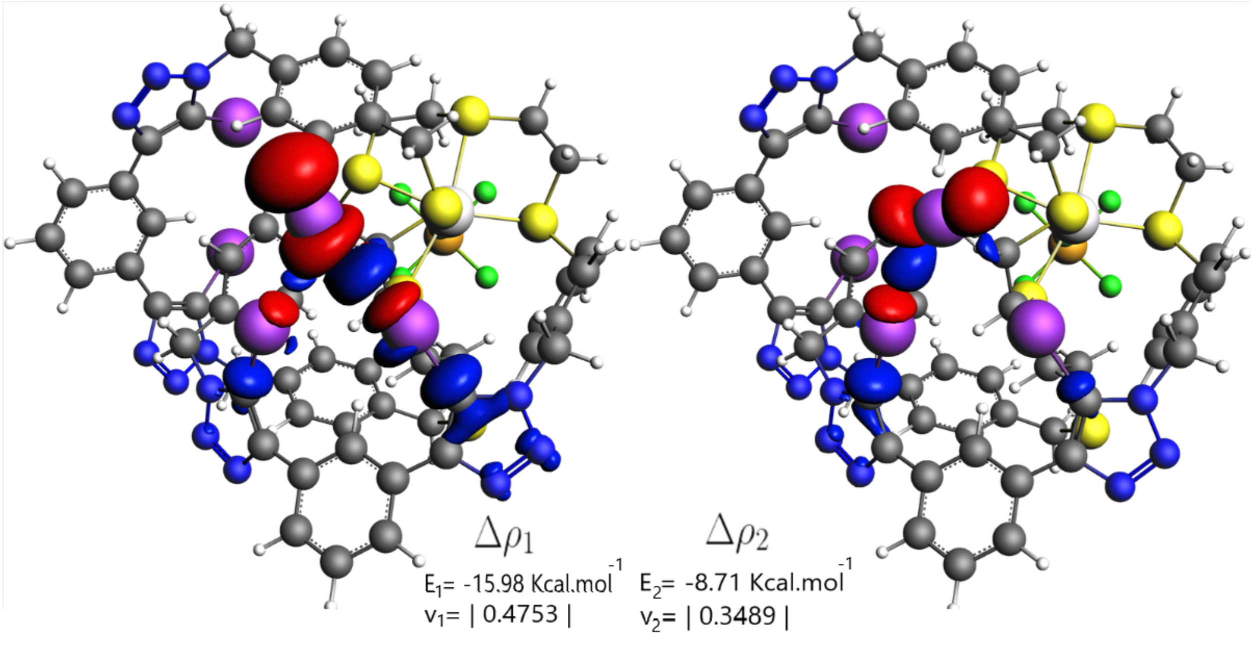

IS-Zn^{2+}···I^{-}

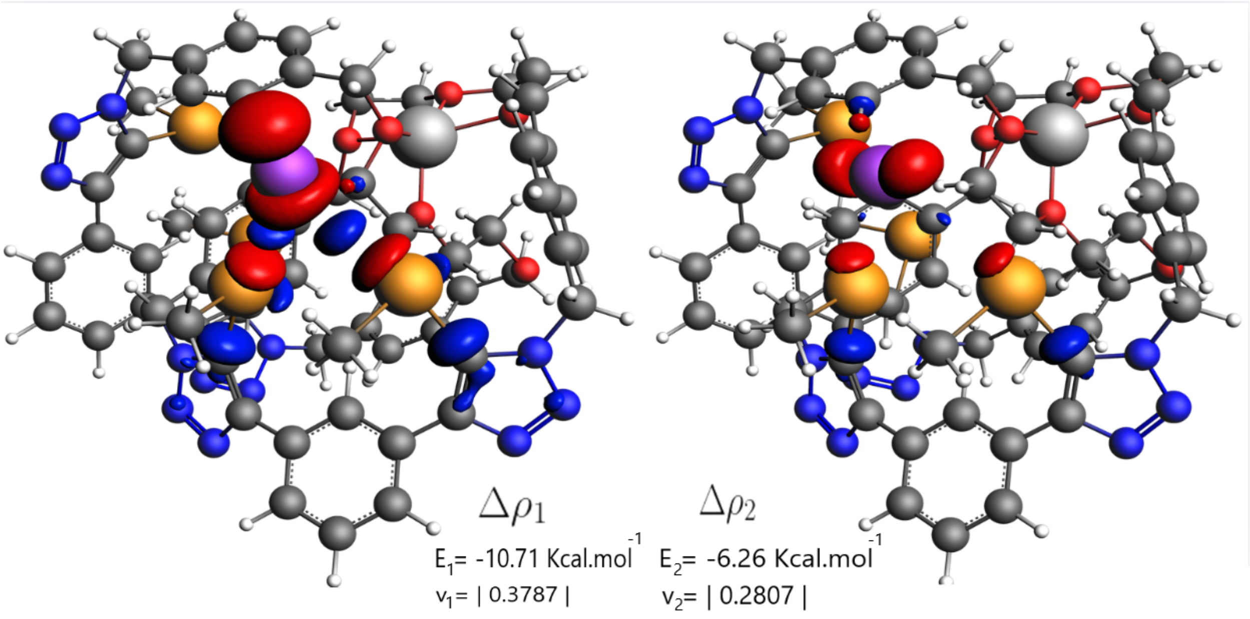

TeO-Li$^{+}\cdots$I^{-}

Figure S5: The first two density deformation channel surface plots, $\rho_{1,2}$ with isovalue: 0.001 a.u., where the red and blue regions indicate the outflow and inflow electron density for the anion interaction of the **IS** and **TeO** structures

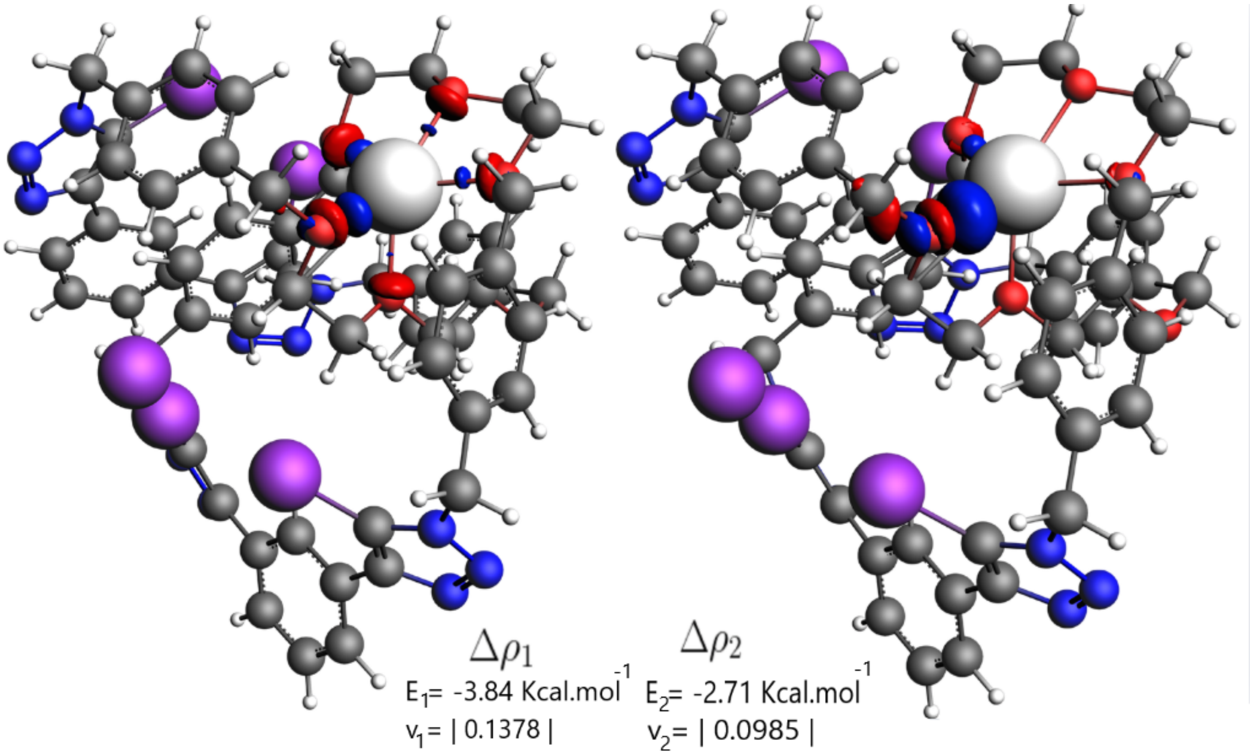

IO-I$^{-}\cdots$Na^{+}

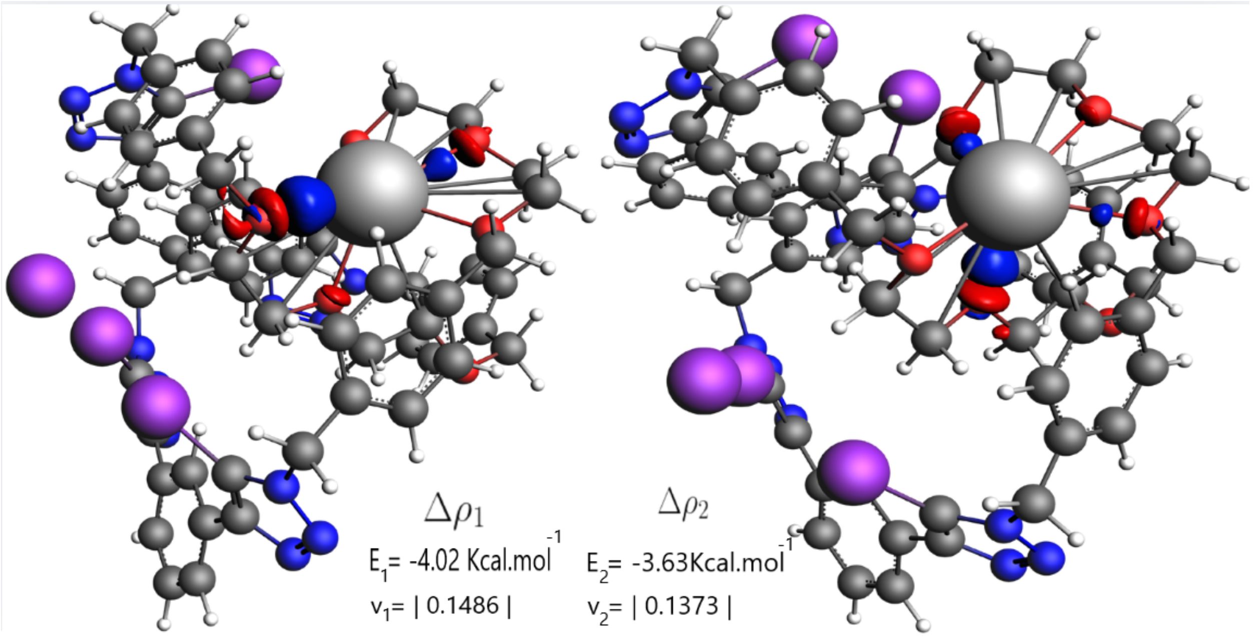

IO-I$^{-}\cdots$K^{+}

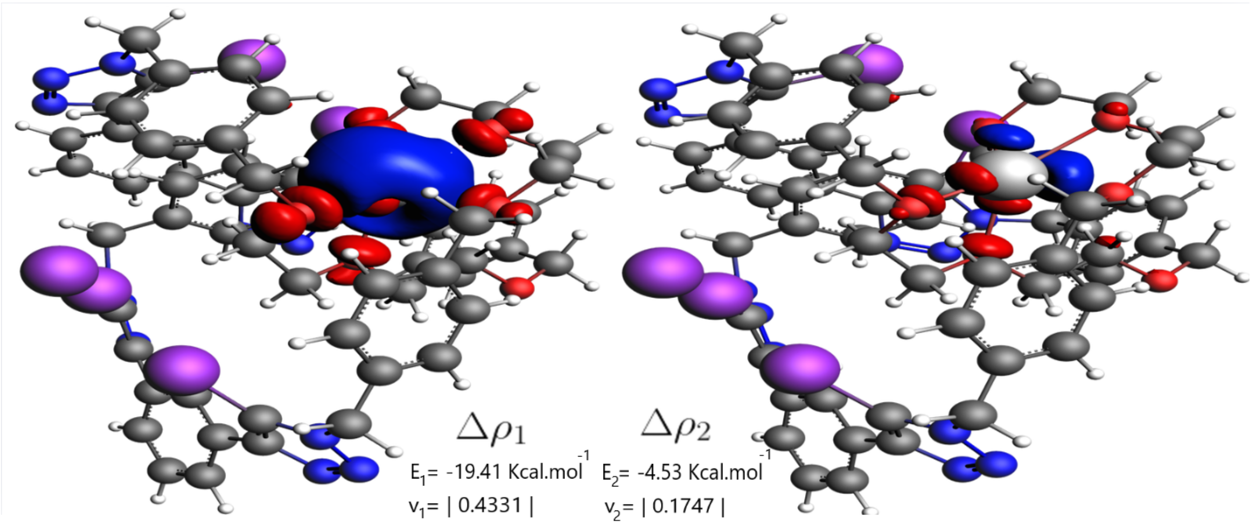

IO-I$^{-}\cdots$Ag^{+}

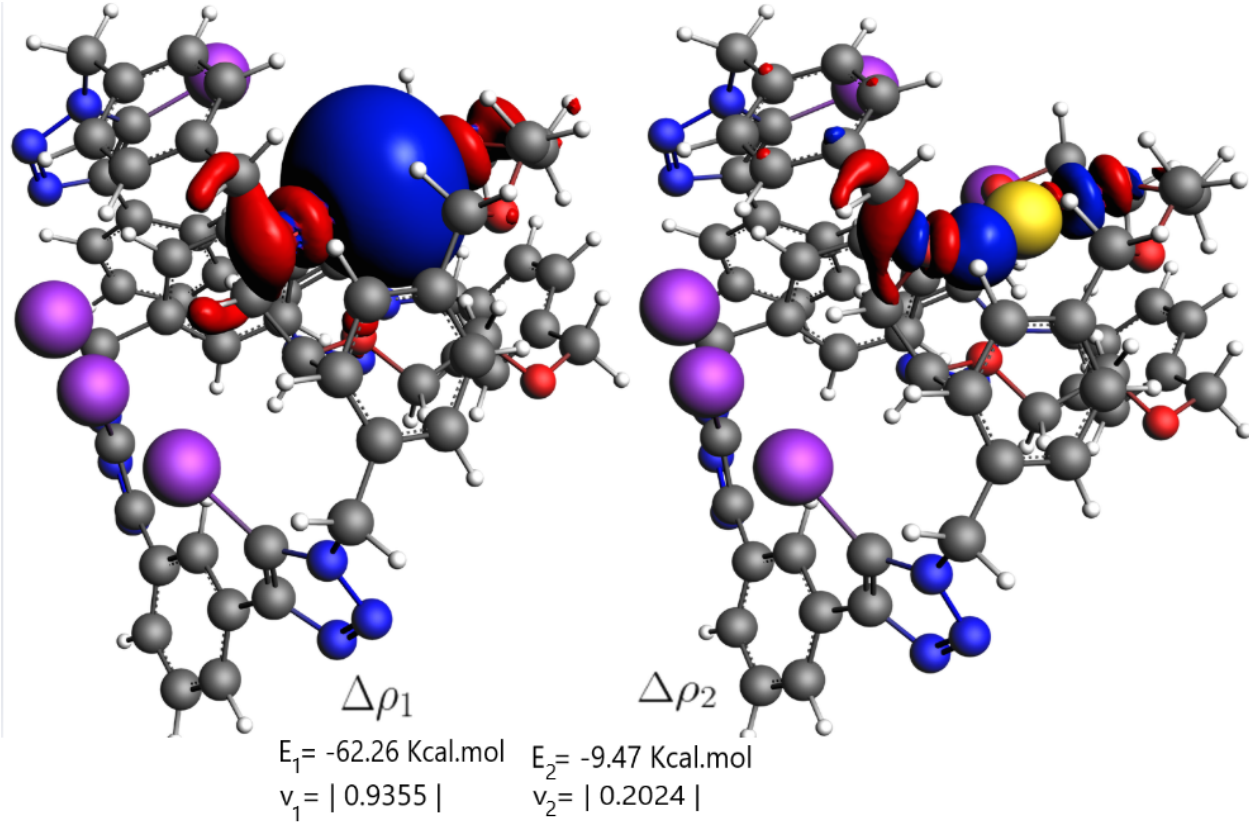

IO-I$^{-}\cdots$Au^{+}

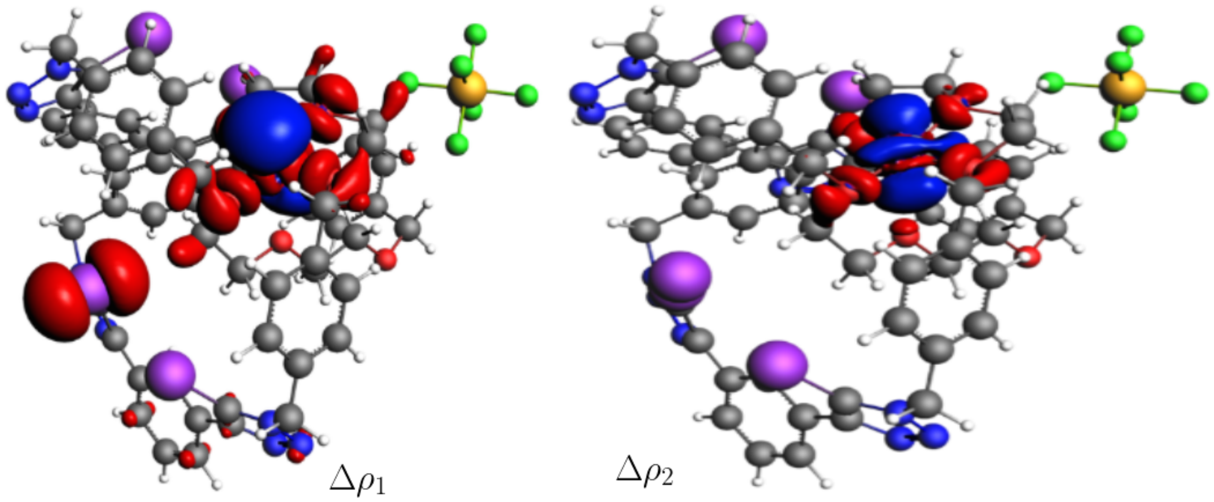

$\Delta\rho_1$ $\Delta\rho_2$

E_1= +234.13 Kcal.mol^{-1} E_2= -53.77 Kcal.mol^{-1}

v_1 = | 1.7829 | v_2= | 0.4277 |

IO-I$^-$···Ni^{2+}

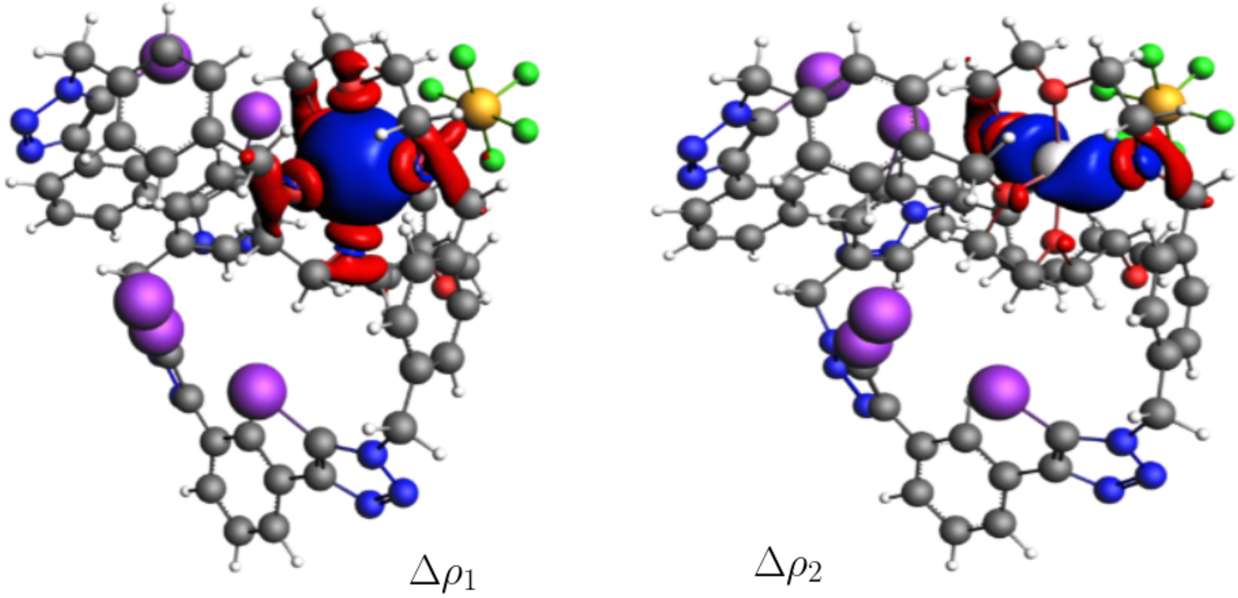

$\Delta\rho_1$ $\Delta\rho_2$

E_1= -56.82 Kcal.mol^{-1} E_2= -21.87 Kcal.mol^{-1}

v_1= | 0.5411 | v_2= | 0.2740 |

IO-I$^-$···Zn^{2+}

Figure S6: The first two density deformation channel surface plots, $\rho_{1,2}$ with isovalue: 0.001 a.u., where the red and blue regions indicate the outflow and inflow electron density for the cation interaction of the **IO** structures

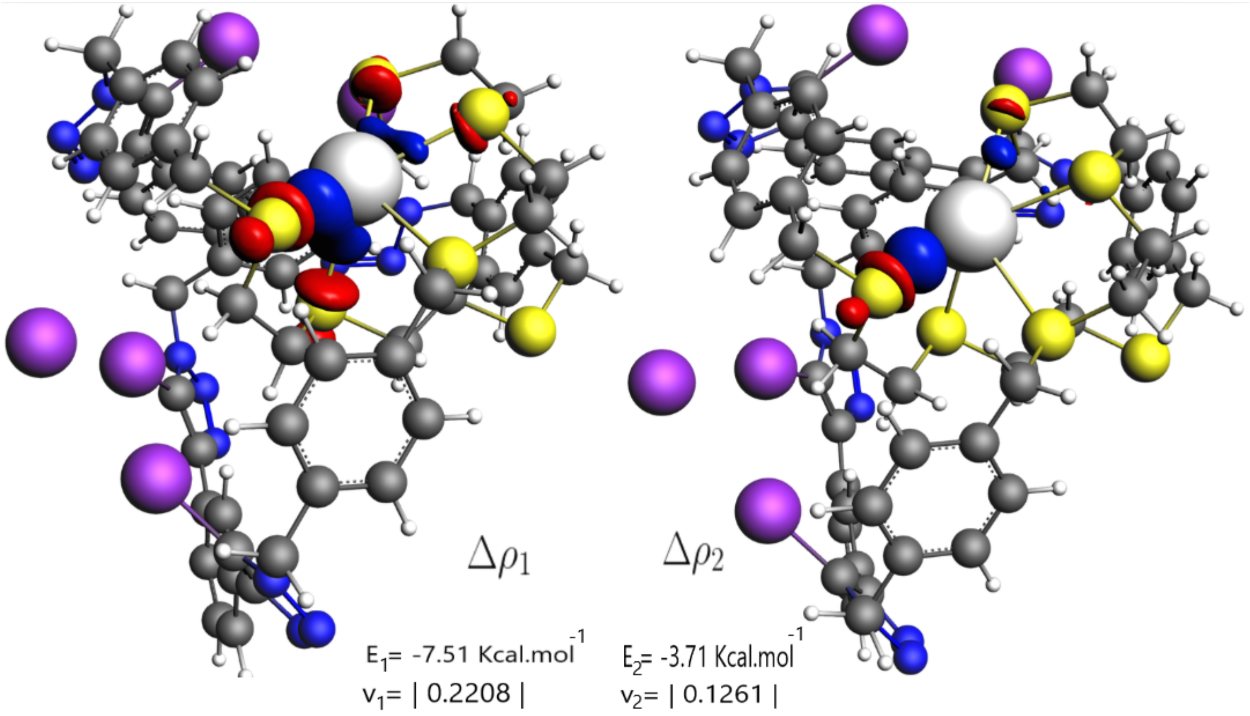
Δρ1
Δρ2
E1= -7.51 Kcal.mol-1
v1= | 0.2208 |
E2= -3.71 Kcal.mol-1
v2= | 0.1261 |

IS-I$^-$···NA$^+$

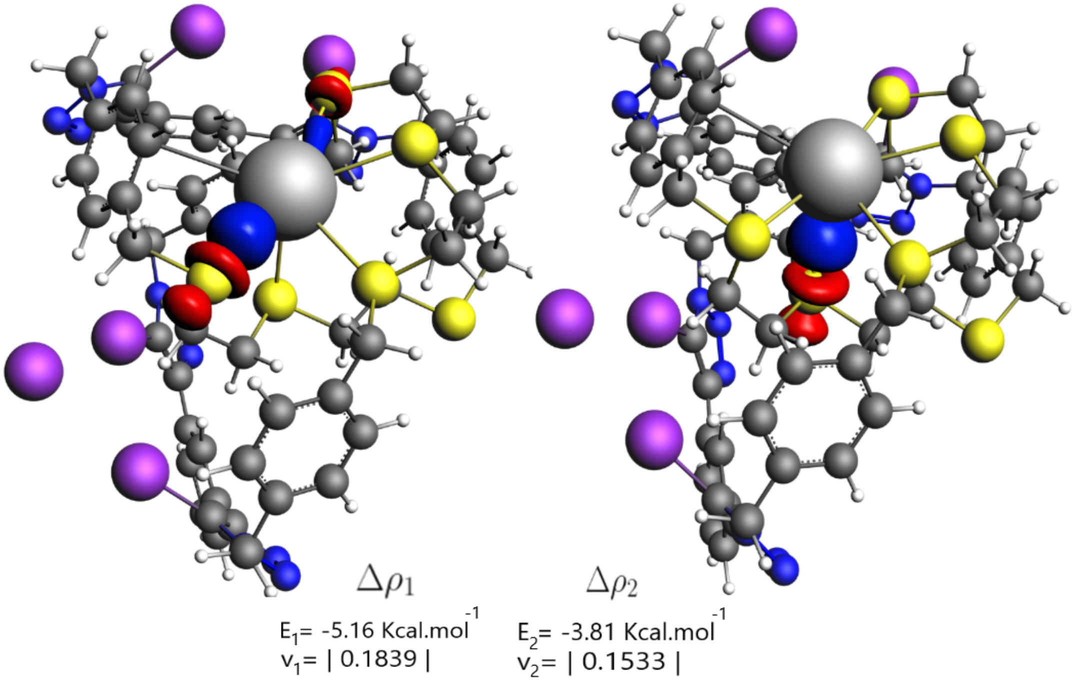
Δρ1
Δρ2
E1= -5.16 Kcal.mol-1
v1= | 0.1839 |
E2= -3.81 Kcal.mol-1
v2= | 0.1533 |

IS-I$^-$···K$^+$

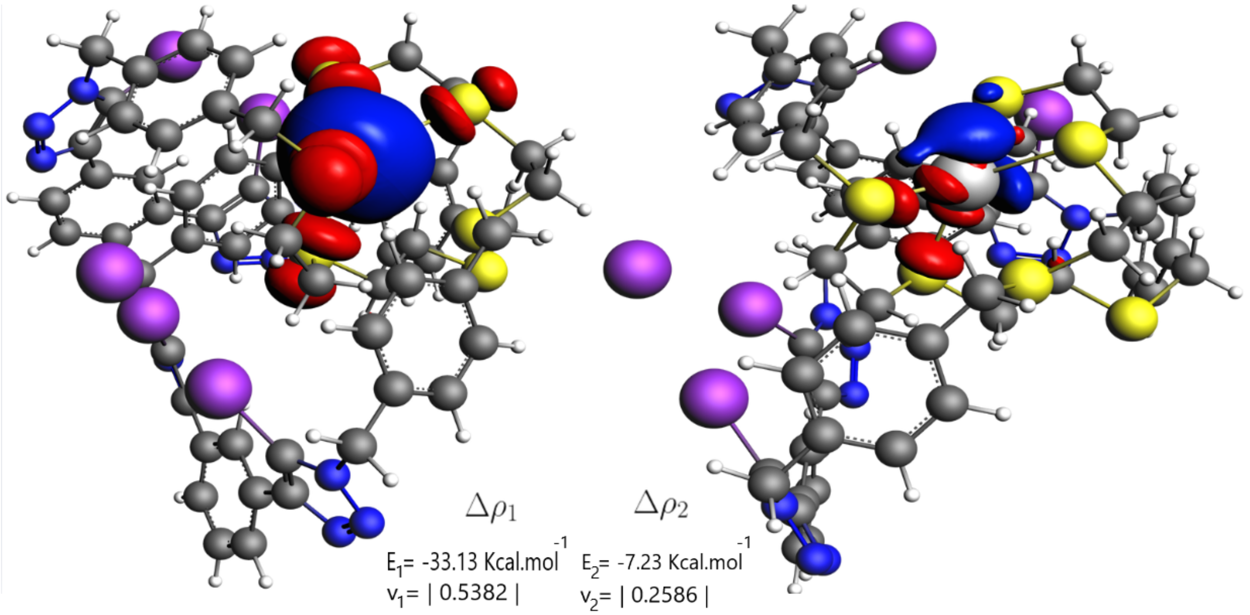

$\Delta\rho_1$
$\Delta\rho_2$
E_1= -33.13 Kcal.mol^{-1}
v_1= | 0.5382 |
E_2= -7.23 Kcal.mol^{-1}
v_2= | 0.2586 |

IS-I$^-$···Ag$^+$

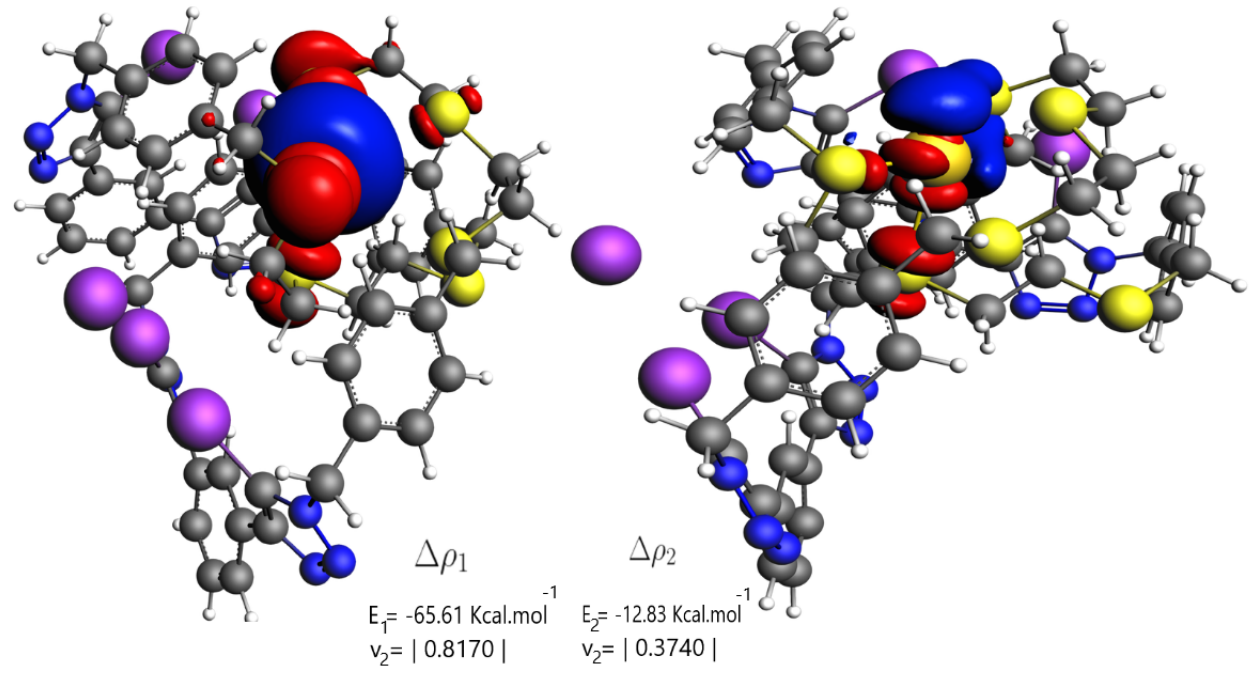

$\Delta\rho_1$
$\Delta\rho_2$
E_1= -65.61 Kcal.mol^{-1}
v_2= | 0.8170 |
E_2= -12.83 Kcal.mol^{-1}
v_2= | 0.3740 |

IS-I$^-$···Au$^+$

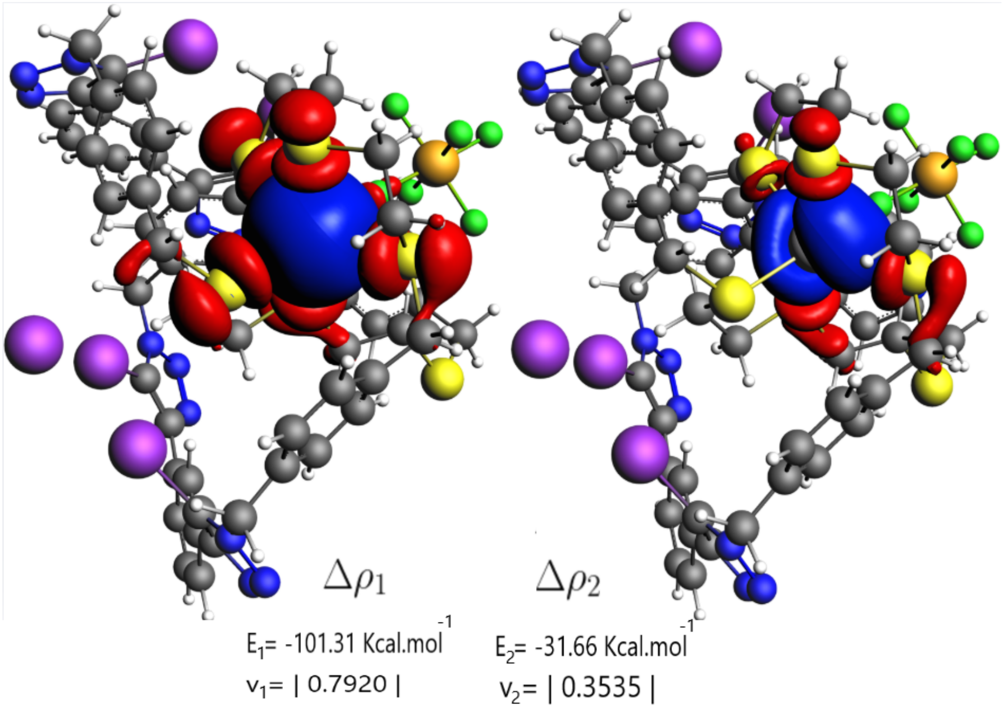

IS-I$^-$···Zn^{2+}

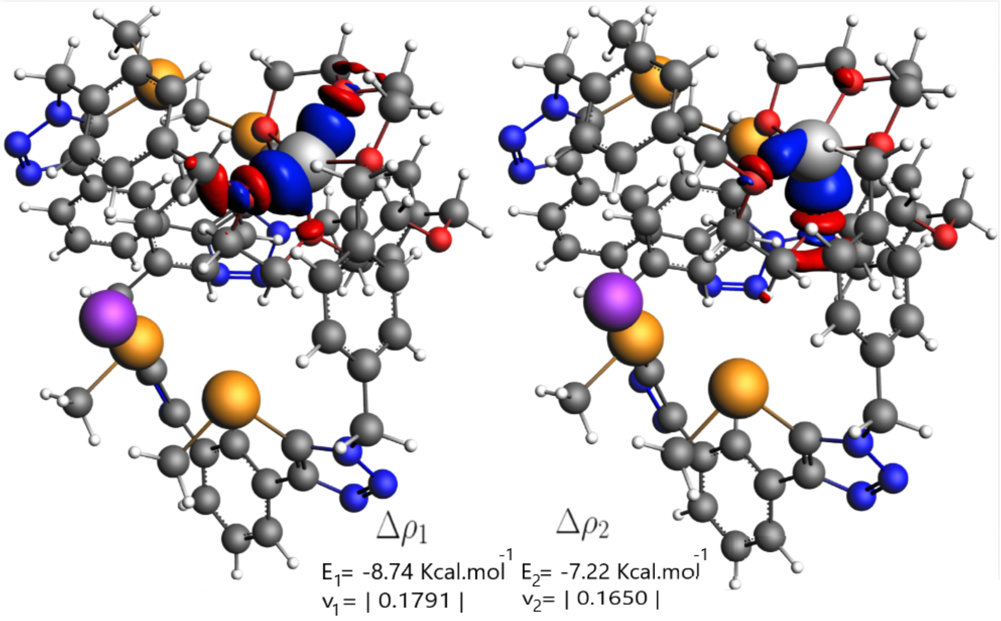

TeO-I$^-$···Li$^+$

Figure S7: The first two density deformation channel surface plots, $\rho_{1,2}$ with isovalue: 0.001 a.u., where the red and blue regions indicate the outflow and inflow electron density for the cation interaction of the **IS** and **TeO**structures

Cartesian Coordinates

Table S1: Cartesian Coordenates of **IO**-Li^+Cl^-

**IO**-$Li^+.Cl^-$	x	y	z
C	9.903226000	17.418544000	23.981717000
C	11.119294000	18.067722000	23.681576000
H	11.683978000	17.802477000	22.782487000
I	12.700210000	15.870110000	31.420234000
C	13.256926000	15.104293000	29.516998000
C	9.187631000	17.752494000	25.151245000
H	8.251203000	17.241631000	25.395957000
N	8.327759000	17.942145000	27.962562000
N	7.828814000	18.384582000	29.146784000
N	14.187253000	13.964758000	27.771697000
N	13.036370000	14.508706000	27.304899000
N	12.459831000	15.203776000	28.389689000
C	14.368018000	14.322260000	29.120283000
C	11.399676000	16.205359000	28.122807000
H	10.988693000	15.961726000	27.129567000
H	10.600519000	16.108397000	28.874136000
N	14.760220000	20.924617000	23.972971000
C	13.492083000	20.936567000	24.523718000
N	13.911013000	18.925504000	23.555434000
N	15.025120000	19.679056000	23.377001000
C	11.630485000	19.067019000	24.555048000
C	12.941305000	19.664107000	24.251697000
C	15.774916000	21.990976000	23.915092000

H	15.282880000	22.925482000	23.597696000
H	16.471885000	21.682417000	23.117131000
I	12.692377000	22.642015000	25.417786000
N	8.173810000	19.744341000	29.238883000
C	8.887330000	20.137148000	28.122492000
I	9.516505000	22.101831000	27.826270000
H	9.247292000	22.782185000	31.191482000
C	9.692250000	21.877536000	31.619744000
H	10.752895000	18.361423000	29.726316000
C	11.616730000	18.560439000	29.086835000
C	8.990235000	18.990002000	27.305712000
C	12.051343000	17.581753000	28.169785000
C	10.909220000	19.401344000	25.727996000
H	11.339499000	20.105691000	26.443416000
C	9.099150000	20.616426000	31.364785000
O	15.997111000	20.470436000	31.353914000
C	15.579095000	19.248818000	32.073663000
H	15.727661000	18.359236000	31.430841000
C	9.688915000	18.747818000	26.033541000
C	12.336560000	19.766716000	29.238432000
H	12.023273000	20.506088000	29.980692000
C	12.699263000	20.888483000	33.811088000
O	13.919551000	20.767405000	32.967445000
H	12.732824000	20.086995000	34.572269000
H	12.806227000	21.871820000	34.297665000
C	16.524031000	22.193575000	25.225786000
C	13.920988000	19.030303000	27.527803000

H	14.826075000	19.203327000	26.934150000
C	13.510501000	19.993902000	28.484930000
C	7.867063000	20.496570000	30.475554000
H	7.477215000	21.487560000	30.191960000
H	7.063934000	19.928692000	30.972576000
C	14.100215000	19.410580000	32.388462000
H	13.780280000	18.643775000	33.119101000
H	13.484615000	19.323097000	31.476927000
C	10.851170000	21.969247000	32.423836000
H	11.305067000	22.948110000	32.620672000
C	11.428931000	20.804329000	32.988366000
C	9.671386000	19.451990000	31.935964000
H	9.212065000	18.473857000	31.751578000
C	13.196764000	17.835032000	27.372236000
H	13.533816000	17.081525000	26.651643000
C	10.822129000	19.543719000	32.744593000
H	11.253242000	18.639232000	33.195540000
O	14.416467000	24.132407000	31.352391000
C	13.972308000	24.360658000	29.962039000
H	13.388344000	25.299750000	29.896613000
H	14.855315000	24.424078000	29.294840000
C	16.718358000	23.500704000	25.731659000
H	16.251604000	24.352986000	25.224568000
C	13.126738000	23.140193000	29.623824000
O	13.957774000	21.957041000	29.919379000
H	12.807829000	23.151499000	28.566688000
H	12.235380000	23.083458000	30.271417000

C	14.405016000	21.189642000	28.749762000
H	14.478403000	21.854047000	27.866222000
H	15.417732000	20.835668000	28.996014000
C	18.010870000	15.271827000	32.502240000
N	19.401094000	15.457115000	32.570481000
C	17.404378000	14.428257000	31.453192000
C	18.099595000	13.266503000	31.017022000
H	19.057296000	13.009586000	31.480755000
C	15.607276000	13.945504000	29.827159000
C	17.418919000	15.975042000	33.579019000
I	15.386566000	16.163528000	34.158119000
N	18.474163000	16.558591000	34.252534000
C	18.466465000	17.390955000	35.474395000
H	17.908933000	16.848187000	36.255894000
H	19.523131000	17.462415000	35.781205000
N	19.704245000	16.245028000	33.633618000
C	16.312892000	12.781660000	29.411462000
H	15.887190000	12.152768000	28.623475000
H	18.087925000	11.555430000	29.675124000
C	17.551401000	12.452232000	30.002177000
C	16.163978000	14.762336000	30.848204000
H	15.655679000	15.684568000	31.141199000
H	16.188916000	19.145643000	32.987687000
C	17.939240000	20.997788000	34.216537000
C	16.710550000	21.342806000	34.834888000
H	18.446034000	21.729970000	33.578932000
C	15.567102000	24.959698000	31.754113000

H	16.437794000	24.719781000	31.110746000
H	15.329322000	26.037079000	31.658550000
C	15.830706000	24.611477000	33.206453000
O	16.041536000	23.148106000	33.279099000
H	14.958088000	24.889684000	33.831231000
H	16.729603000	25.141923000	33.577390000
C	16.123729000	22.726262000	34.702775000
H	15.111248000	22.768195000	35.148472000
H	16.766193000	23.462912000	35.229304000
C	16.064528000	20.390766000	35.659129000
H	15.108647000	20.643453000	36.131387000
C	18.501965000	19.717985000	34.410937000
H	19.445295000	19.452485000	33.919433000
C	17.860491000	18.770279000	35.250110000
C	16.641344000	19.121209000	35.877636000
H	16.134827000	18.394879000	36.520887000
C	17.723774000	21.847709000	30.447018000
O	19.008672000	21.812626000	29.729744000
H	17.813302000	22.478860000	31.351917000
C	17.103886000	21.093614000	25.909132000
H	16.948316000	20.075962000	25.532449000
C	17.520492000	23.712436000	26.877674000
H	17.672502000	24.730476000	27.256156000
C	17.362542000	20.409063000	30.817325000
H	18.053652000	19.997877000	31.575972000
H	17.384188000	19.758166000	29.920623000
C	18.144932000	22.621819000	27.530157000

C	19.099488000	22.858573000	28.694831000
H	18.928316000	23.861254000	29.138175000
H	20.148454000	22.805780000	28.352652000
C	17.907291000	21.307352000	27.048168000
H	18.367405000	20.458750000	27.565193000
H	16.931817000	22.265268000	29.794281000
H	9.515032000	16.649706000	23.306182000
Li	14.871710000	22.068171000	31.813187000
Cl	12.352145000	16.749539000	34.428783000

Table S2: Cartesian Coordenates of **IO**-$Li^{+}.Br^{-}$

**IO**-$Li^{+}.Br^{-}$	x	y	z
C	9.89536787565836	17.22880906580564	24.14866683460592
C	11.14164530902565	17.80997756241525	23.83083727470921
H	11.72064787491857	17.45325044575895	22.97341320335450
I	12.80224590164911	15.78763476664002	31.34982862525978
C	13.40656926825119	15.08337340114575	29.43244999290343
C	9.16345731492736	17.68133986624153	25.26658249064603
H	8.20503793047782	17.22218924243877	25.52697385722821
N	8.18240997737236	18.12092032131212	27.97835204071197
N	7.67731597462535	18.64493161185048	29.12448306037970
N	14.36488992179916	14.01222699018922	27.65937555691782
N	13.21823561065315	14.56973123562689	27.19685526853368
N	12.62603253677523	15.22207534988838	28.29867695469141
C	14.52471781106004	14.31935679295714	29.02278736833967
C	11.54771461775516	16.20737186031162	28.06591318524512
H	11.16607811622546	16.01212892912706	27.05001454871605

H	10.73260906638846	16.04469721837665	28.78901671608344
N	14.84597671026253	20.59746128568587	24.00002672964002
C	13.54538357287999	20.69217056422887	24.45871541330133
N	13.99430888453655	18.56494061632620	23.80074236623202
N	15.12970851969276	19.28093270911279	23.59590965469810
C	11.66028438064991	18.86259846251865	24.63282554375733
C	12.99554414060369	19.39830741130821	24.32531549398813
C	15.88413417874448	21.63734528194245	23.87960167469096
H	15.42332502408323	22.53970831189886	23.44540519670557
H	16.61110036616790	21.23652724787780	23.15319729168273
I	12.70080274390149	22.50317306982396	25.05521462914646
N	8.13235635740208	19.97296590907344	29.18626079255199
C	8.91978210473830	20.26677766833621	28.08849464058361
I	9.73662715253537	22.16305658145519	27.78448825670628
H	9.31599579259416	22.98399013831178	31.18214326223491
C	9.70188124682167	22.05331609276018	31.61217165097162
H	10.68670637192493	18.28109370448886	29.66593823542479
C	11.57759129009826	18.54563033814335	29.08830457672357
C	8.95624170605188	19.08509752172771	27.31411272693710
C	12.14120472961379	17.60355736086482	28.20251389447788
C	10.92360680875076	19.31579953057186	25.75474222665625
H	11.36386978985211	20.06487726577220	26.41619352791331
C	9.05989187511048	20.82608000589109	31.31541467306393
O	15.78021151095245	20.54491942364439	31.23675333991299
C	15.42293058956001	19.30280731160029	31.95889128436060
H	15.54054604715413	18.42707938143921	31.29237767728073
C	9.67674052334475	18.72774695692352	26.08204350531860

C	12.18040636078296	19.81008776627886	29.26429776591526
H	11.75246000894212	20.53651719953948	29.95846029013932
C	12.59974916874958	20.88763647555627	33.85421009025293
O	13.82443123515567	20.77513918293668	33.01289053934570
H	12.60722601368572	20.05207381902327	34.57876988837903
H	12.72660439000612	21.84519281608776	34.38552295864940
C	16.57312844202852	21.96077884819724	25.19893590198320
C	13.93537757972670	19.18763721182436	27.67908286866464
H	14.85877818910422	19.43186193579253	27.14072468687503
C	13.36611852181761	20.13545662597090	28.56716650200427
C	7.85020173674860	20.78406946053190	30.38924266737790
H	7.55427783862290	21.79602338487517	30.06891317215569
H	6.98663101966012	20.29981662490894	30.87356955052379
C	13.96910930969778	19.44028401028692	32.37409841349529
H	13.70067758866135	18.64710531687653	33.09842062380526
H	13.29686937613690	19.37765051847841	31.50134863302620
C	10.83595284037952	22.07616419198217	32.45403308895308
H	11.33310551771838	23.02759656618082	32.67884380100080
C	11.34203217049052	20.87536624745493	33.01110074484722
C	9.56024819213273	19.62463219075822	31.87739867006132
H	9.06468990873900	18.67235926653093	31.65600514585071
C	13.32540281266572	17.93310141225469	27.49573974480952
H	13.77200656868749	17.20546498020678	26.80866238725255
C	10.68982362619600	19.64849892488737	32.72006277118286
H	11.07289869613016	18.71699465036727	33.15872374309925
O	14.62967857567276	24.13038867148167	31.38622122037943
C	14.23020871495443	24.48459748145762	30.01183442307406

H	13.91380475998625	25.54541061826685	29.96692234975368
H	15.08215995946577	24.31955214489756	29.32169236934043
C	16.62575125326158	23.29452932419366	25.66747231551352
H	16.09743336022559	24.08252932121439	25.11905926351301
C	13.06970040393692	23.54465482103222	29.70966162486321
O	13.50848269276543	22.15847318369737	29.93999036747867
H	12.70090289611309	23.67937849968062	28.67594315679479
H	12.24172793209630	23.71926634829418	30.41677050017466
C	14.04349554499844	21.47755202704545	28.74467751526247
H	13.87313001144308	22.11522713632225	27.85403481893738
H	15.13287832064438	21.34680464371265	28.86270077440865
C	18.14846937011677	15.21890753618392	32.42930634793982
N	19.54008863409810	15.39187300191735	32.50449543060937
C	17.54307353822396	14.38446274701677	31.37344492653129
C	18.23981530733035	13.22795898416061	30.92554194265759
H	19.19579337002319	12.96553214222044	31.38967017936305
C	15.75579134671525	13.92455792590911	29.73231848450054
C	17.55730425885980	15.92764590921245	33.50254854117653
I	15.52285322290724	16.11958977887878	34.09645018895088
N	18.61439660032779	16.50112665601293	34.18163893782518
C	18.61216783522308	17.32334469185211	35.40966456962650
H	18.11120849636437	16.75110754703329	36.20861460812122
H	19.67556543192177	17.44126243884216	35.67721498753161
N	19.84454357214950	16.17704613843740	33.56883403442649
C	16.46229612271399	12.76650511823002	29.30332294845268
H	16.04129340132732	12.14999142015919	28.50307194418338
H	18.23467870182665	11.53472212276259	29.56128018561351

C	17.69672325410734	12.42731071033017	29.89735792769980
C	16.30525043034548	14.72623746155261	30.76850050498632
H	15.79458530823460	15.64423905518344	31.07082935640674
H	16.09213151426277	19.19467506634583	32.83084179847810
C	17.82855078185112	20.88954968565059	34.16710760892725
C	16.64728716221162	21.19166433585494	34.89027436880940
H	18.24162419759242	21.63341749608874	33.47859902694152
C	15.73399554614963	24.93491357789985	31.93165416084985
H	16.66361072710164	24.72463705691765	31.36796791568124
H	15.49937748724314	26.01453365429339	31.85956491020869
C	15.84805789657214	24.52125393343609	33.38757836738741
O	15.99583784785632	23.05041417932504	33.40738073235286
H	14.93228937792925	24.80951334123321	33.94232922982919
H	16.72794075605187	24.99984501874561	33.86077934056839
C	15.98896276521146	22.54775084807599	34.80173167707751
H	14.94548802069547	22.50284161256195	35.16772518525227
H	16.54501366882689	23.27752495436476	35.42740384533361
C	16.11367357794157	20.22343061405876	35.77420288170531
H	15.19163217506000	20.44049766117935	36.32579451934847
C	18.45967939506965	19.63624182385222	34.32266852833088
H	19.36589277408977	19.40436433840000	33.75065482848667
C	17.93392809165978	18.67381483667375	35.22212806393245
C	16.75845002620946	18.98092106231026	35.94851920182191
H	16.33582690152758	18.23784514952640	36.63248780931777
C	17.52889343813809	21.98192204774793	30.44683248667963
O	18.86247347441741	21.99795884539770	29.82076093491753
H	17.56837073549754	22.51689884665459	31.41342164563900

C	17.23673346933859	20.94183881610428	25.93134222937699
H	17.19110496978695	19.90403170278125	25.58125951962061
C	17.36860176068641	23.61517609626522	26.82867379120293
H	17.41406535105922	24.65372798594162	27.17796778717754
C	17.13328435102389	20.52058672925754	30.66260090036288
H	17.83181015927524	20.02182990856637	31.36046888993197
H	17.12281524345314	19.96755525993067	29.70371805622869
C	18.07694550579741	22.60941004370243	27.52970583899635
C	18.96597037326611	22.96702356457656	28.71294803819211
H	18.74065193032655	23.99261940245199	29.06993571266203
H	20.03176656479661	22.92607781474005	28.42584166401872
C	17.98196299852318	21.26416185303441	27.08343442202269
H	18.51121180567442	20.47981320058460	27.63473363780475
H	16.78582255456002	22.47579351351075	29.79023202349322
H	9.49731273034451	16.41939515267815	23.52854677555136
Li	14.71550735142614	22.11551263625749	31.84988909149700
Br	12.32611217685595	16.68104606822567	34.50736367194588

Table S3: Cartesian Coordenates of **IO**-Li^+I^-

**IO**-Li^+I^-	x	y	z
C	9.875079000	17.180634000	24.190443000
C	11.136433000	17.727187000	23.870618000
H	11.715832000	17.337151000	23.028158000
I	12.836707000	15.777651000	31.338444000
C	13.430102000	15.107894000	29.418825000
C	9.143206000	17.675455000	25.290257000
H	8.173554000	17.242288000	25.553571000

N	8.147214000	18.196852000	27.977737000
N	7.643648000	18.754042000	29.108523000
N	14.367376000	14.066313000	27.620358000
N	13.207254000	14.617328000	27.183780000
N	12.627298000	15.251773000	28.300710000
C	14.548840000	14.360423000	28.983587000
C	11.540148000	16.233638000	28.096437000
H	11.145631000	16.048834000	27.083528000
H	10.735588000	16.056600000	28.827905000
N	14.902040000	20.432958000	24.011978000
C	13.599777000	20.568715000	24.455178000
N	14.003660000	18.417455000	23.848154000
N	15.157736000	19.101494000	23.639123000
C	11.670199000	18.788236000	24.651451000
C	13.020240000	19.285860000	24.344595000
C	15.964718000	21.446068000	23.874088000
H	15.534570000	22.336258000	23.386312000
H	16.702130000	20.997415000	23.187267000
I	12.794502000	22.410662000	25.008502000
N	8.127236000	20.072669000	29.148853000
C	8.931182000	20.327798000	28.053188000
I	9.796297000	22.198658000	27.723356000
H	9.378786000	23.072231000	31.120271000
C	9.727818000	22.135681000	31.568659000
H	10.649658000	18.304632000	29.685562000
C	11.547988000	18.571990000	29.120492000
C	8.948126000	19.130601000	27.302146000

C	12.128032000	17.631378000	28.243472000
C	10.932332000	19.285470000	25.753979000
H	11.379867000	20.044030000	26.399333000
C	9.049866000	20.926233000	31.278690000
O	15.729503000	20.545719000	31.271702000
C	15.378530000	19.304390000	31.997698000
H	15.492801000	18.427672000	31.331948000
C	9.670991000	18.731045000	26.084496000
C	12.141194000	19.840444000	29.300283000
H	11.699977000	20.566142000	29.986622000
C	12.548627000	20.909816000	33.874616000
O	13.786020000	20.778445000	33.054259000
H	12.528988000	20.072240000	34.596481000
H	12.684092000	21.863320000	34.411220000
C	16.623868000	21.816789000	25.196208000
C	13.919686000	19.224282000	27.739098000
H	14.848665000	19.472818000	27.212378000
C	13.332302000	20.171735000	28.615330000
C	7.853388000	20.911020000	30.334869000
H	7.596980000	21.927215000	29.994708000
H	6.966754000	20.463415000	30.812659000
C	13.926554000	19.441470000	32.422108000
H	13.667952000	18.652150000	33.153038000
H	13.247411000	19.371777000	31.554986000
C	10.848463000	22.133194000	32.428312000
H	11.374154000	23.070281000	32.647755000
C	11.306437000	20.924577000	33.009431000

C	9.502176000	19.716646000	31.863338000
H	8.979442000	18.778024000	31.647141000
C	13.320094000	17.965390000	27.552217000
H	13.780148000	17.239679000	26.872131000
C	10.619703000	19.716388000	32.722064000
H	10.963482000	18.779520000	33.178164000
O	14.608649000	24.148465000	31.439864000
C	14.220469000	24.513694000	30.065523000
H	13.921970000	25.579773000	30.022597000
H	15.072074000	24.336978000	29.377820000
C	16.675407000	23.167813000	25.612932000
H	16.170765000	23.937380000	25.018061000
C	13.044704000	23.595379000	29.757350000
O	13.455802000	22.199675000	29.982436000
H	12.680441000	23.741271000	28.723436000
H	12.219088000	23.782985000	30.463314000
C	13.991882000	21.522842000	28.785114000
H	13.802900000	22.156231000	27.894870000
H	15.084510000	21.407868000	28.890214000
C	18.216166000	15.280661000	32.328915000
N	19.601673000	15.500576000	32.377040000
C	17.610488000	14.448542000	31.272462000
C	18.320863000	13.313173000	30.794014000
H	19.291619000	13.063797000	31.234001000
C	15.798624000	13.976327000	29.664294000
C	17.627471000	15.945280000	33.430102000
I	15.619125000	16.033885000	34.094116000

N	18.678721000	16.538801000	34.101059000
C	18.672577000	17.334935000	35.346707000
H	18.189881000	16.736166000	36.137292000
H	19.735826000	17.468639000	35.606648000
N	19.903239000	16.271550000	33.453523000
C	16.517608000	12.837962000	29.204555000
H	16.091730000	12.224317000	28.404759000
H	18.318680000	11.638103000	29.407382000
C	17.770995000	12.515328000	29.767112000
C	16.353689000	14.774101000	30.699762000
H	15.832721000	15.677993000	31.026300000
H	16.052941000	19.198582000	32.866140000
C	17.819078000	20.908622000	34.181327000
C	16.629231000	21.171075000	34.906016000
H	18.219145000	21.675712000	33.510867000
C	15.717365000	24.939454000	31.995953000
H	16.647734000	24.725727000	31.434324000
H	15.492010000	26.021522000	31.931597000
C	15.820782000	24.511878000	33.448736000
O	15.952990000	23.039774000	33.452717000
H	14.905879000	24.804358000	34.002555000
H	16.703847000	24.976015000	33.930155000
C	15.947240000	22.516561000	34.839544000
H	14.903067000	22.448474000	35.199190000
H	16.488532000	23.244923000	35.479391000
C	16.110757000	20.173392000	35.765657000
H	15.181783000	20.358767000	36.316884000

C	18.475071000	19.665870000	34.312393000
H	19.388136000	19.465265000	33.739518000
C	17.965674000	18.674282000	35.189245000
C	16.780527000	18.940742000	35.916351000
H	16.370485000	18.174920000	36.582458000
C	17.477815000	21.981846000	30.476539000
O	18.817411000	21.999527000	29.863149000
H	17.510028000	22.514371000	31.444624000
C	17.257915000	20.823299000	25.987075000
H	17.213983000	19.772636000	25.677387000
C	17.385741000	23.528763000	26.782339000
H	17.429784000	24.580149000	27.091158000
C	17.079070000	20.520640000	30.688586000
H	17.781243000	20.018942000	31.380672000
H	17.060909000	19.970156000	29.728566000
C	18.063535000	22.546338000	27.544251000
C	18.921904000	22.946210000	28.737061000
H	18.669292000	23.974216000	29.068405000
H	19.993498000	22.921625000	28.469946000
C	17.972103000	21.185566000	27.147266000
H	18.480344000	20.420044000	27.742989000
H	16.741888000	22.479013000	29.814766000
H	9.465494000	16.364828000	23.586444000
I	12.194264000	16.576257000	34.816692000
Li	14.674682000	22.123764000	31.893354000

Table S4: Cartesian Coordenates of **IO**-$Na^{+}.I^{-}$

**IO**-$Na^{+}I^{-}$	x	y	z
C	9.729266000	17.104351000	24.282666000
C	10.981413000	17.628538000	23.899713000
H	11.532863000	17.196878000	23.059180000
I	12.956813000	15.706232000	31.089957000
C	13.798536000	15.354762000	29.176368000
C	9.035281000	17.653804000	25.379905000
H	8.072662000	17.239449000	25.693639000
N	8.059433000	18.297353000	28.009763000
N	7.565496000	18.887266000	29.125323000
N	14.989663000	14.635078000	27.369933000
N	13.848171000	15.185590000	26.883963000
N	13.114320000	15.620706000	28.005056000
C	15.002471000	14.732559000	28.772213000
C	11.961167000	16.523658000	27.819558000
H	11.651710000	16.415650000	26.766507000
H	11.132422000	16.198377000	28.468596000
N	14.781514000	20.274581000	23.770636000
C	13.521823000	20.448857000	24.312692000
N	13.797825000	18.299489000	23.632090000
N	14.957923000	18.943896000	23.352871000
C	11.545941000	18.723161000	24.611026000
C	12.885777000	19.190688000	24.219887000
C	15.888673000	21.236681000	23.625609000
H	15.495269000	22.164043000	23.177915000
H	16.583336000	20.772096000	22.905422000

I	12.849311000	22.308645000	24.971375000
N	8.085800000	20.192165000	29.147063000
C	8.904795000	20.405798000	28.051946000
I	9.789430000	22.261724000	27.686264000
H	9.437581000	23.029994000	31.415620000
C	9.723347000	22.035999000	31.777879000
H	10.816517000	18.309502000	29.591354000
C	11.680081000	18.734214000	29.069880000
C	8.892172000	19.195083000	27.321601000
C	12.391534000	17.949641000	28.135383000
C	10.844162000	19.277167000	25.712002000
H	11.305831000	20.070906000	26.302112000
C	9.018733000	20.895276000	31.322861000
O	15.640154000	20.191393000	31.388689000
C	15.262790000	19.001515000	32.181690000
H	15.386689000	18.087236000	31.567860000
C	9.591535000	18.742979000	26.107679000
C	12.077893000	20.062500000	29.335181000
H	11.525048000	20.673736000	30.051414000
C	12.354974000	20.447198000	34.094246000
O	13.641526000	20.395486000	33.341073000
H	12.268286000	19.524227000	34.697623000
H	12.470124000	21.317746000	34.761116000
C	16.590485000	21.522283000	24.947092000
C	13.939200000	19.813927000	27.768335000
H	14.822508000	20.226685000	27.268228000
C	13.196152000	20.617070000	28.669351000

C	7.860666000	21.031767000	30.340400000
H	7.723432000	22.076553000	30.019451000
H	6.916143000	20.672908000	30.781607000
C	13.802521000	19.138096000	32.567138000
H	13.502428000	18.277236000	33.194135000
H	13.161809000	19.167796000	31.666235000
C	10.789492000	21.892498000	32.694625000
H	11.331019000	22.779568000	33.045989000
C	11.171172000	20.609853000	33.162538000
C	9.396348000	19.610805000	31.790704000
H	8.854577000	18.724227000	31.442636000
C	13.539439000	18.490293000	27.503055000
H	14.112632000	17.878201000	26.797408000
C	10.463037000	19.469811000	32.700469000
H	10.750396000	18.474444000	33.061896000
O	14.587474000	24.455365000	31.594508000
C	14.006073000	24.843294000	30.296183000
H	13.753311000	25.922575000	30.298116000
H	14.729429000	24.640673000	29.480349000
C	16.803980000	22.855184000	25.370120000
H	16.412431000	23.683657000	24.768908000
C	12.748116000	23.991664000	30.153057000
O	13.084702000	22.556864000	30.146661000
H	12.192132000	24.261928000	29.234153000
H	12.093987000	24.135710000	31.029052000
C	13.560324000	22.073467000	28.834992000
H	13.065699000	22.674537000	28.042467000

H	14.653004000	22.225835000	28.735906000
C	18.235232000	15.347346000	32.588486000
N	19.599800000	15.601277000	32.799980000
C	17.778514000	14.613368000	31.394907000
C	18.584607000	13.565887000	30.870655000
H	19.510217000	13.291583000	31.386425000
C	16.183558000	14.299183000	29.539277000
C	17.510747000	15.912731000	33.664037000
I	15.451150000	15.867436000	34.162219000
N	18.465893000	16.487391000	34.479418000
C	18.280207000	17.284380000	35.703905000
H	17.601792000	16.738618000	36.379952000
H	19.275639000	17.340711000	36.175778000
N	19.759580000	16.303445000	33.951116000
C	16.997375000	13.248986000	29.030922000
H	16.692853000	12.731733000	28.115791000
H	18.810172000	12.079814000	29.299251000
C	18.189150000	12.889541000	29.696173000
C	16.581665000	14.972991000	30.723578000
H	15.990515000	15.814698000	31.095371000
H	15.917976000	18.934299000	33.070288000
C	17.954155000	20.847618000	34.278666000
C	16.807470000	21.330157000	34.962107000
H	18.491059000	21.511241000	33.592017000
C	15.938808000	24.988071000	31.839003000
H	16.653830000	24.531933000	31.124634000
H	15.952196000	26.088752000	31.712280000

C	16.284408000	24.638506000	33.275695000
O	16.238562000	23.165815000	33.417023000
H	15.550547000	25.101454000	33.966198000
H	17.299515000	25.006609000	33.524823000
C	16.389799000	22.776879000	34.845214000
H	15.433875000	22.960817000	35.374117000
H	17.165784000	23.432301000	35.292192000
C	16.106148000	20.458167000	35.828546000
H	15.201191000	20.809752000	36.336363000
C	18.411503000	19.530895000	34.492494000
H	19.304249000	19.168779000	33.969614000
C	17.737370000	18.676655000	35.404114000
C	16.569440000	19.141778000	36.051972000
H	16.022966000	18.475501000	36.728070000
C	17.326780000	21.447788000	30.246350000
O	18.667668000	21.351094000	29.649463000
H	17.331320000	22.207746000	31.054196000
C	17.075023000	20.454935000	25.746281000
H	16.898945000	19.418629000	25.435193000
C	17.519087000	23.120692000	26.561809000
H	17.680531000	24.157368000	26.881239000
C	16.982861000	20.067940000	30.802211000
H	17.709957000	19.758177000	31.577407000
H	16.970164000	19.310445000	29.994169000
C	18.037829000	22.058117000	27.341087000
C	18.881918000	22.340323000	28.578106000
H	18.692957000	23.368187000	28.950633000

H	19.957527000	22.250383000	28.343365000
C	17.793811000	20.721301000	26.929349000
H	18.175457000	19.896360000	27.539990000
I	11.930631000	16.064308000	34.525018000
H	16.579299000	21.731295000	29.479340000
H	9.297995000	16.263969000	23.729640000
Na	14.530282000	22.107503000	32.059075000

Table S5: Cartesian Coordenates of **IO**-K^+.I^-

**IO**-K^+.I^-	x	y	z
C	9.871074000	17.191427000	24.136494000
C	11.092945000	17.799378000	23.779152000
H	11.665363000	17.437690000	22.919623000
I	12.843249000	15.551512000	31.081753000
C	13.790255000	15.358674000	29.195556000
C	9.149375000	17.652262000	25.256521000
H	8.209773000	17.174345000	25.548828000
N	8.144864000	18.128340000	27.916760000
N	7.620759000	18.645667000	29.054244000
N	15.086197000	14.750336000	27.419974000
N	13.975117000	15.336766000	26.904422000
N	13.177035000	15.704368000	28.006431000
C	15.014890000	14.757334000	28.822943000
C	12.026680000	16.607701000	27.804140000
H	11.740853000	16.510485000	26.743381000
H	11.184929000	16.269272000	28.429282000
N	14.740075000	20.665075000	23.806182000

C	13.458746000	20.743170000	24.319049000
N	13.888824000	18.635196000	23.590655000
N	15.012183000	19.360050000	23.359202000
C	11.597433000	18.888628000	24.541066000
C	12.909027000	19.449530000	24.179603000
C	15.807374000	21.682246000	23.781217000
H	15.371540000	22.643945000	23.466048000
H	16.514602000	21.349018000	23.003306000
I	12.640173000	22.539672000	24.986353000
N	8.072024000	19.974391000	29.130449000
C	8.876078000	20.276048000	28.045065000
I	9.639935000	22.195630000	27.743262000
H	9.314320000	22.699962000	31.620282000
C	9.601881000	21.687778000	31.926329000
H	10.877820000	18.339737000	29.627761000
C	11.719991000	18.792531000	29.095352000
C	8.927082000	19.096586000	27.266526000
C	12.434463000	18.036113000	28.140181000
C	10.869539000	19.351561000	25.666901000
H	11.291310000	20.140450000	26.292862000
C	8.938439000	20.569101000	31.367012000
O	15.513189000	19.871877000	31.542241000
C	15.114463000	18.695450000	32.335658000
H	15.243110000	17.777625000	31.728620000
C	9.647755000	18.734499000	26.035190000
C	12.077283000	20.134883000	29.359554000
H	11.509442000	20.723451000	30.083714000

C	12.135792000	19.976682000	34.278089000
O	13.472077000	19.978264000	33.613882000
H	12.011126000	19.015318000	34.812015000
H	12.202557000	20.801353000	35.007573000
C	16.502434000	21.805723000	25.132036000
C	13.907999000	19.954376000	27.748466000
H	14.761727000	20.400573000	27.225990000
C	13.158306000	20.730951000	28.669668000
C	7.808005000	20.748636000	30.358971000
H	7.669809000	21.807178000	30.088607000
H	6.855550000	20.362066000	30.758070000
C	13.648199000	18.824441000	32.698573000
H	13.312103000	17.900764000	33.206613000
H	13.040065000	18.968646000	31.785203000
C	10.624886000	21.500636000	32.885088000
H	11.112081000	22.375778000	33.334008000
C	11.008276000	20.195580000	33.287919000
C	9.319218000	19.263083000	31.767622000
H	8.807726000	18.392766000	31.342011000
C	13.549459000	18.619084000	27.485728000
H	14.124246000	18.032688000	26.759818000
C	10.345244000	19.079229000	32.714648000
H	10.631539000	18.066963000	33.025926000
O	14.749802000	24.639945000	31.304680000
C	14.090979000	24.948197000	30.021655000
H	13.916555000	26.040563000	29.938154000
H	14.740342000	24.629767000	29.180839000

C	16.633968000	23.062716000	25.767197000
H	16.188343000	23.952397000	25.307846000
C	12.753791000	24.207882000	30.003982000
O	12.910733000	22.743014000	30.048146000
H	12.180422000	24.499908000	29.100456000
H	12.164239000	24.460899000	30.901647000
C	13.495416000	22.197929000	28.810846000
H	13.078574000	22.759075000	27.947168000
H	14.594624000	22.345185000	28.799556000
C	18.079272000	15.320585000	32.759626000
N	19.428042000	15.641941000	32.975827000
C	17.662029000	14.563932000	31.564971000
C	18.476503000	13.508590000	31.072175000
H	19.377492000	13.220935000	31.623310000
C	16.147724000	14.281684000	29.636863000
C	17.315597000	15.890171000	33.805719000
I	15.259396000	15.697415000	34.287527000
N	18.232536000	16.544747000	34.606541000
C	18.002091000	17.437240000	35.755327000
H	17.168899000	17.032317000	36.351164000
H	18.920206000	17.394081000	36.365472000
N	19.540897000	16.395105000	34.099621000
C	16.969567000	13.224654000	29.158093000
H	16.703515000	12.719389000	28.224235000
H	18.750151000	12.026615000	29.504245000
C	18.123067000	12.842873000	29.877521000
C	16.499241000	14.937763000	30.844567000

H	15.902589000	15.784243000	31.194250000
H	15.752419000	18.625542000	33.236584000
C	18.416366000	20.901846000	34.125307000
C	17.319683000	21.620977000	34.671933000
H	19.127422000	21.418299000	33.469997000
C	16.178358000	25.005006000	31.317970000
H	16.728886000	24.377876000	30.586381000
H	16.302833000	26.071779000	31.041408000
C	16.712392000	24.795699000	32.724711000
O	16.658891000	23.354534000	33.051818000
H	16.106188000	25.368524000	33.456674000
H	17.762622000	25.149106000	32.773865000
C	17.183661000	23.106044000	34.426486000
H	16.491340000	23.568884000	35.158234000
H	18.170702000	23.604707000	34.507552000
C	16.398018000	20.933923000	35.500028000
H	15.534655000	21.469346000	35.911982000
C	18.614471000	19.543146000	34.443564000
H	19.475824000	19.002957000	34.035157000
C	17.718708000	18.873002000	35.318945000
C	16.593888000	19.569349000	35.820349000
H	15.879628000	19.053052000	36.470996000
C	17.181216000	21.103759000	30.375148000
O	18.526518000	21.029458000	29.783899000
H	17.166445000	21.888399000	31.157984000
C	17.055829000	20.654200000	25.750285000
H	16.938572000	19.673306000	25.275266000

C	17.342301000	23.174875000	26.988606000
H	17.445384000	24.154413000	27.471203000
C	16.863571000	19.737531000	30.975703000
H	17.588330000	19.471296000	31.769185000
H	16.869311000	18.948715000	30.197214000
C	17.932604000	22.034346000	27.584284000
C	18.768251000	22.147139000	28.852732000
H	18.585479000	23.119099000	29.357419000
H	19.844994000	22.073943000	28.618185000
C	17.764394000	20.768794000	26.962079000
H	18.192666000	19.879701000	27.436786000
I	11.718454000	15.625796000	34.505576000
H	16.430429000	21.341709000	29.594786000
H	9.484252000	16.355478000	23.545180000
K	14.339315000	22.124455000	32.278391000

Table S6: Cartesian Coordenates of **IO**-Cu^+I^-

**IO**-Cu^+I^-	x	y	z
C	9.800893000	17.135267000	24.335659000
C	11.075751000	17.633762000	23.990065000
H	11.648269000	17.183146000	23.173452000
I	12.895066000	15.728041000	31.279631000
C	13.534809000	15.127287000	29.352777000
C	9.078321000	17.706511000	25.403929000
H	8.099369000	17.309169000	25.688525000
N	8.058887000	18.399716000	28.031316000
N	7.581348000	19.012608000	29.143430000

N	14.515115000	14.150309000	27.541018000
N	13.357873000	14.704641000	27.099906000
N	12.752360000	15.299355000	28.225005000
C	14.667888000	14.403336000	28.915706000
C	11.653669000	16.269095000	28.035701000
H	11.270008000	16.104154000	27.014922000
H	10.846587000	16.061521000	28.756265000
N	14.902027000	20.273097000	24.044766000
C	13.586759000	20.458016000	24.427161000
N	13.977574000	18.262667000	23.995396000
N	15.149662000	18.914468000	23.779849000
C	11.629985000	18.725270000	24.711240000
C	12.992524000	19.178172000	24.391851000
C	15.988577000	21.258556000	23.883295000
H	15.583822000	22.131254000	23.345146000
H	16.732523000	20.764978000	23.236067000
I	12.782716000	22.345367000	24.806722000
N	8.137984000	20.302575000	29.153880000
C	8.960967000	20.484582000	28.057212000
I	9.908061000	22.307148000	27.681942000
H	9.620950000	23.180916000	31.197657000
C	9.889926000	22.206917000	31.621237000
H	10.694650000	18.303990000	29.638239000
C	11.592980000	18.597547000	29.085546000
C	8.915749000	19.268713000	27.337254000
C	12.212203000	17.675145000	28.215032000
C	10.902184000	19.298244000	25.783102000

H	11.367541000	20.079318000	26.387544000
C	9.126671000	21.062529000	31.284180000
O	15.648415000	20.398258000	31.170434000
C	15.359871000	19.164264000	31.940424000
H	15.469326000	18.276439000	31.289161000
C	9.628550000	18.791663000	26.141725000
C	12.136626000	19.888893000	29.258629000
H	11.661850000	20.604708000	29.932519000
C	12.558070000	20.706107000	33.937122000
O	13.812312000	20.587096000	33.124865000
H	12.500356000	19.816406000	34.590268000
H	12.726297000	21.610727000	34.543086000
C	16.621787000	21.679464000	25.203188000
C	13.946089000	19.331327000	27.710930000
H	14.864160000	19.613577000	27.182448000
C	13.310166000	20.266099000	28.565823000
C	7.938564000	21.167505000	30.334670000
H	7.786397000	22.203575000	29.992392000
H	7.009506000	20.811993000	30.809406000
C	13.926806000	19.277319000	32.423374000
H	13.696717000	18.461234000	33.132714000
H	13.212804000	19.247162000	31.581982000
C	10.990911000	22.092346000	32.498947000
H	11.582370000	22.978599000	32.758621000
C	11.342956000	20.834208000	33.049438000
C	9.479867000	19.802165000	31.830069000
H	8.893756000	18.913381000	31.570215000

C	13.401081000	18.045803000	27.537910000
H	13.894645000	17.331840000	26.869231000
C	10.578386000	19.689238000	32.705587000
H	10.846666000	18.714285000	33.131367000
O	14.540927000	24.215205000	31.474796000 a
C	14.230277000	24.583566000	30.076686000
H	14.001817000	25.665634000	30.021440000
H	15.099587000	24.350468000	29.430148000
C	16.599490000	23.034674000	25.609025000
H	16.065592000	23.773256000	25.000885000
C	13.010185000	23.749351000	29.709835000
O	13.307785000	22.317704000	29.881234000
H	12.686353000	23.961490000	28.673521000
H	12.180452000	23.962851000	30.403409000
C	13.881850000	21.659794000	28.688381000
H	13.624171000	22.260572000	27.792025000
H	14.983571000	21.628581000	28.769061000
C	18.260838000	15.287337000	32.343107000
N	19.642245000	15.525115000	32.414881000
C	17.682214000	14.466222000	31.263604000
C	18.410841000	13.345747000	30.777825000
H	19.375018000	13.095450000	31.231551000
C	15.906711000	14.011848000	29.611160000
C	17.646475000	15.931081000	33.442626000
I	15.630345000	15.979549000	34.086044000
N	18.679315000	16.531453000	34.135402000
C	18.639434000	17.323587000	35.381903000

H	18.143007000	16.720218000	36.160397000
H	19.695362000	17.464340000	35.667014000
N	19.916919000	16.287918000	33.504459000
C	16.643344000	12.888461000	29.143233000
H	16.238510000	12.287759000	28.322901000
H	18.449283000	11.698031000	29.359288000
C	17.887640000	12.563908000	29.724920000
C	16.434401000	14.793007000	30.672980000
H	15.900354000	15.686875000	31.006334000
H	16.069508000	19.095035000	32.784939000
C	17.784769000	20.890287000	34.194944000
C	16.603072000	21.161811000	34.929441000
H	18.184656000	21.654373000	33.521053000
C	15.679074000	24.958654000	32.051843000
H	16.601803000	24.718319000	31.489554000
H	15.480942000	26.045757000	31.990367000
C	15.777522000	24.526867000	33.502817000
O	16.008627000	23.067869000	33.505172000
H	14.835487000	24.758909000	34.039278000
H	16.619888000	25.046316000	34.000728000
C	15.935270000	22.514942000	34.877242000
H	14.875469000	22.446909000	35.189115000
H	16.454496000	23.221948000	35.558472000
C	16.080092000	20.166024000	35.788778000
H	15.152735000	20.356197000	36.341291000
C	18.435874000	19.644483000	34.326484000
H	19.345756000	19.438913000	33.750339000

C	17.928045000	18.659055000	35.210743000
C	16.743487000	18.929981000	35.937545000
H	16.331285000	18.165773000	36.604379000
C	17.367112000	21.932499000	30.480872000
O	18.724174000	22.024888000	29.908197000
H	17.351221000	22.431755000	31.466552000
C	17.299150000	20.726572000	26.008272000
H	17.311921000	19.672815000	25.706476000
C	17.274321000	23.440027000	26.784934000
H	17.260481000	24.494674000	27.085354000
C	17.021768000	20.450474000	30.633851000
H	17.718564000	19.956868000	31.336943000
H	17.052799000	19.927492000	29.660318000
C	17.993199000	22.499968000	27.562705000
C	18.810363000	22.953111000	28.765175000
H	18.509769000	23.975766000	29.071793000
H	19.887400000	22.968163000	28.519826000
C	17.981293000	21.134186000	27.172653000
H	18.525340000	20.400835000	27.777130000
H	16.629513000	22.422864000	29.816734000
H	9.374352000	16.296298000	23.776837000
I	12.174741000	16.445614000	34.755923000
Cu	14.524550000	22.114896000	31.881441000

Table S7: Cartesian Coordenates of **IO**-Ag^{+}.I^{-}

**IO**-Ag^{+}.I^{-}	x	y	z
C	9.736675000	17.075504000	24.408448000

C	10.996202000	17.569305000	24.005953000
H	11.543631000	17.096450000	23.184754000
I	12.964693000	15.681832000	31.215550000
C	13.746459000	15.287242000	29.285289000
C	9.047448000	17.675355000	25.482494000
H	8.080762000	17.282177000	25.811055000
N	8.107329000	18.427844000	28.110968000
N	7.653585000	19.061405000	29.220023000
N	14.862383000	14.501327000	27.458440000
N	13.725497000	15.080618000	26.995559000
N	13.038548000	15.557967000	28.129534000
C	14.918455000	14.621295000	28.857775000
C	11.912594000	16.496372000	27.961166000
H	11.551311000	16.362677000	26.927501000
H	11.103149000	16.225759000	28.657598000
N	14.810718000	20.219046000	23.845189000
C	13.511670000	20.413127000	24.275226000
N	13.882725000	18.209927000	23.865716000
N	15.046283000	18.855396000	23.594540000
C	11.568089000	18.685912000	24.673139000
C	12.915278000	19.133683000	24.287029000
C	15.906936000	21.192859000	23.677978000
H	15.511488000	22.075215000	23.148955000
H	16.642031000	20.694594000	23.024215000
I	12.722619000	22.307722000	24.644930000
N	8.198184000	20.356791000	29.187733000
C	8.989156000	20.521400000	28.063954000

I	9.883151000	22.351236000	27.603317000
H	9.798075000	23.139811000	31.334863000
C	10.013084000	22.140394000	31.728923000
H	10.870088000	18.404094000	29.664392000
C	11.729233000	18.777253000	29.097779000
C	8.936835000	19.288440000	27.373792000
C	12.400382000	17.919882000	28.198301000
C	10.873166000	19.288710000	25.751667000
H	11.349394000	20.094863000	26.313129000
C	9.212090000	21.042264000	31.332152000
O	15.610634000	20.033674000	31.209158000
C	15.340510000	18.882919000	32.096245000
H	15.446992000	17.939383000	31.527285000
C	9.615808000	18.784924000	26.169006000
C	12.149933000	20.115964000	29.255632000
H	11.621715000	20.784680000	29.937952000
C	12.530859000	20.442720000	34.087772000
O	13.812355000	20.303218000	33.329668000
H	12.399570000	19.532696000	34.701342000
H	12.706643000	21.315626000	34.737345000
C	16.540699000	21.588223000	25.005937000
C	13.963151000	19.729117000	27.660682000
H	14.835139000	20.096042000	27.108070000
C	13.250187000	20.607782000	28.513983000
C	8.042469000	21.227879000	30.370439000
H	7.956098000	22.272533000	30.032668000
H	7.089757000	20.929899000	30.838297000

C	13.912997000	19.017327000	32.587415000
H	13.668369000	18.184352000	33.271681000
H	13.199395000	19.017928000	31.743135000
C	11.080363000	21.949229000	32.636410000
H	11.688119000	22.806216000	32.954655000
C	11.361877000	20.659046000	33.154364000
C	9.499299000	19.749369000	31.841254000
H	8.886257000	18.895682000	31.531954000
C	13.542185000	18.395059000	27.505263000
H	14.085262000	17.727072000	26.827559000
C	10.565083000	19.559479000	32.742468000
H	10.781579000	18.559890000	33.139648000
O	14.556270000	24.458925000	31.466711000
C	14.285519000	24.830338000	30.064490000
H	14.187648000	25.930987000	29.980734000
H	15.116663000	24.486719000	29.416223000
C	16.580617000	22.943524000	25.410752000
H	16.114138000	23.710487000	24.782400000
C	12.969023000	24.149562000	29.706757000
O	13.091267000	22.681060000	29.777238000
H	12.632709000	24.453051000	28.697028000
H	12.194944000	24.413048000	30.446114000
C	13.627783000	22.069352000	28.543183000
H	13.172452000	22.594515000	27.677877000
H	14.724517000	22.207668000	28.482895000
C	18.289929000	15.285899000	32.535887000
N	19.659828000	15.559852000	32.670055000

C	17.778353000	14.518718000	31.385700000
C	18.547930000	13.442752000	30.864655000
H	19.488228000	13.169099000	31.353583000
C	16.112304000	14.177365000	29.598299000
C	17.614438000	15.870798000	33.632578000
I	15.578188000	15.817241000	34.211073000
N	18.602044000	16.478768000	34.382976000
C	18.482076000	17.280797000	35.616220000
H	17.922985000	16.692822000	36.362966000
H	19.515325000	17.414941000	35.977488000
N	19.868919000	16.293895000	33.793410000
C	16.888987000	13.097977000	29.093557000
H	16.544050000	12.560148000	28.204990000
H	18.690372000	11.905069000	29.333555000
C	18.097676000	12.737279000	29.727260000
C	16.563143000	14.876788000	30.748152000
H	15.998755000	15.738312000	31.115134000
H	16.059971000	18.892153000	32.935161000
C	17.744187000	20.818691000	34.272009000
C	16.542317000	21.139826000	34.950395000
H	18.185201000	21.549312000	33.586517000
C	15.849419000	24.950748000	31.978833000
H	16.676985000	24.533873000	31.371244000
H	15.876315000	26.056712000	31.923440000
C	15.948052000	24.505949000	33.426253000
O	16.061404000	23.031570000	33.450953000
H	15.043225000	24.825151000	33.981744000

H	16.842779000	24.954477000	33.901770000
C	15.905953000	22.505324000	34.828392000
H	14.829829000	22.464871000	35.084528000
H	16.401906000	23.214489000	35.524614000
C	15.965360000	20.183777000	35.821090000
H	15.020374000	20.410998000	36.327189000
C	18.364392000	19.565840000	34.473881000
H	19.292251000	19.322746000	33.942709000
C	17.801712000	18.621571000	35.369805000
C	16.593290000	18.938780000	36.035850000
H	16.137188000	18.204150000	36.707702000
C	17.196818000	21.566285000	30.308384000
O	18.547431000	21.702926000	29.739893000
H	17.087572000	22.238534000	31.182561000
C	17.128297000	20.600269000	25.837678000
H	17.089552000	19.546201000	25.539170000
C	17.221725000	23.310510000	26.617780000
H	17.250187000	24.364187000	26.921069000
C	17.001261000	20.111352000	30.731013000
H	17.698174000	19.828416000	31.542041000
H	17.137865000	19.426683000	29.872581000
C	17.840499000	22.329969000	27.430932000
C	18.607982000	22.730600000	28.685906000
H	18.241753000	23.705455000	29.069734000
H	19.686210000	22.829360000	28.467033000
C	17.773766000	20.968229000	27.035590000
H	18.235844000	20.204991000	27.670153000

H	16.431881000	21.822771000	29.549691000
H	9.296547000	16.218239000	23.889387000
I	12.046745000	16.118185000	34.672061000
Ag	14.329756000	22.016705000	31.781765000

Table S8: Cartesian Coordenates of **IO**-$Au^{+}.I^{-}$

**IO**-$Au^{+}.I^{-}$	x	y	z
C	9.941099000	17.302802000	24.046551000
C	11.132571000	17.994068000	23.739356000
H	11.722170000	17.721783000	22.858392000
I	12.733070000	15.742291000	31.487944000
C	13.376844000	15.129174000	29.568422000
C	9.195277000	17.644155000	25.194900000
H	8.280128000	17.099073000	25.446129000
N	8.285919000	17.895927000	27.987114000
N	7.765747000	18.353978000	29.155624000
N	14.360223000	14.114949000	27.779673000
N	13.206875000	14.665143000	27.324342000
N	12.599334000	15.285357000	28.434575000
C	14.508907000	14.391936000	29.150044000
C	11.510105000	16.261993000	28.211680000
H	11.081493000	16.025919000	27.223747000
H	10.731610000	16.125448000	28.978513000
N	14.656520000	21.004790000	24.042880000
C	13.352100000	20.998295000	24.502062000
N	13.924263000	18.940178000	23.728087000
N	15.015926000	19.730592000	23.567920000

C	11.584446000	19.044668000	24.583708000
C	12.878673000	19.683211000	24.295353000
C	15.648445000	22.093281000	24.016812000
H	15.139016000	23.024878000	23.720698000
H	16.360523000	21.822441000	23.218588000
I	12.398359000	22.719809000	25.192587000
N	8.065667000	19.726641000	29.210667000
C	8.773472000	20.112449000	28.087946000
I	9.338341000	22.089949000	27.746260000
H	9.282564000	22.702823000	31.150435000
C	9.662109000	21.776708000	31.594167000
H	10.734885000	18.397971000	29.778024000
C	11.584205000	18.642585000	29.134291000
C	8.918485000	18.946095000	27.304992000
C	12.103501000	17.664762000	28.259783000
C	10.832317000	19.388163000	25.733774000
H	11.221724000	20.136952000	26.426818000
C	8.995962000	20.552040000	31.340958000
O	15.786275000	19.952526000	31.033254000
C	15.430579000	18.946735000	32.048161000
H	15.571667000	17.922999000	31.649607000
C	9.639281000	18.690998000	26.048179000
C	12.171103000	19.926634000	29.204622000
H	11.763957000	20.684600000	29.877681000
C	12.538417000	20.615768000	33.857197000
O	13.804096000	20.489996000	33.023671000
H	12.567759000	19.764948000	34.559602000

H	12.687520000	21.566286000	34.391633000
C	16.378161000	22.266066000	25.343429000
C	13.817418000	19.254196000	27.525554000
H	14.685472000	19.488976000	26.899645000
C	13.292708000	20.238306000	28.401226000
C	7.767330000	20.491874000	30.440188000
H	7.425174000	21.498157000	30.151179000
H	6.935859000	19.960235000	30.930096000
C	13.966501000	19.141429000	32.379123000
H	13.639708000	18.393261000	33.122588000
H	13.326246000	19.094911000	31.483124000
C	10.808077000	21.804312000	32.420118000
H	11.316940000	22.754715000	32.623173000
C	11.301995000	20.606983000	32.998171000
C	9.490252000	19.355721000	31.919020000
H	8.977243000	18.406636000	31.728130000
C	13.227783000	17.979127000	27.454836000
H	13.633847000	17.228989000	26.766985000
C	10.634229000	19.382509000	32.738431000
H	11.006417000	18.457372000	33.194892000
O	14.849681000	24.125804000	31.073420000
C	14.863054000	24.284991000	29.585022000
H	15.069650000	25.350816000	29.379221000
H	15.680121000	23.666032000	29.175751000
C	16.565964000	23.557167000	25.891305000
H	16.115188000	24.426424000	25.398809000
C	13.501410000	23.924792000	29.006368000

O	13.112788000	22.526119000	29.198366000
H	13.525015000	24.171569000	27.923545000
H	12.707669000	24.514187000	29.494183000
C	13.994640000	21.571144000	28.497958000
H	14.247716000	21.961711000	27.492983000
H	14.932870000	21.446391000	29.072700000
C	18.117304000	15.304319000	32.558785000
N	19.499867000	15.542265000	32.610505000
C	17.527020000	14.467898000	31.496996000
C	18.249246000	13.338748000	31.022242000
H	19.214622000	13.090665000	31.474625000
C	15.743902000	14.002733000	29.854769000
C	17.516571000	15.967073000	33.654379000
I	15.509954000	16.020429000	34.330548000
N	18.557870000	16.577861000	34.324958000
C	18.527527000	17.406911000	35.547301000
H	17.964555000	16.859974000	36.321702000
H	19.579126000	17.487606000	35.868917000
N	19.787970000	16.323068000	33.683816000
C	16.474893000	12.870600000	29.398417000
H	16.065939000	12.263382000	28.585008000
H	18.274848000	11.673349000	29.624149000
C	17.718624000	12.546287000	29.981129000
C	16.277324000	14.791516000	30.908154000
H	15.747964000	15.689257000	31.237760000
H	16.060691000	19.078269000	32.947786000
C	18.005910000	20.997815000	34.251742000

C	16.812615000	21.378057000	34.916643000
H	18.503250000	21.712989000	33.589929000
C	15.992754000	24.869383000	31.703820000
H	16.933192000	24.522613000	31.238297000
H	15.827161000	25.937856000	31.481354000
C	16.004656000	24.629920000	33.199363000
O	16.550727000	23.283954000	33.428156000
H	14.977763000	24.714204000	33.612917000
H	16.646371000	25.393714000	33.683753000
C	16.248043000	22.774922000	34.780416000
H	15.150105000	22.770918000	34.938340000
H	16.702050000	23.458805000	35.529802000
C	16.165407000	20.441670000	35.759550000
H	15.228031000	20.715058000	36.257913000
C	18.548261000	19.708326000	34.440984000
H	19.470406000	19.420955000	33.922293000
C	17.917973000	18.782799000	35.310669000
C	16.718780000	19.158467000	35.963224000
H	16.212541000	18.443375000	36.619753000
C	17.489741000	21.533524000	30.484924000
O	18.810569000	21.673885000	29.844251000
H	17.451003000	22.124608000	31.418818000
C	16.941437000	21.145535000	26.006351000
H	16.793871000	20.140258000	25.595230000
C	17.340591000	23.731057000	27.062819000
H	17.489277000	24.737662000	27.472094000
C	17.233772000	20.051359000	30.759703000

H	17.814165000	19.688201000	31.627496000
H	17.482426000	19.439618000	29.871855000
C	17.941342000	22.618646000	27.701220000
C	18.851679000	22.811604000	28.910677000
H	18.602561000	23.758178000	29.435248000
H	19.908862000	22.865353000	28.594314000
C	17.714192000	21.321369000	27.171945000
H	18.156786000	20.455963000	27.675985000
H	16.700821000	21.894123000	29.797950000
H	9.596920000	16.494142000	23.394031000
I	12.090343000	16.447070000	35.003601000
Au	14.328930000	22.206453000	31.898418000

Table S9: Cartesian Coordenates of **IO**-$Ni^{2+}.PF_6{}^{-}I^{-}$

**IO**-$Ni^{2+}.PF_6{}^{-}.I^{-}$	x	y	z
C	9.800638000	17.256746000	24.082870000
C	11.011619000	17.918671000	23.793787000
H	11.595340000	17.647286000	22.909107000
I	12.935571000	15.825188000	31.325600000
C	13.555472000	15.162251000	29.414821000
C	9.057645000	17.602165000	25.231490000
H	8.122714000	17.084380000	25.466729000
N	8.121884000	17.836145000	28.006470000
N	7.586039000	18.291023000	29.168859000
N	14.576176000	14.180765000	27.629544000
N	13.356753000	14.584441000	27.199144000
N	12.722553000	15.182172000	28.306614000

C	14.745391000	14.532257000	28.981602000
C	11.539582000	16.045421000	28.074365000
H	11.178080000	15.801220000	27.062181000
H	10.751269000	15.803442000	28.804304000
N	14.590918000	20.843288000	24.021637000
C	13.351001000	20.827199000	24.633522000
N	13.737675000	18.852376000	23.581165000
N	14.835966000	19.619806000	23.375656000
C	11.494050000	18.942821000	24.656561000
C	12.793780000	19.560523000	24.343366000
C	15.584567000	21.926720000	23.925785000
H	15.075548000	22.841261000	23.577363000
H	16.283674000	21.603489000	23.135211000
I	12.605436000	22.498118000	25.636191000
N	7.914546000	19.656046000	29.248344000
C	8.656010000	20.039051000	28.145919000
I	9.265123000	22.007755000	27.835276000
H	8.897937000	22.723586000	31.269111000
C	9.382520000	21.818933000	31.651075000
H	10.491106000	18.035119000	29.668927000
C	11.375886000	18.367239000	29.119256000
C	8.793105000	18.880184000	27.351191000
C	11.999580000	17.491654000	28.208467000
C	10.744910000	19.287935000	25.809104000
H	11.145877000	20.017300000	26.516292000
C	8.827294000	20.548861000	31.356540000
O	15.867196000	20.595633000	31.373971000

C	15.478121000	19.354570000	32.065881000
H	15.666765000	18.471987000	31.422763000
C	9.527649000	18.622026000	26.102577000
C	11.933805000	19.636555000	29.386063000
H	11.470610000	20.297493000	30.120303000
C	12.437753000	20.852821000	33.758133000
O	13.723392000	20.798311000	32.945590000
H	12.531432000	20.016446000	34.474817000
H	12.505394000	21.817664000	34.282762000
C	16.333298000	22.189476000	25.225029000
C	13.728931000	19.175008000	27.786381000
H	14.652694000	19.482167000	27.283327000
C	13.136526000	20.022928000	28.758009000
C	7.591783000	20.417348000	30.473283000
H	7.195005000	21.403286000	30.182968000
H	6.793291000	19.848179000	30.976251000
C	13.986851000	19.457947000	32.329425000
H	13.664580000	18.691536000	33.060450000
H	13.383661000	19.370777000	31.411766000
C	10.552642000	21.917278000	32.435791000
H	10.972206000	22.901840000	32.676224000
C	11.188142000	20.748375000	32.924863000
C	9.449224000	19.381395000	31.865132000
H	9.017105000	18.397285000	31.653075000
C	13.161408000	17.918284000	27.514964000
H	13.640861000	17.250844000	26.790352000
C	10.620862000	19.479431000	32.639280000

H	11.092050000	18.573085000	33.036726000
O	14.145449000	24.156986000	31.245220000
C	13.668041000	24.307751000	29.832675000
H	13.296371000	25.336416000	29.703098000
H	14.508115000	24.111710000	29.146348000
C	16.591396000	23.522134000	25.624525000
H	16.173263000	24.353008000	25.044312000
C	12.565448000	23.266120000	29.765231000
O	13.165648000	21.980326000	30.271447000
H	12.190771000	23.099446000	28.743346000
H	11.733165000	23.490634000	30.446347000
C	13.915044000	21.238441000	29.192888000
H	14.128404000	21.938842000	28.366465000
H	14.859270000	20.932951000	29.671738000
C	18.349401000	15.827261000	32.289687000
N	19.697617000	16.216577000	32.323736000
C	17.825174000	14.958124000	31.218830000
C	18.655221000	13.924710000	30.704733000
H	19.658635000	13.786134000	31.119508000
C	16.044846000	14.299677000	29.640154000
C	17.709177000	16.368513000	33.427697000
I	15.718315000	16.207741000	34.112272000
N	18.692044000	17.063787000	34.105638000
C	18.604445000	17.825882000	35.370644000
H	18.170531000	17.168128000	36.141835000
H	19.647387000	18.053937000	35.644737000
N	19.925309000	16.976824000	33.425869000

C	16.883879000	13.260195000	29.148914000
H	16.515790000	12.610568000	28.349184000
H	18.818993000	12.281886000	29.289471000
C	18.179839000	13.081994000	29.676724000
C	16.525416000	15.142674000	30.677575000
H	15.909510000	15.973200000	31.033951000
H	16.058314000	19.255780000	32.997614000
C	17.467345000	21.326940000	34.243302000
C	16.198211000	21.430806000	34.866633000
H	17.834586000	22.148979000	33.620480000
C	15.340759000	24.927766000	31.692095000
H	16.225379000	24.566172000	31.147125000
H	15.174563000	25.997087000	31.492858000
C	15.384904000	24.639568000	33.175938000
O	15.291710000	23.138467000	33.307451000
H	14.532987000	25.090800000	33.713170000
H	16.342038000	24.962022000	33.616312000
C	15.380507000	22.686770000	34.745617000
H	14.358668000	22.567997000	35.138708000
H	15.865646000	23.526600000	35.275444000
C	15.730820000	20.370076000	35.678036000
H	14.750544000	20.441884000	36.161930000
C	18.243195000	20.161185000	34.405563000
H	19.214183000	20.073870000	33.905019000
C	17.777578000	19.096512000	35.219991000
C	16.524165000	19.218043000	35.867459000
H	16.157845000	18.402110000	36.497539000

C	17.545897000	22.018937000	30.437925000
O	18.831792000	22.000138000	29.727490000
H	17.609685000	22.689781000	31.316408000
C	16.852527000	21.121456000	26.000029000
H	16.653106000	20.084950000	25.703231000
C	17.391975000	23.789945000	26.758168000
H	17.598274000	24.825391000	27.046590000
C	17.247613000	20.581136000	30.858673000
H	17.941530000	20.228242000	31.642480000
H	17.311441000	19.901762000	29.985855000
C	17.951712000	22.725608000	27.506379000
C	18.900553000	23.020503000	28.662293000
H	18.706435000	24.028399000	29.077687000
H	19.950497000	22.980551000	28.321949000
C	17.654875000	21.389253000	27.129567000
H	18.074046000	20.564074000	27.715259000
H	16.737395000	22.377882000	29.770627000
H	9.436627000	16.470663000	23.413825000
I	12.318742000	16.728959000	34.854631000
F	16.289996000	28.643487000	28.405694000
F	17.846679000	25.979532000	30.081184000
F	18.389185000	28.374266000	29.728985000
F	15.765353000	26.249797000	28.771674000
F	16.201349000	27.732383000	30.710435000
F	17.947983000	26.897295000	27.777906000
P	17.081927000	27.329297000	29.238607000
Ni	14.128669000	22.383421000	31.928398000

Table S10: Cartesian Coordenates of **IO**-$Zn^{2+}.PF_6^-.I^-$

**IO**-$Zn^{2+}.PF_6^-.I^-$	x	y	z
C	9.757423000	16.877405000	24.342004000
C	10.937570000	17.522676000	23.919772000
H	11.513549000	17.133336000	23.075375000
I	13.142188000	16.169871000	31.062988000
C	14.017447000	15.672292000	29.199518000
C	9.031507000	17.375879000	25.442389000
H	8.122907000	16.872611000	25.785226000
N	7.970203000	17.995728000	28.027104000
N	7.398149000	18.562551000	29.115730000
N	15.242111000	14.792577000	27.489453000
N	14.146574000	15.361589000	26.925940000
N	13.391172000	15.896056000	27.987498000
C	15.204863000	14.972085000	28.882352000
C	12.244026000	16.777354000	27.692483000
H	12.002690000	16.614115000	26.628991000
H	11.379876000	16.469658000	28.302932000
N	14.458536000	20.510965000	23.638783000
C	13.221223000	20.576829000	24.252820000
N	13.617932000	18.472737000	23.494281000
N	14.708955000	19.209299000	23.172603000
C	11.398818000	18.687776000	24.594206000
C	12.675081000	19.276399000	24.155704000
C	15.491949000	21.550210000	23.474040000
H	14.995601000	22.501133000	23.222323000
H	16.092610000	21.232038000	22.605483000

I	12.471364000	22.365955000	25.016599000
N	7.802500000	19.908208000	29.120310000
C	8.635005000	20.168264000	28.044295000
I	9.358862000	22.087991000	27.659976000
H	8.520903000	22.985273000	31.550402000
C	9.102986000	22.081959000	31.764875000
H	11.032088000	18.574117000	29.395030000
C	11.891749000	19.008938000	28.877202000
C	8.741895000	18.947760000	27.338061000
C	12.630857000	18.223966000	27.964744000
C	10.662266000	19.192304000	25.696808000
H	11.041649000	20.052334000	26.251458000
C	8.709156000	20.842999000	31.207601000
O	15.726756000	20.816663000	31.238872000
C	15.336430000	19.618288000	32.050874000
H	15.590390000	18.707336000	31.483570000
C	9.482022000	18.536472000	26.132663000
C	12.261514000	20.346133000	29.133568000
H	11.677388000	20.932216000	29.846025000
C	12.304449000	21.100485000	33.653384000
O	13.540174000	21.029699000	32.799740000
H	12.390234000	20.274865000	34.382714000
H	12.379539000	22.068430000	34.166371000
C	16.370848000	21.697728000	24.709409000
C	14.146722000	20.114264000	27.595432000
H	15.028116000	20.537528000	27.101370000
C	13.389043000	20.910812000	28.490395000

C	7.492770000	20.746651000	30.292173000
H	7.165814000	21.742472000	29.952342000
H	6.644915000	20.253283000	30.796361000
C	13.842238000	19.672067000	32.273505000
H	13.544624000	18.911547000	33.017771000
H	13.284124000	19.516481000	31.336386000
C	10.257340000	22.158990000	32.577900000
H	10.564052000	23.121204000	33.002295000
C	11.035999000	21.004387000	32.837861000
C	9.462502000	19.678120000	31.500465000
H	9.150684000	18.708292000	31.097871000
C	13.767762000	18.783295000	27.329725000
H	14.357498000	18.176862000	26.632848000
C	10.610183000	19.756904000	32.309947000
H	11.166539000	18.843789000	32.547809000
O	13.171710000	23.958478000	32.435338000
C	12.472309000	24.656156000	31.325557000
H	11.512710000	25.064072000	31.690379000
H	13.118029000	25.472446000	30.962110000
C	16.522522000	22.955420000	25.337597000
H	15.955539000	23.818301000	24.970540000
C	12.219713000	23.608674000	30.244463000
O	13.473641000	22.840684000	30.057360000
H	11.954435000	24.109383000	29.298192000
H	11.420790000	22.909129000	30.533058000
C	13.784819000	22.356165000	28.691300000
H	13.263341000	23.022687000	27.978726000

H	14.868492000	22.497040000	28.553511000
C	18.357677000	15.604098000	32.768729000
N	19.732741000	15.724468000	33.028988000
C	17.872374000	14.822572000	31.617171000
C	18.597370000	13.670657000	31.205455000
H	19.475594000	13.356770000	31.778234000
C	16.321836000	14.507114000	29.724279000
C	17.659507000	16.345054000	33.750337000
I	15.586450000	16.542314000	34.160846000
N	18.638544000	16.883882000	34.561890000
C	18.492194000	17.841783000	35.670562000
H	17.755702000	17.447837000	36.390048000
H	19.477646000	17.882841000	36.164524000
N	19.923954000	16.511062000	34.118505000
C	17.055769000	13.354954000	29.327705000
H	16.738177000	12.797367000	28.441085000
H	18.743973000	12.055013000	29.757651000
C	18.185143000	12.943987000	30.067698000
C	16.736515000	15.232639000	30.871479000
H	16.209586000	16.148492000	31.154346000
H	15.907365000	19.650941000	32.992738000
C	18.413673000	21.107447000	33.635664000
C	17.336027000	21.804649000	34.236659000
H	18.966904000	21.573846000	32.812624000
C	13.680214000	24.850026000	33.510709000
H	14.207866000	25.700077000	33.045970000
H	12.832682000	25.208562000	34.121575000

C	14.634190000	24.004396000	34.353421000
O	15.549314000	23.317243000	33.412923000
H	14.103941000	23.241741000	34.948986000
H	15.214118000	24.657495000	35.027029000
C	16.992832000	23.199463000	33.766296000
H	17.199893000	23.964151000	34.536234000
H	17.546227000	23.464082000	32.852197000
C	16.608606000	21.180938000	35.282355000
H	15.760751000	21.697126000	35.744662000
C	18.783562000	19.825348000	34.095172000
H	19.620874000	19.295885000	33.626474000
C	18.073180000	19.215792000	35.158799000
C	16.972293000	19.894907000	35.736243000
H	16.400558000	19.414767000	36.537715000
C	17.568405000	21.770436000	29.886188000
O	18.794492000	21.313221000	29.211225000
H	17.827312000	22.478655000	30.693531000
C	17.088521000	20.578306000	25.208376000
H	16.961777000	19.597915000	24.734795000
C	17.412569000	23.105482000	26.428508000
H	17.533840000	24.083527000	26.905798000
C	16.938550000	20.498401000	30.432943000
H	17.653365000	19.973452000	31.091261000
H	16.627401000	19.826952000	29.613254000
C	18.163242000	22.001227000	26.900965000
C	19.155037000	22.155775000	28.040939000
H	19.239352000	23.214469000	28.349499000

H	20.152414000	21.781136000	27.755171000
C	17.974868000	20.730344000	26.292757000
H	18.536997000	19.866838000	26.665738000
H	16.890737000	22.270071000	29.173008000
H	9.406669000	15.983594000	23.816569000
I	12.096786000	16.812932000	34.492893000
F	17.725265000	24.816609000	29.303883000
F	15.146553000	26.352749000	31.095986000
F	17.466106000	25.690784000	31.602759000
F	15.373533000	25.439697000	28.814535000
F	16.985017000	27.168111000	29.636606000
F	15.888488000	24.004601000	30.749207000
P	16.447554000	25.611924000	30.185120000
Zn	14.604572000	22.515805000	31.754011000

Table S11: Cartesian Coordenates of **IS**-Li^+I^-

**IS**-$Li^+.I^-$	x	y	z
C	10.209053000	16.625133000	24.134389000
C	11.304348000	17.394820000	23.688989000
H	11.902951000	17.067653000	22.833459000
I	13.062733000	16.026699000	31.246854000
C	14.059822000	15.620817000	29.419632000
C	9.451307000	17.043772000	25.247788000
H	8.611732000	16.441912000	25.608419000
N	8.531636000	17.551987000	27.956758000
N	7.972815000	18.074569000	29.077178000
N	15.384666000	14.851733000	27.729808000

N	14.313543000	15.438322000	27.141487000
N	13.496430000	15.901439000	28.188835000
C	15.268473000	14.941282000	29.127784000
C	12.295447000	16.695090000	27.870904000
H	12.048118000	16.461921000	26.821914000
H	11.463669000	16.356275000	28.509357000
N	14.406274000	20.828873000	23.357044000
C	13.160181000	20.721953000	23.945723000
N	13.901875000	18.676542000	23.291711000
N	14.871387000	19.564761000	22.955817000
C	11.644808000	18.604608000	24.354387000
C	12.830709000	19.348444000	23.900880000
C	15.273460000	22.012675000	23.183575000
H	14.644981000	22.857823000	22.860398000
H	15.963381000	21.753102000	22.364436000
I	12.075312000	22.385493000	24.582225000
N	8.148381000	19.466163000	28.994766000
C	8.814863000	19.803302000	27.831024000
I	9.188465000	21.788833000	27.308249000
H	8.915879000	22.676783000	30.961430000
C	9.336086000	21.802926000	31.471305000
H	10.983084000	18.462510000	29.521488000
C	11.765182000	18.948312000	28.930037000
C	9.063320000	18.581519000	27.164895000
C	12.562848000	18.180029000	28.054252000
C	10.879833000	19.024676000	25.472067000
H	11.179941000	19.914038000	26.029502000

C	8.879437000	20.505600000	31.132466000
S	14.866384000	19.924023000	30.898547000
C	15.195279000	19.113864000	32.595880000
H	15.367310000	18.038913000	32.425823000
C	9.786705000	18.246999000	25.928374000
C	11.973765000	20.336687000	29.049342000
H	11.337695000	20.915023000	29.726897000
C	11.772920000	21.033825000	34.340033000
S	13.647786000	21.133243000	33.894453000
H	11.702485000	20.195581000	35.051090000
H	11.594696000	21.979704000	34.875937000
C	16.037293000	22.345209000	24.458286000
C	13.827412000	20.194494000	27.464305000
H	14.631510000	20.675529000	26.894887000
C	12.998507000	20.976200000	28.311002000
C	7.744092000	20.313198000	30.133901000
H	7.386471000	21.277926000	29.741144000
H	6.894738000	19.791982000	30.605801000
C	13.961937000	19.292447000	33.473172000
H	14.089526000	18.755327000	34.425043000
H	13.056649000	18.917990000	32.969360000
C	10.310501000	21.975948000	32.480028000
H	10.644079000	22.984734000	32.748895000
C	10.840399000	20.855264000	33.171183000
C	9.448792000	19.380119000	31.779967000
H	9.110796000	18.370913000	31.519184000
C	13.607793000	18.808740000	27.333355000

H	14.239847000	18.216437000	26.661746000
C	10.415854000	19.553749000	32.789576000
H	10.806526000	18.675740000	33.319467000
S	15.190074000	24.771298000	31.769881000
C	14.777422000	25.168463000	29.952529000
H	15.069576000	26.212609000	29.751608000
H	15.370613000	24.488834000	29.317170000
C	15.763082000	23.526317000	25.186421000
H	14.978362000	24.207822000	24.840875000
C	13.274511000	24.978897000	29.746274000
S	12.705047000	23.188173000	30.065728000
H	12.993793000	25.226496000	28.708397000
H	12.682685000	25.611806000	30.427793000
C	13.213457000	22.469239000	28.366060000
H	12.583413000	22.994665000	27.625694000
H	14.268630000	22.729689000	28.182319000
C	18.020018000	14.799450000	33.383204000
N	19.354310000	14.776322000	33.824111000
C	17.658586000	14.276648000	32.053391000
C	18.419842000	13.204404000	31.511694000
H	19.227577000	12.768241000	32.107461000
C	16.314295000	14.353057000	29.985592000
C	17.233766000	15.387081000	34.400865000
I	15.139382000	15.671527000	34.591764000
N	18.122432000	15.700189000	35.411469000
C	17.883844000	16.500657000	36.628952000
H	17.016859000	16.084598000	37.166658000

H	18.783700000	16.375395000	37.253419000
N	19.437188000	15.332586000	35.059331000
C	17.084308000	13.275213000	29.462240000
H	16.853255000	12.893108000	28.463441000
H	18.714951000	11.881895000	29.811122000
C	18.130207000	12.711564000	30.221735000
C	16.610759000	14.848521000	31.282663000
H	16.056732000	15.705712000	31.675396000
H	16.100574000	19.562671000	33.036447000
C	18.419336000	19.979869000	35.091186000
C	17.208866000	20.647575000	35.423149000
H	19.179587000	20.505512000	34.502326000
C	17.092650000	24.683832000	31.663106000
H	17.365220000	24.133016000	30.747762000
H	17.476471000	25.714654000	31.594123000
C	17.640555000	24.002561000	32.912583000
S	16.994821000	22.209565000	33.063765000
H	17.337870000	24.535888000	33.828924000
H	18.740624000	23.957683000	32.863646000
C	16.981763000	22.074765000	34.989314000
H	16.017986000	22.470053000	35.341917000
H	17.803005000	22.734480000	35.316279000
C	16.228171000	19.956624000	36.176288000
H	15.286847000	20.458307000	36.423388000
C	18.637681000	18.650639000	35.497582000
H	19.567373000	18.138373000	35.223872000
C	17.652638000	17.960255000	36.251930000

C	16.449645000	18.621734000	36.589926000
H	15.677540000	18.089876000	37.155716000
C	16.732649000	21.500769000	29.540425000
S	18.564603000	21.951422000	29.295505000
H	16.264832000	22.300470000	30.141123000
C	17.048937000	21.464231000	24.922955000
H	17.257011000	20.540297000	24.371251000
C	16.501511000	23.832205000	26.354264000
H	16.287096000	24.753711000	26.908374000
C	16.648827000	20.145416000	30.246320000
H	17.310558000	20.098212000	31.126747000
H	16.875534000	19.314882000	29.561433000
C	17.535765000	22.971600000	26.801282000
C	18.365677000	23.354508000	28.006919000
H	17.944519000	24.239605000	28.511083000
H	19.410136000	23.579029000	27.726540000
C	17.791581000	21.775860000	26.078098000
H	18.572121000	21.092554000	26.430135000
H	16.254337000	21.470273000	28.548847000
H	9.949252000	15.696372000	23.616689000
I	11.683639000	16.241338000	34.590798000
Li	14.467925000	22.277398000	31.823472000

Table S12: Cartesian Coordenates of **IS**-Na^+I^-

**IS**-$Na^+.I^-$	x	y	z
C	10.184937000	16.784679000	23.994628000
C	11.263407000	17.580939000	23.555330000

H	11.831927000	17.303646000	22.662446000
I	12.952672000	15.952266000	31.457510000
C	13.837381000	15.591231000	29.566538000
C	9.464597000	17.140022000	25.154027000
H	8.636204000	16.518444000	25.507203000
N	8.635172000	17.521381000	27.927666000
N	8.082308000	18.002823000	29.069524000
N	15.034533000	14.808719000	27.789761000
N	13.947581000	15.434910000	27.275480000
N	13.210096000	15.909302000	28.376792000
C	15.006701000	14.885272000	29.192536000
C	12.054334000	16.791971000	28.139597000
H	11.674295000	16.536521000	27.136502000
H	11.269607000	16.561796000	28.876862000
N	14.368048000	21.022263000	23.329480000
C	13.130943000	20.889007000	23.932148000
N	13.860221000	18.875611000	23.171113000
N	14.825711000	19.777659000	22.863211000
C	11.626829000	18.751615000	24.275968000
C	12.800240000	19.518938000	23.829265000
C	15.248083000	22.201673000	23.233030000
H	14.644077000	23.070605000	22.926046000
H	15.962526000	21.970295000	22.425663000
I	12.049676000	22.529052000	24.634775000
N	8.221385000	19.400348000	29.018349000
C	8.860631000	19.781691000	27.853167000
I	9.177739000	21.782384000	27.359285000

H	9.178161000	22.457575000	31.068655000
C	9.500781000	21.545984000	31.584262000
H	11.014717000	18.792015000	29.726258000
C	11.810932000	19.152381000	29.068393000
C	9.126581000	18.583271000	27.153425000
C	12.474845000	18.252842000	28.209687000
C	10.900078000	19.108730000	25.440105000
H	11.215647000	19.969663000	26.033414000
C	8.934465000	20.297158000	31.224641000
S	15.073103000	19.555195000	30.989075000
C	15.329518000	18.920169000	32.774292000
H	15.649616000	17.868231000	32.704161000
C	9.820566000	18.306172000	25.886556000
C	12.164344000	20.521132000	29.081642000
H	11.603429000	21.197730000	29.735887000
C	11.752118000	20.531520000	34.535209000
S	13.605574000	20.784484000	34.096430000
H	11.705752000	19.613777000	35.142190000
H	11.521109000	21.400500000	35.172603000
C	15.973723000	22.466393000	24.546237000
C	13.887040000	20.089875000	27.407658000
H	14.695064000	20.451066000	26.760745000
C	13.197218000	21.007801000	28.246094000
C	7.819885000	20.206285000	30.189045000
H	7.515564000	21.203239000	29.834454000
H	6.936384000	19.701890000	30.613490000
C	14.034647000	18.997182000	33.576932000

H	14.141051000	18.411009000	34.502562000
H	13.175398000	18.604007000	33.010056000
C	10.449045000	21.623644000	32.629904000
H	10.849962000	22.600580000	32.930072000
C	10.846105000	20.453710000	33.332610000
C	9.377409000	19.121707000	31.881308000
H	8.959459000	18.149778000	31.596701000
C	13.528585000	18.729546000	27.388384000
H	14.056687000	18.035027000	26.725694000
C	10.324747000	19.198715000	32.921923000
H	10.635247000	18.287626000	33.448506000
S	14.962228000	24.872029000	31.423263000
C	14.954271000	25.199126000	29.546504000
H	15.420314000	26.184000000	29.372815000
H	15.571291000	24.420113000	29.072160000
C	15.877116000	23.722422000	25.189227000
H	15.245461000	24.506977000	24.757795000
C	13.524113000	25.207310000	29.007088000
S	12.556089000	23.589306000	29.293350000
H	13.538556000	25.383753000	27.918026000
H	12.911663000	25.990629000	29.483162000
C	13.625662000	22.460742000	28.189783000
H	13.548627000	22.852921000	27.162097000
H	14.673326000	22.569325000	28.516380000
C	18.074062000	14.844948000	33.208388000
N	19.443880000	14.935254000	33.504240000
C	17.609623000	14.271387000	31.932911000

C	18.327196000	13.185919000	31.360599000
H	19.184434000	12.768042000	31.897751000
C	16.101907000	14.293625000	29.981463000
C	17.353326000	15.399167000	34.290787000
I	15.273112000	15.527384000	34.680369000
N	18.314429000	15.811394000	35.193276000
C	18.144440000	16.612173000	36.420475000
H	17.354228000	16.154148000	37.036940000
H	19.102566000	16.540148000	36.961358000
N	19.610282000	15.533075000	34.712340000
C	16.826014000	13.200491000	29.426626000
H	16.516972000	12.793464000	28.459123000
H	18.481255000	11.814927000	29.679423000
C	17.930878000	12.655922000	30.113748000
C	16.500811000	14.821575000	31.237136000
H	15.981365000	15.690822000	31.650648000
H	16.141142000	19.509560000	33.231009000
C	18.368424000	20.144513000	34.912580000
C	17.189431000	20.761735000	35.413121000
H	19.040709000	20.715788000	34.262278000
C	16.853174000	24.835019000	31.696141000
H	17.303341000	24.227361000	30.893090000
H	17.223367000	25.870364000	31.618907000
C	17.178689000	24.265346000	33.074053000
S	16.639499000	22.438726000	33.187796000
H	16.668611000	24.822049000	33.878173000
H	18.268356000	24.303791000	33.241034000

C	16.873559000	22.201454000	35.084554000
H	15.952877000	22.543633000	35.579760000
H	17.710124000	22.870905000	35.348778000
C	16.319950000	20.006223000	36.238084000
H	15.398349000	20.464352000	36.609643000
C	18.671623000	18.808312000	35.238286000
H	19.580946000	18.339849000	34.843997000
C	17.804312000	18.058773000	36.076494000
C	16.624645000	18.664518000	36.567314000
H	15.938466000	18.087335000	37.196292000
C	16.833492000	21.315697000	29.702090000
S	18.621827000	21.882707000	29.365132000
H	16.316341000	22.141889000	30.221627000
C	16.776666000	21.452048000	25.127958000
H	16.841339000	20.469132000	24.647144000
C	16.597916000	23.970481000	26.380360000
H	16.531493000	24.953835000	26.860161000
C	16.871684000	20.052584000	30.560801000
H	17.387504000	20.233467000	31.517888000
H	17.340616000	19.210843000	30.028490000
C	17.434206000	22.971895000	26.943438000
C	18.272110000	23.318313000	28.160819000
H	17.813784000	24.147872000	28.723794000
H	19.286468000	23.638112000	27.858893000
C	17.499112000	21.702953000	26.311786000
H	18.123866000	20.917149000	26.751303000
I	11.782831000	16.026993000	34.904331000

H	16.351655000	21.124597000	28.729793000
H	9.908098000	15.885448000	23.435337000
Na	13.905249000	22.141492000	31.609812000

Table S13: Cartesian Coordenates of **IS**-K^+.I^-

**IS**-K^+I^-	x	y	z
C	10.007913000	17.026852000	23.929971000
C	11.122102000	17.795019000	23.532729000
H	11.677313000	17.540697000	22.624724000
I	12.924118000	15.892470000	31.455155000
C	13.721498000	15.502251000	29.531781000
C	9.307939000	17.349059000	25.111263000
H	8.452322000	16.747267000	25.432472000
N	8.538349000	17.598009000	27.921445000
N	8.009489000	18.035026000	29.091443000
N	14.824542000	14.652714000	27.725127000
N	13.716711000	15.268737000	27.244569000
N	13.036411000	15.785357000	28.364593000
C	14.868800000	14.779519000	29.123814000
C	11.907958000	16.709564000	28.152743000
H	11.476117000	16.451272000	27.171241000
H	11.145110000	16.531773000	28.926376000
N	14.414488000	21.057798000	23.485260000
C	13.167252000	20.970839000	24.076015000
N	13.784152000	18.953706000	23.229358000
N	14.801841000	19.810136000	22.964842000
C	11.542303000	18.904891000	24.316385000

C	12.758342000	19.628277000	23.910448000
C	15.349259000	22.193866000	23.413188000
H	14.787584000	23.096870000	23.123380000
H	16.050169000	21.945346000	22.598468000
I	12.195452000	22.631113000	24.882181000
N	8.178622000	19.431652000	29.107258000
C	8.810157000	19.854557000	27.951347000
I	9.167257000	21.865375000	27.533105000
H	8.826072000	22.363692000	31.653113000
C	9.259794000	21.418118000	31.999146000
H	10.960271000	18.825682000	29.650983000
C	11.781937000	19.115957000	28.990620000
C	9.042640000	18.685655000	27.192393000
C	12.402611000	18.149306000	28.172135000
C	10.833831000	19.231319000	25.501008000
H	11.189145000	20.043703000	26.138942000
C	8.881346000	20.201784000	31.376657000
S	15.092378000	19.590012000	31.200388000
C	15.274251000	18.910547000	32.975173000
H	15.678203000	17.889481000	32.890081000
C	9.719334000	18.454156000	25.906372000
C	12.219159000	20.459624000	28.966516000
H	11.678766000	21.195112000	29.574157000
C	11.580039000	20.158936000	34.805074000
S	13.429615000	20.550850000	34.432401000
H	11.572752000	19.168566000	35.288191000
H	11.296385000	20.927300000	35.542219000

C	16.095503000	22.408263000	24.723872000
C	13.925228000	19.876452000	27.326826000
H	14.755106000	20.166939000	26.674329000
C	13.301972000	20.857555000	28.144820000
C	7.788293000	20.186494000	30.311937000
H	7.511376000	21.208189000	30.008032000
H	6.885033000	19.687317000	30.702063000
C	13.932649000	18.860634000	33.699630000
H	13.991282000	18.158618000	34.544904000
H	13.117607000	18.531053000	33.034680000
C	10.152473000	21.411742000	33.096799000
H	10.391769000	22.353772000	33.608200000
C	10.697998000	20.190515000	33.581528000
C	9.459613000	18.987930000	31.827804000
H	9.177952000	18.042160000	31.351619000
C	13.482238000	18.540762000	27.340640000
H	13.966285000	17.800837000	26.693605000
C	10.361105000	18.983018000	32.911924000
H	10.770397000	18.033418000	33.278469000
S	14.869280000	25.063172000	31.099622000
C	15.132869000	25.083791000	29.212885000
H	15.705143000	25.995869000	28.970040000
H	15.743299000	24.203818000	28.958817000
C	16.169294000	23.695506000	25.305537000
H	15.646043000	24.535606000	24.835062000
C	13.810450000	25.103427000	28.448042000
S	12.674283000	23.598567000	28.735258000

H	14.019190000	25.138408000	27.365003000
H	13.193982000	25.977100000	28.717602000
C	13.890260000	22.254704000	28.138303000
H	14.216831000	22.538712000	27.124893000
H	14.769526000	22.303435000	28.801223000
C	18.135832000	14.983878000	32.959907000
N	19.508860000	15.202956000	33.149887000
C	17.626078000	14.335215000	31.738618000
C	18.344865000	13.249047000	31.170557000
H	19.243933000	12.878592000	31.673189000
C	16.013280000	14.235937000	29.875900000
C	17.446577000	15.509129000	34.076682000
I	15.395278000	15.455809000	34.600478000
N	18.428138000	16.035793000	34.893495000
C	18.282657000	16.838775000	36.120146000
H	17.536423000	16.355559000	36.771234000
H	19.263730000	16.801348000	36.622847000
N	19.706387000	15.853835000	34.325850000
C	16.737934000	13.141208000	29.325204000
H	16.390532000	12.687995000	28.391808000
H	18.445872000	11.814360000	29.538783000
C	17.894986000	12.656475000	29.970264000
C	16.464054000	14.823674000	31.086493000
H	15.944926000	15.695045000	31.495611000
H	16.008950000	19.540790000	33.499694000
C	18.320490000	20.396967000	34.645885000
C	17.215335000	21.005468000	35.302564000

H	18.920076000	20.986634000	33.942797000
C	16.712709000	25.018200000	31.608245000
H	17.231635000	24.338524000	30.911482000
H	17.115456000	26.037690000	31.489954000
C	16.882817000	24.555846000	33.053294000
S	16.297065000	22.750532000	33.252860000
H	16.321825000	25.189672000	33.760295000
H	17.954274000	24.581431000	33.317246000
C	16.881490000	22.458694000	35.068152000
H	16.082091000	22.810258000	35.737122000
H	17.769248000	23.103935000	35.183638000
C	16.437628000	20.223200000	36.192300000
H	15.569309000	20.669218000	36.686366000
C	18.652627000	19.050400000	34.895193000
H	19.511747000	18.595386000	34.388801000
C	17.884195000	18.277681000	35.806328000
C	16.766685000	18.869331000	36.439244000
H	16.152201000	18.275744000	37.124938000
C	16.944483000	21.239726000	29.906575000
S	18.759230000	21.695429000	29.535332000
H	16.452411000	22.131637000	30.332143000
C	16.765211000	21.328948000	25.353679000
H	16.709473000	20.325433000	24.916137000
C	16.922349000	23.904143000	26.482951000
H	16.990914000	24.911311000	26.909663000
C	16.916268000	20.077962000	30.895793000
H	17.333213000	20.375074000	31.871767000

H	17.444287000	19.191288000	30.509856000
C	17.616307000	22.832121000	27.103387000
C	18.480460000	23.132912000	28.317077000
H	18.073705000	23.991398000	28.876970000
H	19.508623000	23.394989000	28.005769000
C	17.514537000	21.538853000	26.530589000
H	18.035550000	20.699583000	27.005382000
I	11.866386000	15.796795000	34.957185000
H	16.471892000	20.963001000	28.950282000
H	9.688058000	16.174912000	23.321724000
K	13.373777000	22.278962000	31.778911000

Table S14: Cartesian Coordenates of **IS**-$Cu^{+}I^{-}$

**IS**-$Cu^{+}.I^{-}$	x	y	z
C	9.331617000	16.749465000	24.518854000
C	10.578366000	17.219158000	24.052760000
H	11.114567000	16.686236000	23.261488000
I	13.486752000	16.009717000	30.647231000
C	14.747839000	15.801113000	28.954970000
C	8.659231000	17.424890000	25.558815000
H	7.705164000	17.048532000	25.939913000
N	7.786862000	18.376566000	28.158427000
N	7.360909000	19.095540000	29.224893000
N	16.375005000	15.292751000	27.438858000
N	15.356053000	15.846772000	26.737226000
N	14.352564000	16.149381000	27.676698000
C	16.040612000	15.242215000	28.803035000

C	13.112461000	16.806182000	27.220937000
H	13.089905000	16.665033000	26.126654000
H	12.250868000	16.282109000	27.665243000
N	14.370962000	19.860923000	23.603611000
C	13.079881000	20.087170000	24.041205000
N	13.457004000	17.857681000	23.826908000
N	14.610613000	18.481047000	23.473547000
C	11.151555000	18.391084000	24.616742000
C	12.491678000	18.811585000	24.179137000
C	15.443201000	20.818598000	23.256355000
H	14.997145000	21.616347000	22.640188000
H	16.148194000	20.244982000	22.632904000
I	12.283524000	21.997819000	24.298471000
N	7.884370000	20.392353000	29.065935000
C	8.632597000	20.471197000	27.903818000
I	9.403344000	22.280245000	27.205677000
H	8.746794000	23.317815000	31.809366000
C	9.263780000	22.363019000	31.961114000
H	11.300185000	18.152974000	28.791272000
C	12.073158000	18.815799000	28.389101000
C	8.578091000	19.182668000	27.322968000
C	13.097986000	18.282666000	27.576320000
C	10.467615000	19.076845000	25.650960000
H	10.938801000	19.940099000	26.122826000
C	8.926250000	21.245827000	31.158101000
S	14.790521000	19.835636000	30.792268000
C	15.314383000	19.141770000	32.498615000

H	15.560080000	18.078063000	32.349643000
C	9.229024000	18.591239000	26.141491000
C	12.037524000	20.193672000	28.677926000
H	11.239586000	20.586550000	29.313955000
C	11.868274000	20.861620000	34.344659000
S	13.729531000	21.097857000	33.846936000
H	11.830801000	19.857252000	34.796230000
H	11.710074000	21.621558000	35.125030000
C	16.144400000	21.402301000	24.474562000
C	14.079006000	20.517833000	27.378239000
H	14.857593000	21.177049000	26.982543000
C	13.037174000	21.059811000	28.171496000
C	7.759936000	21.348890000	30.175259000
H	7.673229000	22.367378000	29.762857000
H	6.810207000	21.105814000	30.682698000
C	14.168415000	19.264216000	33.489678000
H	14.455383000	18.815506000	34.452245000
H	13.253216000	18.771775000	33.125193000
C	10.243485000	22.247656000	32.971404000
H	10.480199000	23.112106000	33.603303000
C	10.906921000	21.012920000	33.199528000
C	9.625786000	20.028147000	31.346933000
H	9.384965000	19.150049000	30.739371000
C	14.106899000	19.141717000	27.077953000
H	14.912636000	18.734284000	26.457247000
C	10.606413000	19.914590000	32.353332000
H	11.101812000	18.949603000	32.514238000

S	14.761858000	24.222508000	32.080651000
C	14.127049000	25.075419000	30.494553000
H	14.260643000	26.162820000	30.621351000
H	14.754742000	24.724333000	29.658149000
C	15.970252000	22.763819000	24.817582000
H	15.308514000	23.394201000	24.214524000
C	12.647915000	24.757421000	30.284592000
S	12.264924000	22.890565000	30.164447000
H	12.287038000	25.215530000	29.348293000
H	12.025873000	25.118992000	31.119710000
C	13.011577000	22.546309000	28.432855000
H	12.359411000	23.080135000	27.718057000
H	14.024079000	22.977276000	28.396821000
C	18.148568000	14.844970000	33.403054000
N	19.407627000	14.784201000	34.025782000
C	17.993398000	14.415312000	32.001365000
C	18.863128000	13.406129000	31.502502000
H	19.586013000	12.941809000	32.180168000
C	16.975788000	14.625922000	29.763310000
C	17.227657000	15.379108000	34.334154000
I	15.127464000	15.659026000	34.247819000
N	17.966617000	15.627554000	35.476212000
C	17.580405000	16.397693000	36.674558000
H	16.625876000	16.007334000	37.062213000
H	18.369613000	16.210422000	37.421543000
N	19.317278000	15.268147000	35.289164000
C	17.855566000	13.614411000	29.282762000

H	17.792296000	13.309112000	28.233977000
H	19.460808000	12.233573000	29.772085000
C	18.791635000	13.013715000	30.149560000
C	17.054214000	15.022516000	31.124463000
H	16.415201000	15.831186000	31.490482000
H	16.208852000	19.696583000	32.821733000
C	18.449530000	19.873405000	35.280026000
C	17.266584000	20.614962000	35.549440000
H	19.282758000	20.359323000	34.760020000
C	16.623556000	24.607987000	31.850528000
H	16.954173000	24.180273000	30.889897000
H	16.715508000	25.706770000	31.807947000
C	17.424700000	24.065158000	33.030474000
S	17.269175000	22.168176000	33.199493000
H	17.079704000	24.504211000	33.981546000
H	18.491199000	24.307397000	32.888833000
C	17.153013000	22.058008000	35.118851000
H	16.189702000	22.499118000	35.417221000
H	17.985408000	22.669840000	35.505852000
C	16.196844000	19.972867000	36.221058000
H	15.278179000	20.532073000	36.426317000
C	18.550552000	18.521808000	35.659897000
H	19.461731000	17.956181000	35.433342000
C	17.472283000	17.879049000	36.323285000
C	16.298116000	18.614347000	36.605090000
H	15.454754000	18.123337000	37.102933000
C	16.620734000	21.681272000	29.709077000

S	18.449667000	22.142126000	29.432137000
H	16.223264000	22.287307000	30.538714000
C	17.000938000	20.595860000	25.267850000
H	17.135625000	19.537878000	25.014989000
C	16.645355000	23.313418000	25.931079000
H	16.502867000	24.369969000	26.186631000
C	16.520549000	20.197715000	30.051647000
H	17.248238000	19.900634000	30.823837000
H	16.636444000	19.553817000	29.167593000
C	17.527526000	22.518997000	26.707544000
C	18.308359000	23.174235000	27.829063000
H	17.875334000	24.155012000	28.084125000
H	19.364930000	23.327797000	27.543370000
C	17.686604000	21.148143000	26.369341000
H	18.349390000	20.519261000	26.973445000
H	16.074817000	21.921277000	28.784078000
H	8.888229000	15.851297000	24.077426000
I	11.664513000	16.244529000	33.725034000
Cu	13.915173000	21.932709000	31.612804000

Table S15: Cartesian Coordenates of **IS**-$Ag^{+}.I^{-}$

**IS**-$Ag^{+}.I^{-}$	x	y	z
C	10.204814000	16.566213000	24.185989000
C	11.290276000	17.330982000	23.707637000
H	11.883917000	16.983240000	22.856671000
I	13.064118000	16.034534000	31.287752000
C	14.076688000	15.663932000	29.461789000

C	9.455501000	17.009384000	25.295849000
H	8.626125000	16.410042000	25.683321000
N	8.579614000	17.586770000	28.011741000
N	8.047365000	18.137043000	29.131691000
N	15.419217000	14.927958000	27.770814000
N	14.355149000	15.527772000	27.183155000
N	13.527700000	15.971315000	28.230899000
C	15.287228000	14.988479000	29.168792000
C	12.326485000	16.763548000	27.912838000
H	12.061972000	16.508793000	26.873146000
H	11.503703000	16.440689000	28.570616000
N	14.366123000	20.792696000	23.301364000
C	13.104552000	20.684761000	23.854703000
N	13.907030000	18.628398000	23.317282000
N	14.869521000	19.522050000	22.972316000
C	11.625992000	18.561676000	24.334853000
C	12.805149000	19.304180000	23.862821000
C	15.222003000	21.985284000	23.129748000
H	14.593106000	22.813919000	22.767482000
H	15.944439000	21.719438000	22.341380000
I	11.959888000	22.347589000	24.376589000
N	8.214272000	19.526873000	29.008106000
C	8.847701000	19.835250000	27.817953000
I	9.182760000	21.807149000	27.222205000
H	9.142331000	22.712834000	30.944219000
C	9.511171000	21.823703000	31.467117000
H	11.052560000	18.561838000	29.556077000

C	11.813037000	19.038015000	28.929753000
C	9.085537000	18.596840000	27.179301000
C	12.594548000	18.252855000	28.055535000
C	10.868325000	19.007229000	25.447321000
H	11.167690000	19.914358000	25.975234000
C	8.988645000	20.547910000	31.139608000
S	14.903637000	19.656174000	30.824817000
C	15.255639000	19.042637000	32.603082000
H	15.487082000	17.967508000	32.534066000
C	9.788212000	18.233781000	25.939108000
C	11.999895000	20.434049000	28.992348000
H	11.364628000	21.021646000	29.662431000
C	11.854516000	20.958236000	34.383951000
S	13.729359000	21.070028000	33.934022000
H	11.775956000	20.102147000	35.072458000
H	11.679958000	21.892934000	34.940271000
C	15.933040000	22.351322000	24.425895000
C	13.807834000	20.265041000	27.358380000
H	14.582500000	20.739998000	26.744712000
C	12.982709000	21.065034000	28.192870000
C	7.846374000	20.399421000	30.140847000
H	7.532015000	21.375536000	29.740059000
H	6.974788000	19.921404000	30.617806000
C	14.028825000	19.232810000	33.487256000
H	14.168765000	18.695480000	34.437785000
H	13.113058000	18.860624000	33.000751000
C	10.482057000	21.956880000	32.485880000

H	10.861913000	22.950139000	32.752104000
C	10.936359000	20.817201000	33.201092000
C	9.493528000	19.401500000	31.803375000
H	9.107411000	18.408858000	31.546646000
C	13.611887000	18.871465000	27.287403000
H	14.235451000	18.265954000	26.619880000
C	10.454988000	19.535310000	32.824078000
H	10.799615000	18.644683000	33.364789000
S	15.097924000	24.641114000	31.683369000
C	14.848335000	25.075346000	29.844767000
H	15.278417000	26.076841000	29.673105000
H	15.400159000	24.333618000	29.243988000
C	15.604982000	23.530678000	25.134759000
H	14.818120000	24.191585000	24.755790000
C	13.358323000	25.093119000	29.510191000
S	12.491762000	23.406998000	29.729740000
H	13.208510000	25.384575000	28.456760000
H	12.799725000	25.794117000	30.151613000
C	13.149669000	22.565294000	28.143726000
H	12.572739000	23.003955000	27.310360000
H	14.208769000	22.846649000	28.026883000
C	18.014824000	14.768248000	33.435359000
N	19.348132000	14.733217000	33.878055000
C	17.652609000	14.263209000	32.099106000
C	18.404091000	13.188114000	31.549610000
H	19.203679000	12.736396000	32.144812000
C	16.320748000	14.378130000	30.025718000

C	17.229792000	15.348338000	34.458044000
I	15.135473000	15.633846000	34.646473000
N	18.118188000	15.646740000	35.473169000
C	17.883979000	16.441195000	36.695880000
H	17.001656000	16.039296000	37.219056000
H	18.773453000	16.289479000	37.329134000
N	19.431519000	15.276006000	35.119328000
C	17.080726000	13.297243000	29.494295000
H	16.850524000	12.928909000	28.490125000
H	18.692742000	11.880552000	29.836389000
C	18.115683000	12.712628000	30.252921000
C	16.615325000	14.855742000	31.329742000
H	16.068472000	15.715044000	31.728259000
H	16.136389000	19.584693000	32.982407000
C	18.535543000	19.931977000	35.229189000
C	17.328092000	20.619206000	35.532011000
H	19.326988000	20.452631000	34.678275000
C	17.005945000	24.587699000	31.697394000
H	17.347407000	23.978406000	30.843328000
H	17.358778000	25.624715000	31.568232000
C	17.513193000	24.022886000	33.020520000
S	17.089849000	22.170813000	33.184545000
H	17.069949000	24.546604000	33.884548000
H	18.610652000	24.124906000	33.064765000
C	17.141952000	22.054837000	35.105329000
H	16.202076000	22.480487000	35.488259000
H	17.993845000	22.684368000	35.414118000

C	16.308241000	19.933775000	36.237432000
H	15.369956000	20.450405000	36.464529000
C	18.714265000	18.592043000	35.621038000
H	19.643227000	18.066023000	35.371916000
C	17.691144000	17.909137000	36.330075000
C	16.489020000	18.588054000	36.635657000
H	15.688567000	18.062743000	37.167196000
C	16.643055000	21.469471000	29.563249000
S	18.438354000	22.051681000	29.297236000
H	16.119846000	22.213834000	30.191919000
C	16.945185000	21.496896000	24.935803000
H	17.193110000	20.572793000	24.401174000
C	16.293301000	23.862564000	26.326002000
H	16.037361000	24.783542000	26.862944000
C	16.673044000	20.105579000	30.251290000
H	17.301980000	20.116700000	31.156231000
H	17.010219000	19.310270000	29.569406000
C	17.332240000	23.031240000	26.815740000
C	18.121608000	23.453659000	28.036686000
H	17.628517000	24.290591000	28.557967000
H	19.143167000	23.773129000	27.762888000
C	17.638259000	21.834669000	26.114019000
H	18.420577000	21.172369000	26.500538000
I	11.684719000	16.221755000	34.633139000
H	16.156659000	21.415123000	28.576392000
H	9.947229000	15.621128000	23.697554000
Ag	13.990946000	22.089349000	31.521459000

Table S16: Cartesian Coordenates of **IS**-$Au^{+}.I^{-}$

**IS**-$Au^{+}.I^{-}$	x	y	z
C	10.186166000	16.581821000	24.168629000
C	11.262811000	17.355601000	23.685016000
H	11.851549000	17.015648000	22.827507000
I	13.061904000	16.031297000	31.251714000
C	14.106425000	15.663347000	29.442850000
C	9.444092000	17.014596000	25.287445000
H	8.621771000	16.408191000	25.678958000
N	8.589818000	17.572558000	28.015047000
N	8.064103000	18.114136000	29.142298000
N	15.485474000	14.936185000	27.777337000
N	14.428185000	15.527252000	27.169657000
N	13.578187000	15.965718000	28.201368000
C	15.326933000	14.995862000	29.172636000
C	12.373531000	16.742139000	27.856925000
H	12.133888000	16.480343000	26.812903000
H	11.541972000	16.410743000	28.499227000
N	14.324059000	20.831057000	23.282251000
C	13.064994000	20.715664000	23.839751000
N	13.872228000	18.665208000	23.287324000
N	14.830226000	19.564073000	22.943509000
C	11.597357000	18.584381000	24.316624000
C	12.770351000	19.334165000	23.841246000
C	15.177501000	22.026552000	23.119702000
H	14.547529000	22.857300000	22.764253000
H	15.900212000	21.768208000	22.329049000

I	11.916866000	22.372094000	24.374082000
N	8.219882000	19.505808000	29.023174000
C	8.840144000	19.823668000	27.828651000
I	9.153773000	21.800510000	27.237015000
H	9.076041000	22.712929000	30.991051000
C	9.482648000	21.827923000	31.492641000
H	11.073637000	18.520033000	29.500869000
C	11.825950000	19.007815000	28.873926000
C	9.080550000	18.589774000	27.182253000
C	12.619153000	18.235095000	27.998979000
C	10.847292000	19.019295000	25.438366000
H	11.147283000	19.924231000	25.969866000
C	9.004020000	20.540079000	31.145759000
S	14.901656000	19.581448000	30.801174000
C	15.294719000	19.025269000	32.587551000
H	15.515231000	17.946214000	32.547711000
C	9.775427000	18.237233000	25.934632000
C	11.992484000	20.405337000	28.938499000
H	11.354009000	20.981325000	29.615585000
C	11.911041000	20.995934000	34.346271000
S	13.787404000	21.108203000	33.847381000
H	11.865501000	20.142872000	35.041643000
H	11.755208000	21.935270000	34.899129000
C	15.888160000	22.382179000	24.418949000
C	13.807685000	20.264266000	27.308466000
H	14.578614000	20.751080000	26.699563000
C	12.970474000	21.050194000	28.142961000

C	7.852600000	20.371492000	30.160663000
H	7.515672000	21.342335000	29.765191000
H	6.995609000	19.878420000	30.648870000
C	14.104507000	19.248684000	33.509956000
H	14.307498000	18.795851000	34.492663000
H	13.173970000	18.824959000	33.101028000
C	10.459343000	21.977585000	32.503288000
H	10.806632000	22.978607000	32.783155000
C	10.966925000	20.841501000	33.188441000
C	9.556639000	19.400834000	31.783299000
H	9.201187000	18.399458000	31.516423000
C	13.628817000	18.868244000	27.232978000
H	14.261288000	18.272615000	26.565039000
C	10.525637000	19.550992000	32.794255000
H	10.902538000	18.662851000	33.316548000
S	15.166067000	24.686379000	31.723910000
C	14.895842000	25.028173000	29.873041000
H	15.328411000	26.015426000	29.635728000
H	15.428345000	24.252227000	29.299530000
C	15.566706000	23.560429000	25.132496000
H	14.784252000	24.227880000	24.755742000
C	13.404835000	25.047109000	29.551599000
S	12.533912000	23.355380000	29.746004000
H	13.239492000	25.339029000	28.500660000
H	12.844333000	25.736194000	30.203901000
C	13.126028000	22.550198000	28.109333000
H	12.498397000	23.009131000	27.325173000

H	14.174758000	22.848539000	27.951864000
C	17.981271000	14.766366000	33.487654000
N	19.306089000	14.723713000	33.955117000
C	17.642635000	14.266863000	32.143126000
C	18.407741000	13.197192000	31.601729000
H	19.197775000	12.745023000	32.209150000
C	16.347646000	14.387630000	30.046702000
C	17.180800000	15.349009000	34.496652000
I	15.084797000	15.641799000	34.649364000
N	18.051470000	15.640555000	35.529001000
C	17.799441000	16.442031000	36.743520000
H	16.898903000	16.055140000	37.246469000
H	18.670835000	16.280003000	37.399022000
N	19.369299000	15.263346000	35.198795000
C	17.121479000	13.312364000	29.523674000
H	16.910776000	12.948444000	28.513653000
H	18.732208000	11.899658000	29.887422000
C	18.144339000	12.727136000	30.297952000
C	16.616838000	14.859535000	31.358429000
H	16.058176000	15.713560000	31.751827000
H	16.190405000	19.572256000	32.921521000
C	18.535203000	19.910329000	35.264763000
C	17.341123000	20.625772000	35.554944000
H	19.342319000	20.411410000	34.718583000
C	17.068884000	24.583355000	31.705944000
H	17.378098000	23.956280000	30.852348000
H	17.458117000	25.606172000	31.563954000

C	17.583363000	24.009123000	33.023190000
S	17.129535000	22.164355000	33.198679000
H	17.160760000	24.540791000	33.893030000
H	18.683090000	24.091450000	33.054956000
C	17.192967000	22.062877000	35.118778000
H	16.268145000	22.517919000	35.505407000
H	18.063971000	22.669225000	35.420684000
C	16.300417000	19.965278000	36.253657000
H	15.373714000	20.505089000	36.475963000
C	18.681662000	18.569037000	35.664467000
H	19.601371000	18.022169000	35.426307000
C	17.637928000	17.911368000	36.367448000
C	16.447668000	18.616933000	36.658417000
H	15.631156000	18.111712000	37.185213000
C	16.598879000	21.462004000	29.560741000
S	18.392949000	22.052442000	29.291807000
H	16.070723000	22.181315000	30.211583000
C	16.893533000	21.518537000	24.926734000
H	17.135926000	20.595049000	24.388495000
C	16.256470000	23.883155000	26.325601000
H	16.006385000	24.803377000	26.866706000
C	16.653080000	20.084742000	30.219029000
H	17.291561000	20.086642000	31.117265000
H	16.996583000	19.309181000	29.517289000
C	17.289769000	23.043062000	26.812560000
C	18.084725000	23.456724000	28.032509000
H	17.602531000	24.299172000	28.554485000

H	19.108763000	23.765749000	27.755716000
C	17.587547000	21.846671000	26.107017000
H	18.365008000	21.177645000	26.491717000
I	11.638992000	16.218706000	34.570585000
H	16.110020000	21.416667000	28.574728000
H	9.930621000	15.637292000	23.678059000
Au	13.748915000	22.003264000	31.474464000

Table S17: Cartesian Coordenates of **IS**-$Ni^{2+}.PF_6{}^{-}I^{-}$

**IS**-$Ni^{2+}PF_6{}^{-}.I^{-}$	x	y	z
C	9.506112000	17.733251000	23.911083000
C	10.838969000	18.129923000	23.680816000
H	11.404109000	17.719346000	22.838955000
I	13.851860000	15.831977000	30.454468000
C	14.538544000	15.733523000	28.453309000
C	8.796573000	18.243125000	25.018394000
H	7.768931000	17.922894000	25.215962000
N	7.813542000	18.683548000	27.740139000
N	7.250451000	19.270604000	28.825260000
N	15.650117000	15.469016000	26.482074000
N	14.353073000	15.688913000	26.161033000
N	13.665757000	15.840434000	27.383522000
C	15.810672000	15.502891000	27.881207000
C	12.319226000	16.444317000	27.389974000
H	11.952571000	16.392804000	26.350360000
H	11.645525000	15.855199000	28.032676000
N	15.008566000	20.070767000	24.234453000

C	13.734835000	20.440067000	24.634096000
N	13.714754000	18.336674000	23.770776000
N	14.995508000	18.768546000	23.699811000
C	11.474304000	19.057941000	24.553473000
C	12.906291000	19.332353000	24.341119000
C	16.299653000	20.768326000	24.351326000
H	16.128479000	21.849775000	24.225471000
H	16.906986000	20.419701000	23.497814000
I	13.289302000	22.346694000	25.357148000
N	7.757947000	20.582785000	28.876764000
C	8.634792000	20.799231000	27.827951000
I	9.530020000	22.642325000	27.453519000
H	8.250899000	23.542606000	31.541891000
C	8.669309000	22.583019000	31.868020000
H	10.558835000	17.898461000	28.977137000
C	11.439284000	18.478909000	28.681645000
C	8.669787000	19.591595000	27.097613000
C	12.433440000	17.887328000	27.872052000
C	10.750566000	19.589270000	25.651945000
H	11.248075000	20.263446000	26.352400000
C	8.333214000	21.400126000	31.166734000
S	14.888728000	20.786794000	31.361701000
C	14.311539000	19.400337000	32.547030000
H	14.316202000	18.479766000	31.939559000
C	9.415196000	19.174507000	25.896860000
C	11.571480000	19.821325000	29.102246000
H	10.786968000	20.274614000	29.715663000

C	11.006040000	21.245115000	34.606674000
S	12.887506000	21.263805000	34.116661000
H	10.888667000	20.321193000	35.194692000
H	10.910894000	22.121124000	35.266881000
C	17.019547000	20.482122000	25.667016000
C	13.740766000	19.955031000	27.986093000
H	14.649246000	20.512359000	27.740733000
C	12.720972000	20.571186000	28.752651000
C	7.360518000	21.458814000	29.992319000
H	7.262355000	22.488644000	29.612592000
H	6.359844000	21.110664000	30.301446000
C	12.912032000	19.683900000	33.057337000
H	12.596185000	18.844302000	33.699134000
H	12.192317000	19.811110000	32.232960000
C	9.533453000	22.534063000	32.984811000
H	9.794741000	23.457304000	33.515263000
C	10.069981000	21.299518000	33.430516000
C	8.853159000	20.160727000	31.617951000
H	8.581422000	19.233965000	31.100759000
C	13.585308000	18.637740000	27.529427000
H	14.369020000	18.193887000	26.910638000
C	9.701148000	20.110712000	32.742649000
H	10.071283000	19.142822000	33.096681000
S	13.080283000	24.746809000	32.773423000
C	12.398316000	25.271402000	31.059651000
H	11.864796000	26.224658000	31.207052000
H	13.266158000	25.407969000	30.404682000

C	17.848011000	21.481709000	26.235196000
H	17.908760000	22.469139000	25.764083000
C	11.465284000	24.181784000	30.566165000
S	12.182913000	22.415879000	30.823641000
H	11.277802000	24.285005000	29.486706000
H	10.509067000	24.164713000	31.104151000
C	12.898526000	22.027191000	29.089520000
H	12.333873000	22.683487000	28.407475000
H	13.952446000	22.323265000	29.081343000
C	19.212720000	16.567632000	31.488282000
N	20.518241000	17.054096000	31.678952000
C	18.826296000	15.964675000	30.198926000
C	19.845787000	15.379851000	29.396620000
H	20.876682000	15.375661000	29.763259000
C	17.163159000	15.405130000	28.460302000
C	18.506015000	16.736613000	32.702385000
I	16.520752000	16.268836000	33.265348000
N	19.414219000	17.302110000	33.577830000
C	19.225085000	17.720309000	34.983988000
H	18.723093000	16.901858000	35.524761000
H	20.240366000	17.848296000	35.392607000
N	20.658389000	17.508688000	32.948491000
C	18.194895000	14.813317000	27.679226000
H	17.944714000	14.377561000	26.707536000
H	20.314073000	14.356130000	27.537757000
C	19.525280000	14.809019000	28.147150000
C	17.490024000	15.979687000	29.718256000

H	16.711707000	16.477731000	30.301605000
H	15.057732000	19.322868000	33.351779000
C	18.250156000	21.417324000	34.634420000
C	16.901723000	21.411665000	35.084466000
H	18.702394000	22.350199000	34.279103000
C	14.630210000	25.871971000	32.852357000
H	15.013507000	25.972429000	31.827709000
H	14.297561000	26.849380000	33.237663000
C	15.615405000	25.220533000	33.801045000
S	15.962531000	23.392269000	33.276401000
H	15.235105000	25.178233000	34.833723000
H	16.591228000	25.730411000	33.777814000
C	16.099074000	22.679817000	35.080282000
H	15.074497000	22.554876000	35.458334000
H	16.608582000	23.497277000	35.617897000
C	16.329648000	20.201768000	35.548388000
H	15.292639000	20.189500000	35.900317000
C	18.999211000	20.226105000	34.616035000
H	20.027361000	20.230410000	34.239077000
C	18.423192000	19.012111000	35.070909000
C	17.093397000	19.012596000	35.556383000
H	16.647173000	18.082584000	35.920351000
C	17.544214000	21.514641000	30.677978000
S	19.376169000	21.031072000	30.602209000
H	17.524144000	22.523473000	31.112689000
C	16.934547000	19.219005000	26.301218000
H	16.323778000	18.422402000	25.863576000

C	18.590123000	21.219776000	27.406557000
H	19.221835000	22.004087000	27.838190000
C	16.788245000	20.503395000	31.524422000
H	17.046367000	20.547106000	32.591399000
H	16.905032000	19.471211000	31.150769000
C	18.500049000	19.960233000	28.053262000
C	19.296574000	19.625069000	29.298579000
H	20.364064000	19.455548000	29.069893000
H	18.907346000	18.718675000	29.783782000
C	17.650952000	18.971083000	27.492681000
H	17.569163000	17.994958000	27.980744000
Ni	14.021761000	22.737834000	32.231834000
H	17.167021000	21.566649000	29.645428000
H	9.023901000	17.021157000	23.233910000
I	13.053814000	15.934710000	33.936453000
F	14.134300000	24.837156000	28.279171000
F	17.407254000	24.553526000	29.579971000
F	16.416864000	25.440322000	27.464132000
F	15.117681000	23.918833000	30.390897000
F	15.596910000	26.270150000	29.689413000
F	15.919495000	23.095837000	28.174037000
P	15.789788000	24.692319000	28.906310000

Table S18: Cartesian Coordenates of **IS**-Zn^{2+}.$PF_6{}^-I^-$

**IS**-$Zn^{2+}PF_6{}^-.I^-$	x	y	z
C	8.989395000	17.401658000	24.260876000
C	10.302616000	17.791250000	23.921215000

H	10.829272000	17.307526000	23.092893000
I	13.488744000	15.756604000	30.419433000
C	14.779386000	15.622743000	28.745604000
C	8.328683000	18.002515000	25.352229000
H	7.324094000	17.678993000	25.640654000
N	7.432533000	18.659419000	28.025067000
N	7.023769000	19.254009000	29.171604000
N	16.459571000	15.197701000	27.263739000
N	15.411463000	15.663003000	26.536344000
N	14.376343000	15.914253000	27.456255000
C	16.112284000	15.165363000	28.624137000
C	13.135074000	16.571212000	27.002451000
H	13.146868000	16.513099000	25.900070000
H	12.269444000	16.006113000	27.384287000
N	14.375823000	19.990816000	23.940862000
C	13.102074000	20.326844000	24.363500000
N	13.221196000	18.103092000	23.915342000
N	14.452270000	18.614167000	23.663250000
C	10.956409000	18.815526000	24.659698000
C	12.359886000	19.126037000	24.339387000
C	15.540646000	20.846176000	23.633969000
H	15.156872000	21.780255000	23.192847000
H	16.093349000	20.299274000	22.851374000
I	12.545058000	22.278883000	24.843136000
N	7.659283000	20.508277000	29.217170000
C	8.461965000	20.683487000	28.102132000
I	9.431995000	22.489869000	27.719724000

H	8.620302000	23.021969000	32.322106000
C	9.169652000	22.073838000	32.284089000
H	11.215523000	17.818598000	28.536407000
C	12.027778000	18.496578000	28.255193000
C	8.324569000	19.502082000	27.338580000
C	13.100873000	18.016731000	27.473382000
C	10.279140000	19.439302000	25.736938000
H	10.799069000	20.189059000	26.334468000
C	8.788351000	21.076335000	31.353810000
S	15.579707000	20.798720000	30.537717000
C	15.173580000	19.145501000	31.390131000
H	15.150758000	18.379592000	30.599093000
C	8.975646000	19.020120000	26.107750000
C	11.998071000	19.844137000	28.673020000
H	11.166594000	20.201418000	29.286134000
C	12.048582000	20.351512000	34.154096000
S	13.879927000	20.496504000	33.493283000
H	12.023795000	19.313413000	34.524361000
H	12.041119000	21.054515000	35.000935000
C	16.472277000	21.158736000	24.798293000
C	14.139462000	20.233180000	27.561776000
H	14.965864000	20.903179000	27.305672000
C	13.048180000	20.727004000	28.322440000
C	7.578444000	21.306154000	30.448726000
H	7.474912000	22.371570000	30.184645000
H	6.650428000	20.988885000	30.954985000
C	13.807782000	19.246628000	32.049269000

H	13.514215000	18.280709000	32.491658000
H	13.029156000	19.576935000	31.343669000
C	10.238284000	21.847420000	33.178838000
H	10.508983000	22.620064000	33.905430000
C	10.952457000	20.620825000	33.161371000
C	9.496473000	19.849206000	31.335148000
H	9.207828000	19.052041000	30.642529000
C	14.163253000	18.892692000	27.136223000
H	15.007949000	18.520232000	26.546792000
C	10.559863000	19.622776000	32.230568000
H	11.059733000	18.648354000	32.235152000
S	12.743034000	24.199606000	33.229929000
C	12.301926000	25.041558000	31.562231000
H	11.619843000	25.875329000	31.792968000
H	13.240462000	25.431295000	31.150382000
C	16.702457000	22.507157000	25.157161000
H	16.109263000	23.302170000	24.691775000
C	11.605558000	24.057500000	30.619653000
S	12.406114000	22.320017000	30.528099000
H	11.619575000	24.464047000	29.596258000
H	10.569003000	23.863160000	30.927561000
C	13.036865000	22.183050000	28.721097000
H	12.322028000	22.775210000	28.126020000
H	14.033995000	22.634651000	28.656846000
C	18.292486000	15.627085000	33.150131000
N	19.559205000	15.813277000	33.730716000
C	18.152820000	14.945882000	31.851321000

C	19.084086000	13.931404000	31.496045000
H	19.851134000	13.632898000	32.217379000
C	17.089992000	14.726979000	29.636300000
C	17.333593000	16.239284000	33.989402000
I	15.215524000	16.258978000	33.947270000
N	18.056180000	16.775061000	35.038445000
C	17.603378000	17.719441000	36.062727000
H	16.646954000	17.365266000	36.482504000
H	18.356482000	17.700389000	36.869014000
N	19.437060000	16.523469000	34.879520000
C	18.030895000	13.716402000	29.295035000
H	17.979644000	13.249816000	28.306339000
H	19.737119000	12.542319000	29.956460000
C	19.018811000	13.324156000	30.223714000
C	17.158945000	15.336699000	30.915749000
H	16.472341000	16.151140000	31.162541000
H	15.977278000	18.925790000	32.110378000
C	17.994169000	20.831269000	33.811108000
C	17.473990000	21.853369000	34.643879000
H	18.450527000	21.101352000	32.852263000
C	13.920441000	25.498819000	34.001025000
H	14.200745000	26.209381000	33.206606000
H	13.347983000	26.023350000	34.781291000
C	15.135250000	24.804682000	34.613483000
S	16.220523000	24.084115000	33.218865000
H	14.858577000	23.991757000	35.304100000
H	15.759638000	25.543303000	35.142037000

C	17.650387000	23.290253000	34.252184000
H	17.739102000	23.959493000	35.122516000
H	18.521290000	23.435519000	33.595464000
C	16.886252000	21.491459000	35.884476000
H	16.471085000	22.268193000	36.536979000
C	17.995388000	19.486541000	34.234682000
H	18.447072000	18.726968000	33.590478000
C	17.469772000	19.136361000	35.503481000
C	16.882619000	20.145399000	36.307856000
H	16.454169000	19.881744000	37.281663000
C	18.109143000	21.868166000	29.895544000
S	19.866867000	21.517023000	29.251720000
H	18.193074000	22.448367000	30.826525000
C	17.230510000	20.139136000	25.435295000
H	17.053010000	19.088971000	25.174698000
C	17.719505000	22.840349000	26.080843000
H	17.903897000	23.891223000	26.325246000
C	17.421200000	20.525320000	30.108155000
H	17.865228000	19.943701000	30.931125000
H	17.407446000	19.924306000	29.186024000
C	18.533265000	21.833216000	26.659404000
C	19.732958000	22.253566000	27.482346000
H	19.766865000	23.349354000	27.584118000
H	20.674672000	21.913480000	27.015922000
C	18.246314000	20.472851000	26.356922000
H	18.847570000	19.679709000	26.815686000
H	17.599531000	22.477450000	29.134103000

H	8.486311000	16.620265000	23.682482000
I	11.747032000	16.119229000	33.561414000
Zn	14.489200000	22.560356000	32.049782000
F	15.927311000	24.040565000	28.137804000
F	15.295377000	26.411508000	30.626854000
F	17.258238000	25.050667000	29.964187000
F	13.987542000	25.420414000	28.784297000
F	16.165679000	26.530935000	28.277205000
F	15.078594000	23.953625000	30.441452000
P	15.643892000	25.269689000	29.346149000

Table S19: Cartesian Coordenates of **TeO**-$Li^+.I^-$

**TeO**-$Li^+.I^-$	x	y	z
C	9.764579000	17.158191000	24.287095000
C	11.055823000	17.622198000	23.957400000
H	11.623918000	17.159523000	23.144532000
Te	12.891655000	16.027091000	31.197620000
C	13.585645000	15.356814000	29.289932000
C	9.049240000	17.743922000	25.351899000
H	8.056338000	17.374252000	25.624761000
N	7.978257000	18.445442000	27.925368000
N	7.482259000	19.057445000	29.029281000
N	14.601915000	14.328642000	27.507713000
N	13.506933000	14.961229000	27.014040000
N	12.885530000	15.576057000	28.112666000
C	14.691643000	14.565532000	28.891523000
C	11.800766000	16.549998000	27.889943000

H	11.494740000	16.435939000	26.836414000
H	10.944422000	16.304339000	28.538747000
N	14.964286000	20.109801000	24.033123000
C	13.664012000	20.368154000	24.439815000
N	13.939477000	18.158146000	23.914605000
N	15.142262000	18.747963000	23.718535000
C	11.635509000	18.696486000	24.686211000
C	13.011579000	19.111354000	24.363448000
C	16.087715000	21.047338000	23.858135000
H	15.723713000	21.909628000	23.272256000
H	16.831820000	20.501507000	23.254617000
Te	13.027907000	22.312445000	24.940330000
N	8.089611000	20.319131000	29.084880000
C	8.971345000	20.505158000	28.028414000
Te	10.036401000	22.306187000	27.738340000
H	9.333909000	23.263599000	31.258143000
C	9.663751000	22.295769000	31.651558000
H	10.707052000	18.473281000	29.536364000
C	11.624604000	18.815141000	29.048400000
C	8.896296000	19.298122000	27.288797000
C	12.321486000	17.950421000	28.177567000
C	10.911610000	19.286152000	25.753487000
H	11.383458000	20.067755000	26.353648000
C	8.999600000	21.113274000	31.243705000
O	15.565438000	20.675961000	31.264920000
C	15.224552000	19.395390000	31.926977000
H	15.310055000	18.560789000	31.205114000

C	9.622136000	18.809280000	26.102347000
C	12.112964000	20.114474000	29.302518000
H	11.585766000	20.780275000	29.990518000
C	12.417767000	20.898042000	33.935786000
O	13.669078000	20.803820000	33.125014000
H	12.384475000	20.020233000	34.607643000
H	12.552821000	21.818259000	34.527589000
C	16.703072000	21.516784000	25.170360000
C	14.030745000	19.677472000	27.850455000
H	14.967474000	20.008606000	27.387175000
C	13.312612000	20.557373000	28.697702000
C	7.838938000	21.179390000	30.258655000
H	7.672061000	22.212160000	29.913972000
H	6.906265000	20.800897000	30.709101000
C	13.789093000	19.508874000	32.407016000
H	13.553744000	18.677150000	33.098034000
H	13.082798000	19.491367000	31.559360000
C	10.750428000	22.225740000	32.550407000
H	11.267440000	23.142602000	32.858215000
C	11.192262000	20.975877000	33.051212000
C	9.429278000	19.862219000	31.753631000
H	8.916411000	18.943660000	31.446644000
C	13.538738000	18.383455000	27.593191000
H	14.094775000	17.708686000	26.932766000
C	10.514776000	19.794328000	32.650907000
H	10.843487000	18.824768000	33.045572000
O	14.618113000	24.239595000	31.719120000

C	14.176962000	24.759622000	30.412793000
H	13.938250000	25.838397000	30.493928000
H	14.978673000	24.615041000	29.660556000
C	16.693965000	22.889843000	25.513531000
H	16.191147000	23.608576000	24.856198000
C	12.930795000	23.945750000	30.087447000
O	13.246759000	22.510182000	30.152953000
H	12.524841000	24.215372000	29.093574000
H	12.157061000	24.113817000	30.853757000
C	13.802846000	21.970649000	28.896013000
H	13.457727000	22.606952000	28.052842000
H	14.906812000	22.010226000	28.916498000
C	18.245496000	15.165003000	32.434398000
N	19.644921000	15.270660000	32.506495000
C	17.616155000	14.382990000	31.352214000
C	18.255174000	13.195684000	30.898133000
H	19.179006000	12.866684000	31.384476000
C	15.863641000	14.073626000	29.641784000
C	17.676205000	15.904514000	33.504447000
Te	15.669416000	16.220202000	34.165778000
N	18.771823000	16.432095000	34.168966000
C	18.800403000	17.246285000	35.399013000
H	18.366890000	16.649621000	36.221023000
H	19.868777000	17.417664000	35.612725000
N	19.986399000	16.055685000	33.556730000
C	16.511155000	12.883746000	29.207088000
H	16.082526000	12.315996000	28.375355000

H	18.191864000	11.534648000	29.495978000
C	17.699088000	12.451946000	29.835143000
C	16.419783000	14.811907000	30.720239000
H	15.943301000	15.743926000	31.040642000
H	15.927121000	19.232791000	32.764382000
C	17.723868000	20.740312000	34.169584000
C	16.607734000	21.004184000	35.001813000
H	18.032406000	21.490380000	33.435501000
C	15.761876000	24.951080000	32.311610000
H	16.676608000	24.745453000	31.722742000
H	15.573503000	26.041863000	32.326045000
C	15.864871000	24.421675000	33.730244000
O	15.942406000	22.947966000	33.635043000
H	14.965612000	24.709001000	34.311418000
H	16.768519000	24.818394000	34.232631000
C	15.887670000	22.332660000	34.979069000
H	14.829931000	22.214088000	35.281345000
H	16.374841000	23.025986000	35.696634000
C	16.204448000	20.030581000	35.947466000
H	15.328465000	20.215143000	36.579959000
C	18.427148000	19.521458000	34.285148000
H	19.281721000	19.317571000	33.629438000
C	18.041418000	18.558130000	35.251783000
C	16.923667000	18.824395000	36.080865000
H	16.607665000	18.078487000	36.817398000
C	17.353969000	22.095127000	30.492154000
O	18.709854000	22.088162000	29.912958000

H	17.398494000	22.560383000	31.493232000
C	17.345718000	20.592313000	26.035391000
H	17.344732000	19.525329000	25.784007000
C	17.346738000	23.339116000	26.685768000
H	17.342775000	24.406597000	26.936686000
C	16.883304000	20.644447000	30.613495000
H	17.594123000	20.064666000	31.232860000
H	16.792481000	20.164138000	29.621162000
C	18.034168000	22.426126000	27.521554000
C	18.842638000	22.930284000	28.708828000
H	18.571489000	23.980372000	28.940820000
H	19.923573000	22.893137000	28.483580000
C	18.009563000	21.043472000	27.194457000
H	18.529056000	20.330547000	27.843746000
H	16.658522000	22.674857000	29.855588000
H	9.319671000	16.334740000	23.719181000
C	15.132862000	14.151059000	33.798967000
H	14.177573000	13.987761000	34.321769000
H	15.017637000	13.973402000	32.721421000
H	15.915378000	13.502068000	34.221809000
C	12.272100000	14.001651000	31.708472000
H	11.421108000	13.711314000	31.073628000
H	13.119341000	13.315282000	31.562760000
H	11.971836000	14.024395000	32.768193000
C	11.099369000	22.147759000	23.962956000
H	10.440867000	21.487084000	24.544460000
H	10.684432000	23.168670000	23.926065000

H	11.242513000	21.762985000	22.941781000
C	8.475034000	23.154274000	26.471930000
H	7.547845000	23.276097000	27.052644000
H	8.834758000	24.139027000	26.128429000
H	8.308876000	22.490706000	25.609991000
Li	14.577437000	22.213516000	32.045770000
I	12.107479000	16.635234000	34.786917000
